# Rab10 coordinates SADS-CoV non-lytic egress through the ERGIC-TGN-lysosome trafficking pathway

**DOI:** 10.1371/journal.ppat.1014569

**Published:** 2026-09-01

**Authors:** Liaoyuan Zhang, Jiyu Zhang, Mei Xue, Mengyu Liu, Xinwei Sun, Dakai Liu, Miaomiao Zeng, Gengting Gu, Hongyan Shi, Xin Zhang, Jianfei Chen, Zhaoyang Ji, Da Shi, Li Feng

**Affiliations:** State Key Laboratory of Animal Disease Control and Prevention, Chinese Academy of Agricultural Sciences Harbin Veterinary Research Institute, Harbin, China; National University of Singapore, SINGAPORE

## Abstract

Swine acute diarrhea syndrome coronavirus (SADS-CoV) is a bat-originated alphacoronavirus that causes devastating enteric disease in neonatal piglets and possesses significant potential for cross-species transmission. While the early stages of the coronavirus life cycle have been extensively characterized, the host factors indispensable for virion assembly and subsequent export remain largely enigmatic. Here, by performing a genome-wide CRISPR-Cas9 knockout screen using a recombinant icSADS-CoV-GFP reporter virus, we identified the small GTPase Rab10 as a critical host dependency factor for SADS-CoV infection. Viral life cycle analysis revealed that Rab10 is not required for viral attachment, entry, or initial genome replication, but is essential for the virion transport and non-lytic egress. Rab10 deficiency markedly reduced the extracellular release of viral RNA, viral proteins, and infectious progeny, as well as the secretion of SADS-CoV virus-like particles. Confocal imaging showed that Rab10 and viral protein-positive intracellular structures were associated with LMAN1, TGN46, and LAMP1 positive compartments. These findings support a model in which Rab10 coordinates a virus-containing vesicles trafficking pathway associated with ERGIC-TGN-lysosome compartments. Mechanistically, Rab10 facilitates the loading of the viral envelope (E) protein into transport vesicles derived from the ERGIC. Rab10 associates with the SADS-CoV E protein, and mapping analyses implicated the C-terminal PDZ-binding motif, particularly residue V75, in efficient Rab10 association and viral release. Collectively, our findings identify Rab10 as a host regulator of SADS-CoV non-lytic egress and highlight the E-Rab10 interaction and the vesicular trafficking machinery as a potential target for developing antiviral strategies.

## Author summary

Coronaviruses, including the emerging SADS-CoV, are notorious for their ability to cross species barriers and cause severe diseases. While it is well-established how these viruses enter cells and replicate their genetic material, the mechanisms of non-lytic egress, which allows progeny virions to exit the host cell and infect neighboring cells without compromising host cell viability, remain poorly understood. Here, we employed a genome-wide CRISPR-Cas9 screening approach and identified Rab10 as a pivotal host factor regulating SADS-CoV infection. We discovered that while Rab10 is dispensable for viral entry and assembly, but instead promotes a later stage of virus trafficking and non-lytic release. This guide service is triggered by an association between Rab10 and the viral E protein. By identifying the specific amino acid V75 as viral site that interacts with Rab10, we have uncovered a critical host-pathogen interface within the SADS-CoV life cycle. These findings reveal how SADS-CoV uses host membrane trafficking machinery for efficient egress and identify the E–Rab10 interaction as a potential target for future antiviral development.

## Introduction

Coronaviruses (CoVs) are enveloped, positive-sense RNA viruses that extensively remodel host endomembrane systems to create specialized environments for replication and morphogenesis. SADS-CoV is a highly pathogenic, bat-originated alphacoronavirus first identified during a large-scale outbreak of fatal diarrhea in piglets in 2017 [1]. Belonging to the species *Rhinolophus* bat coronavirus HKU2 [2], SADS-CoV causes devastating clinical manifestations, including acute vomiting and watery diarrhea, with mortality rates exceeding 90% in neonatal piglets under 5 days of age [3]. It has also been detected via antigen- and antibody-based assays in countries such as South Korea and Vietnam [4,5]. Beyond its significant impact on the global swine industry, SADS-CoV possesses broad cell tropism and the ability to replicate efficiently in various human cell lines and organoids, highlighting its potent risk for zoonotic spillover [6]. Similar to other highly pathogenic coronaviruses, SADS-CoV co-opts host cellular machinery to facilitate its complex life cycle, although the molecular mechanisms that coordinate the late stages, which primarily encompass virion assembly and cellular egress, remain poorly understood.

The morphogenesis of coronaviral progeny is a complex process that initiates with the assembly of structural proteins at the endoplasmic reticulum-Golgi intermediate compartment (ERGIC) [7]. Traditionally, following budding, mature virions must navigate the host's secretory machinery to reach the extracellular space [8,9]. While conventional models suggest a reliance on the classic biosynthetic secretory pathway [10], recent evidence indicates that certain betacoronaviruses, such as SARS-CoV-2 [11], PHEV [12] and MHV [13], utilize Golgi-independent routes or lysosomal exocytosis for non-lytic egress. These findings highlight a sophisticated “arms race” where viruses evolve divergent strategies to exploit host vesicular transport while maintaining host cell integrity for maximal progeny dissemination. While progress has been made in understanding betacoronaviral exit, the host factors that coordinate these non-classical transport itineraries for other coronaviruses like SADS-CoV have yet to be fully elucidated.

The emergence of genome-scale forward genetic screening, specifically CRISPR knockout technology, has revolutionized our understanding of virus infection biology by enabling the unbiased dissection of host-pathogen interactions [14]. Compared to traditional loss-of-function platforms like RNA interference (RNAi), CRISPR-Cas9 screens offer superior specificity and efficiency, providing a high signal-to-noise ratio through the permanent disruption of target genes. This technology has been pivotally applied to the study of various coronaviruses, uncovering essential host dependency factors for SARS-CoV-2, MERS-CoV, and several porcine enteric coronaviruses such as PEDV and TGEV [15]. These screens have identified both virus-specific entry receptors and conserved “pan-coronavirus” host factors, such as *PLAC8* [16], *TMEM41B* [17], *TMEM198* [18], *YIPF5* [19], and *ZDHHC17* [20] in facilitating the infection of multiple coronaviruses, including SARS-CoV-2, SADS-CoV and PEDV.

In this study, we employed a genome-wide CRISPR-Cas9 knockout screen using an icSADS-CoV-GFP reporter virus to identify host dependency factors required for infection. We identified Rab10 as an essential host factor that is indispensable for SADS-CoV infection. Rab10 typically modulates transport from the *trans*-Golgi network (TGN) and endosomes to the plasma membrane [21,22], suggesting that it may be exploited during the late stages of the viral life cycle. We show that Rab10 activity is specifically concentrated during the intracellular trafficking and non-lytic egress. We further show that Rab10 associates with the SADS-CoV envelope (E) protein and that the C-terminal PDZ-binding motif-containing region of E, particularly residue V75. This interaction facilitates the loading of mature virions into a hybrid secretory itinerary involving the ERGIC-TGN-lysosome pathway for non-lytic egress. These findings identify a previously unrecognized role for Rab10 in alphacoronavirus trafficking and egress. It's suggested that the E-Rab10 interaction and the associated host trafficking warrant further investigation as potential antiviral targets.

## Results

### Genome-wide CRISPR knockout screen identified host dependency factors for SADS-CoV infection

**Fig 1:**
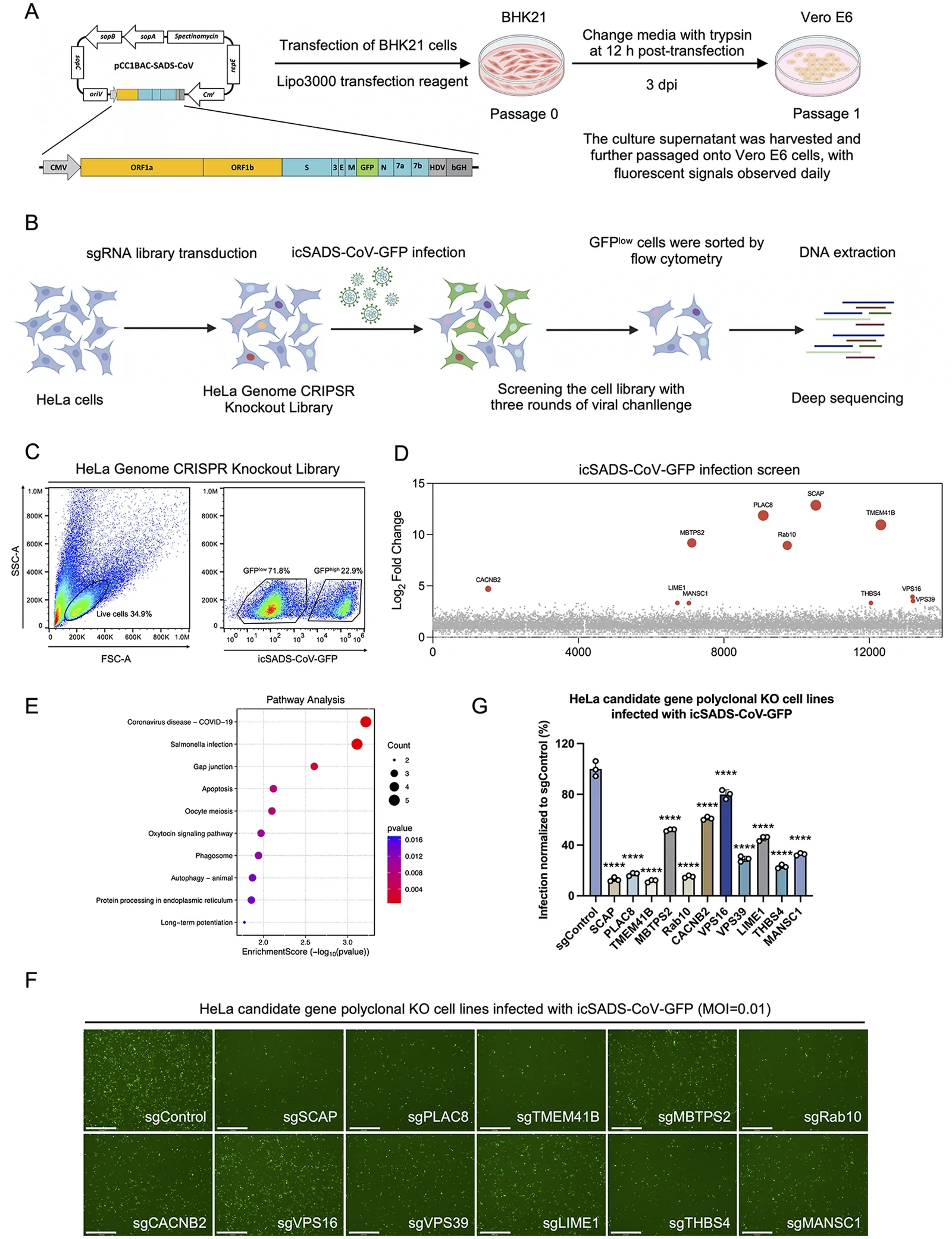
Genome-wide CRISPR-Cas9 screening identifies host factors using icSADS-CoV-GFP. **(A)** Schematic diagram of the icSADS-CoV-GFP rescue. The GFP-2A sequence was inserted between the M and N genes of the full-length SADS-CoV genome. Created with BioRender.com. https://BioRender.com/cc4lsyi **(B)** Schematic of the screening procedure. A CRISPR sgRNA knockout library was generated in HeLa cells. The library was infected with icSADS-CoV-GFP, and GFP negative cells were collected by fluorescence-activated cell sorting (FACS) for genomic DNA extraction and sequencing analysis. Created with BioRender.com. https://BioRender.com/vszss7g **(C)** Flow cytometry analysis after three rounds of icSADS-CoV-GFP infection and sorting. **(D)** Enrichment of sgRNAs in surviving cells after icSADS-CoV-GFP infection, as determined by NGS. The fold change in sgRNA reads was calculated by comparing surviving cells with the uninfected library control. The top 11 enriched genes are labeled. **(E)** KEGG pathway analysis of 50 top-ranked genes from the screens in (D). **(F)** Polyclonal HeLa knockout cell lines were established for the top 11 candidate genes and infected with icSADS-CoV-GFP. Green signal indicates GFP fluorescence. The lower panel shows representative images of positive cells from three independent experiments. Scale bar, 1250 μm. **(G)** Relative fluorescence intensity was quantified to indicate the percentage of infected cells. Data are presented as means ± SD from three independent experiments. Statistical significance was analyzed by one-way ANOVA with Dunnett's test. ^*^P < 0.05, ^**^P < 0.01, ^***^P < 0.001, ^****^P < 0.0001, ns, not significant.

Several CRISPR screening approaches have been employed to identify host factors involved in SADS-CoV infection, leading to the identification of genes such as *PLAC8* [16] and *ZDHHC17* [20]. These screenings were conducted by infecting CRISPR knockout cell libraries with wild-type SADS-CoV, followed by next-generation sequencing (NGS) analysis of surviving cells to identify candidate genes. To enhance the efficiency of this screening process, we designed and rescued a recombinant SADS-CoV reporter virus expressing green fluorescent protein (GFP) to replace the wild-type virus for cell infection (Fig 1A). This approach, combined with fluorescence-activated cell sorting (FACS), enables the isolation of resistant cell populations post-knockout, which are subsequently subjected to NGS analysis for candidate gene identification. Validation experiments demonstrated that icSADS-CoV-GFP exhibited growth kinetics, peak titers (up to 10^6^ TCID_50_/mL, TCID_50_: 50% tissue culture infectious dose) (S1A–B Fig), and plaque morphology comparable to the wild-type virus (S1C Fig). It consistently expressed both fluorescent and N proteins in infected HeLa cells (S1D–E Fig), while transmission electron microscopy (TEM) imaging confirmed the assembly of intact virions with typical spike structures (S1F Fig). These results establish icSADS-CoV-GFP as a robust tool for genome-wide screening.

To comprehensively identify novel host factors associated with SADS-CoV infection, we constructed a Human HeLa CRISPR GeCKO v2 library covering the whole genome. After infection with icSADS-CoV-GFP at an MOI (multiplicity of infection) of 0.01 for 48 h, GFP-negative cells were isolated by FACS and allowed to recover (Fig 1B–C). This process of viral infection and FACS-based isolation of GFP-negative cells was repeated for two additional rounds, resulting in a total of three rounds of selection. Genomic DNA was extracted from the GFP-negative cell population collected after the third infection, and NGS analysis was performed using primers specific to the GeCKO library to identify enriched sgRNAs. The screening results indicated that several host factors were highly enriched. The top-ranked host factors included *PLAC8*, *SCAP*, *MBTPS2*, *Rab10*, *TMEM41B*, *VPS16*, *CACNB2*, *VPS39*, *LIME1*, *MANSC1*, and *THBS4* (Fig 1D). The top 50 candidates were predominantly enriched in viral and bacterial infection pathways, confirming the successful identification of a robust host gene signature associated with pathogen-host interactions (Fig 1E). Subsequently, we constructed lentiCRISPR v2 knockout vectors for each of these selected genes. After lentiviral transduction of the knockout sgRNAs and one week of selection, polyclonal knockout HeLa cell lines were established for each gene. At 24 h post-infection (hpi) with icSADS-CoV-GFP (MOI = 0.01), GFP fluorescence signals were observed under a fluorescence microscope. The results showed significant differences in GFP signal intensity among the various polyclonal knockout cell lines after infection. Calculating the infection rate based on fluorescence intensity revealed the top three candidate genes to be *TMEM41B*, *SCAP*, and *Rab10* (Fig 1F–G). As the functions of the candidate genes *TMEM41B* [23] and *SCAP* [16] have been investigated in previous studies, we therefore selected *Rab10* for further analysis, whose role in coronavirus infection warranted mechanistic exploration.

### Rab10 is an important proviral host factor in SADS-CoV infection

**Fig 2:**
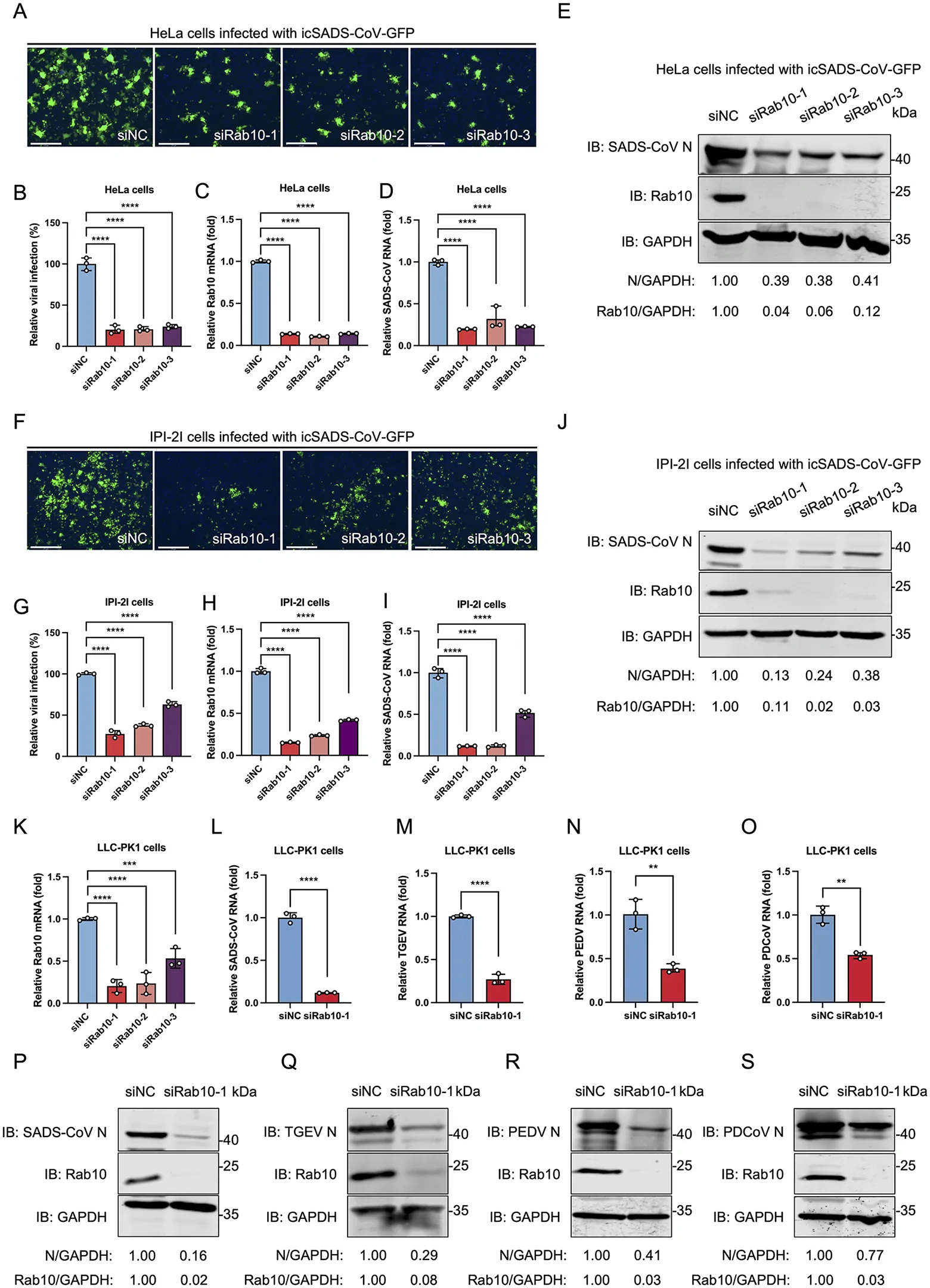
Rab10 is an important proviral host factor in pan-coronavirus infection. **(A–E)** Effect of Rab10 knockdown on SADS-CoV infection in HeLa cells. HeLa cells were transfected with siRNAs targeting Rab10 (siRab10–1, siRab10–2, and siRab10–3) or a negative control (siNC) for 24 h, followed by infection with icSADS-CoV-GFP at an MOI of 0.1. Samples were harvested at 24 hpi. Representative immunofluorescence images of HeLa cells infected with icSADS-CoV-GFP (green) (A) and quantification of relative viral infection rates (B). Nuclei were stained with DAPI (blue). Scale bar, 275 μm. Relative Rab10 mRNA levels determined by RT-qPCR (C). Intracellular SADS-CoV genomic RNA levels determined by RT-qPCR (D). Western blot analysis of SADS-CoV N protein and Rab10 expression (E). **(F–J)** Effect of Rab10 knockdown on SADS-CoV infection in IPI-2I cells. Representative immunofluorescence images (F) and quantification of viral infection in IPI-2I cells (G). Nuclei were stained with DAPI (blue). Scale bar, 275 μm. Relative Rab10 mRNA levels determined by RT-qPCR (H). Intracellular SADS-CoV genomic RNA levels determined by RT-qPCR (I). Western blot analysis of SADS-CoV N protein and Rab10 expression (J). **(K–O)** Effect of Rab10 knockdown on swine enteric coronaviruses infection in LLC-PK1 cells. Relative Rab10 mRNA levels determined by RT-qPCR (K). Relative SADS-CoV mRNA levels determined by RT-qPCR (L). Relative mRNA levels of TGEV (M), PEDV (N), and PDCoV (O) in siRab10-treated cells compared to control. **(P–S)** Western blot analysis of viral N protein and Rab10 expression in siNC or siRab10–1 treated LLC-PK1 cells infected with SADS-CoV (P), TGEV (Q), PEDV (R) or PDCoV (S). Data points represent individual biological replicates (n = 3). Data are presented as means ± SD from three independent experiments. B–D, G**–**I, K, one-way ANOVA with Dunnett's test; L–O, unpaired t-test. ^*^P < 0.05, ^**^P < 0.01, ^***^P < 0.001, ^****^P < 0.0001, ns, not significant.

To further investigate the role of Rab10 in SADS-CoV infection, we employed RNAi as an orthogonal approach to validate its function. HeLa cells were transfected with three independent Rab10-specific siRNAs and subsequently infected with icSADS-CoV-GFP for 24 h. Rab10-knockdown cells exhibited a marked reduction in the GFP-positive infection signal, with an average decrease of 78.39% compared with siNC-treated cells (Fig 2A–B). RT-qPCR analysis showed that the three siRNAs reduced Rab10 mRNA levels by an average of 87.28% (Fig 2C). Consistently, intracellular SADS-CoV RNA levels were reduced by an average of 75.32% (Fig 2D). Western blot analysis further confirmed efficient Rab10 depletion at the protein level and revealed a corresponding reduction in SADS-CoV N protein abundance (Fig 2E). Densitometric analysis indicated average reductions of 92.67% in Rab10 protein levels and 60.67% in viral N protein levels. Collectively, these results demonstrate that Rab10 is required for efficient SADS-CoV infection.

Given that SADS-CoV infection primarily causes intestinal diarrhea in piglets by targeting the porcine small intestinal epithelium, we performed RNAi in the porcine ileal epithelial cell line IPI-2I. Cells were transfected with three porcine Rab10-specific siRNAs and subsequently infected with icSADS-CoV-GFP for 24 h. Rab10 depletion significantly reduced the GFP-positive infection signal, with an average decrease of 57.42% compared with siNC-treated cells (Fig 2F–G). The three siRNAs reduced Rab10 mRNA levels by 57.49%–84.78% (Fig 2H), while intracellular SADS-CoV RNA levels decreased by an average of 74.84% (Fig 2I). Western blot analysis further confirmed efficient depletion of Rab10 protein and a corresponding reduction in viral N protein abundance (Fig 2J). Densitometric analysis showed average reductions of 94.67% in Rab10 protein levels and 75.00% in SADS-CoV N protein levels. These results confirm that Rab10 is also required for SADS-CoV infection in porcine intestinal epithelial cells. To further assess the impact of Rab10 on the replication of diverse porcine coronaviruses, we employed swine LLC-PK1 cells, which are susceptible to SADS-CoV, TGEV, PEDV, and PDCoV. Following RNAi mediated knockdown of Rab10 in these cells, three distinct siRNAs reduced Rab10 mRNA levels by 46.71% to 79.54% (Fig 2K). At 24 hpi with the respective porcine coronaviruses, intracellular viral genome levels were quantified by RT-qPCR. Treatment with the most effective siRNA (siRab10–1) resulted in an average reduction of 88.18% for SADS-CoV (Fig 2L), 72.95% for TGEV (Fig 2M), 61.62% for PEDV (Fig 2N), and 45.72% for PDCoV replication (Fig 2O). Western blot further confirmed efficient Rab10 depletion and corresponding reductions in viral N protein levels (Fig 2P–S). These results suggest that Rab10 broadly promotes infection by multiple porcine coronaviruses.

To determine whether SADS-CoV regulates endogenous Rab10, we examined Rab10 expression and localization in infected HeLa cells. Rab10 mRNA levels remained unchanged from 12 to 48 hpi, while total Rab10 protein was not increased and declined at later time points (S2A–B Fig), suggesting that SADS-CoV primarily utilizes the pre-existing Rab10 pool rather than inducing its expression. Immunofluorescence analysis further showed that Rab10 displayed a dispersed punctate cytoplasmic pattern in mock-infected cells, whereas a subset of Rab10-positive structures overlapped with SADS-CoV S-positive puncta following infection, particularly near the cell membrane (S2C Fig). These results indicate that SADS-CoV alters the subcellular distribution of Rab10 without increasing its overall expression.

**Fig 3:**
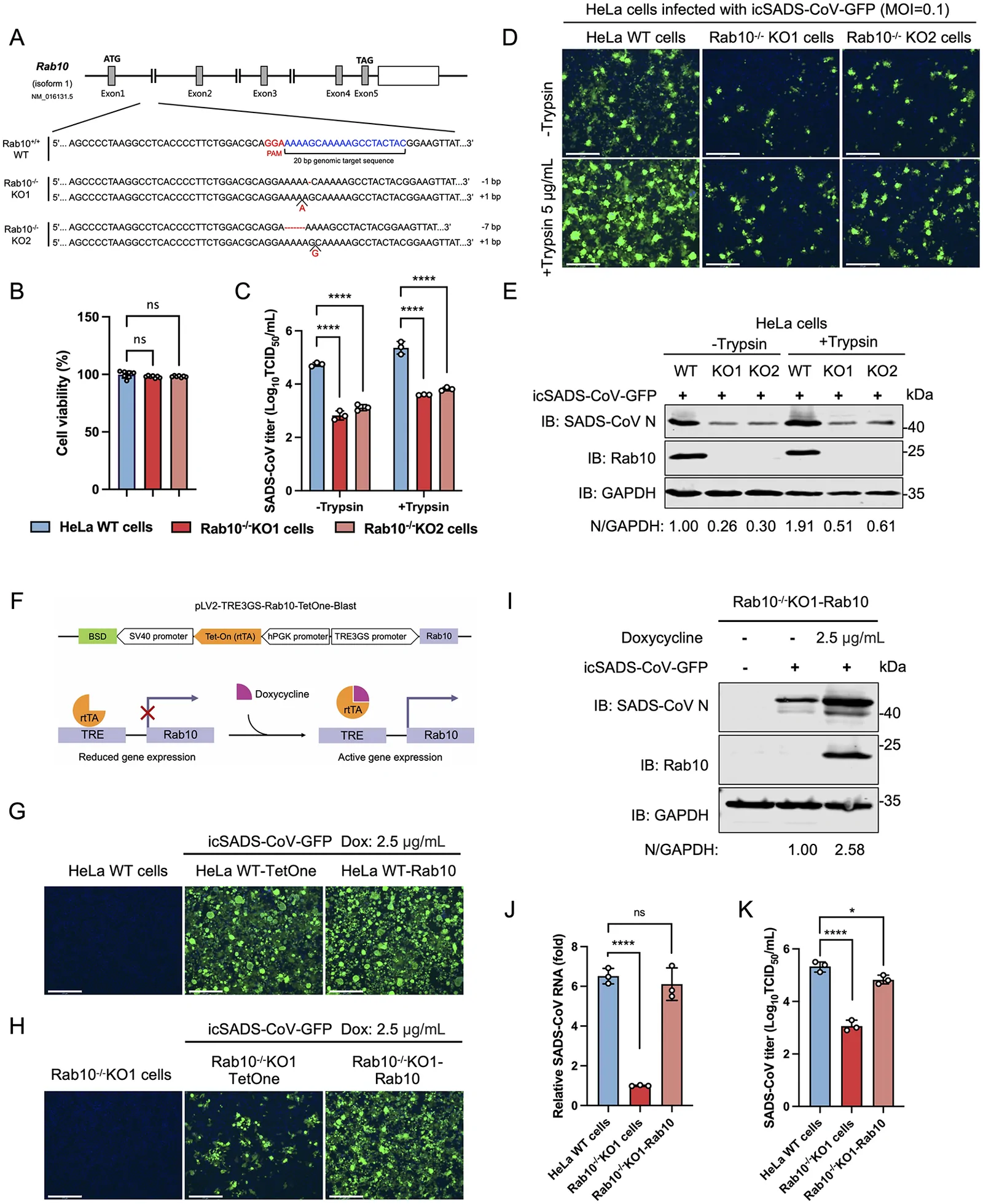
SADS-CoV replication was inhibited in Rab10 knockout cells and restored by Rab10 reconstitution. **(A)** Strategy for generating Rab10 knockout HeLa cell lines using CRISPR-Cas9. The schematic shows the sgRNA targeting exon 2 of the Rab10 gene. Sanger sequencing alignments confirm frameshift mutations (deletions/insertions) in two independent clones (KO1 and KO2) compared to WT sequences. **(B)** Cell viability of WT and Rab10 KO HeLa cells. Data show no significant cytotoxicity associated with Rab10 deletion. **(C–E)** Effect of Rab10 deficiency on SADS-CoV infection. WT and Rab10 KO cells were infected with icSADS-CoV-GFP (MOI = 0.1) in the presence or absence of trypsin. Viral titers in cell supernatants determined by TCID_50_ assay (C). Representative fluorescence microscopy images of cells infected with icSADS-CoV-GFP (green) (D). Nuclei were stained with DAPI (blue). Scale bar, 275 μm. Immunoblot analysis of SADS-CoV N protein and Rab10 expression at 24 hpi (E). GAPDH served as a loading control. Band intensities were quantified and normalized to GAPDH (values shown below bands). **(F)** Schematic of the Doxycycline (Dox)-inducible Tet-On system (pLV2-TRE3GS-Rab10-TetOne-Blast) used for Rab10 expression. Binding of the rtTA protein to the TRE promoter in the presence of Dox triggers active gene expression. Created with BioRender.com. https://BioRender.com/i4o2s2d **(G)** Representative fluorescence microscopy images of icSADS-CoV-GFP (green) infection in HeLa WT cells, HeLa-WT-TetOne (empty vector), and HeLa-TetOne-Rab10 cells following induction with 2.5 μg/mL Dox. Nuclei were stained with DAPI (blue). Scale bar, 275 μm. **(H)** Reconstitution of Rab10 in *Rab10*^*-/-*^ KO1 cells. *Rab10*^*-/-*^ KO1 cells were transduced with TetOne-empty or TetOne-Rab10 and induced with Dox before infection with icSADS-CoV-GFP (green). Representative fluorescence microscopy images of the infected cells. Nuclei were stained with DAPI (blue). Scale bar, 275 μm. **(I)** Western blot verification of Rab10 rescue in *Rab10*^*-/-*^ KO1 cells. The levels of SADS-CoV N, Rab10, and GAPDH were detected following Dox induction and viral infection. GAPDH was used as a loading control. Band intensities were quantified using Image J and normalized to GAPDH. N/GAPDH ratios are provided. **(J)** Quantitative analysis of SADS-CoV vRNA (left axis, bars) and viral titers (right axis, dots) in HeLa WT, *Rab10*^*-/-*^ KO1, and Rab10-reconstituted cells. Data points represent individual biological replicates (n = 3). Data are presented as means ± SD from three independent experiments. B–C, J-K, one-way ANOVA with Dunnett's test. ^*^P < 0.05, ^**^P < 0.01, ^***^P < 0.001, ^****^P < 0.0001, ns, not significant.

To further elucidate the function of Rab10 in SADS-CoV infection, we generated Rab10-knockout (KO) cell lines using the CRISPR-Cas9 genome-editing system. Sequencing revealed various deletions or insertions near the sgRNA target site in both alleles of two independent KO clones, resulting in complete loss of Rab10 protein (Fig 3A). Cell viability, assessed by the CCK-8 (Cell Counting Kit-8) assay, was 98.75% and 96.62% for the KO clones compared to wild-type (WT) cells, indicating that Rab10 knockout had a relatively small impact on the normal life activities of cells (Fig 3B). We then infected the KO clones (KO1 and KO2) and WT HeLa cells with icSADS-CoV-GFP in the presence of exogenous trypsin. At 24 hpi, TCID_50_ assay of infectious virus particles in the supernatant revealed that KO cells produced significantly fewer infectious virions than WT cells (Fig 3C), and both GFP fluorescence and intracellular N protein level were markedly reduced in KO1 and KO2 cells compared to WT controls (Fig 3D–E). This reduction in viral infection was observed both with and without exogenous trypsin, suggesting that Rab10's role is independent of trypsin-mediated enhancement of viral entry. These results demonstrate that Rab10 knockout substantially impairs SADS-CoV infection. To determine whether Rab10 is essential for SADS-CoV infection, we performed a complementation assay by re-expressing Rab10 in the knockout cells. The stable overexpression of Rab10 via a doxycycline-inducible system in WT cells enhanced viral infection to some extent (Fig 3F). Importantly, restoring Rab10 expression in KO1 cells (rescue experiment) significantly recovered viral infectivity (Fig 3G–K). Collectively, these results confirm that Rab10 is an important proviral host factor for SADS-CoV infection and exhibits a similar facilitatory role in the replication of other porcine coronaviruses.

### Rab10 is essential for the late-stage of SADS-CoV in a single-cycle infection

**Fig 4:**
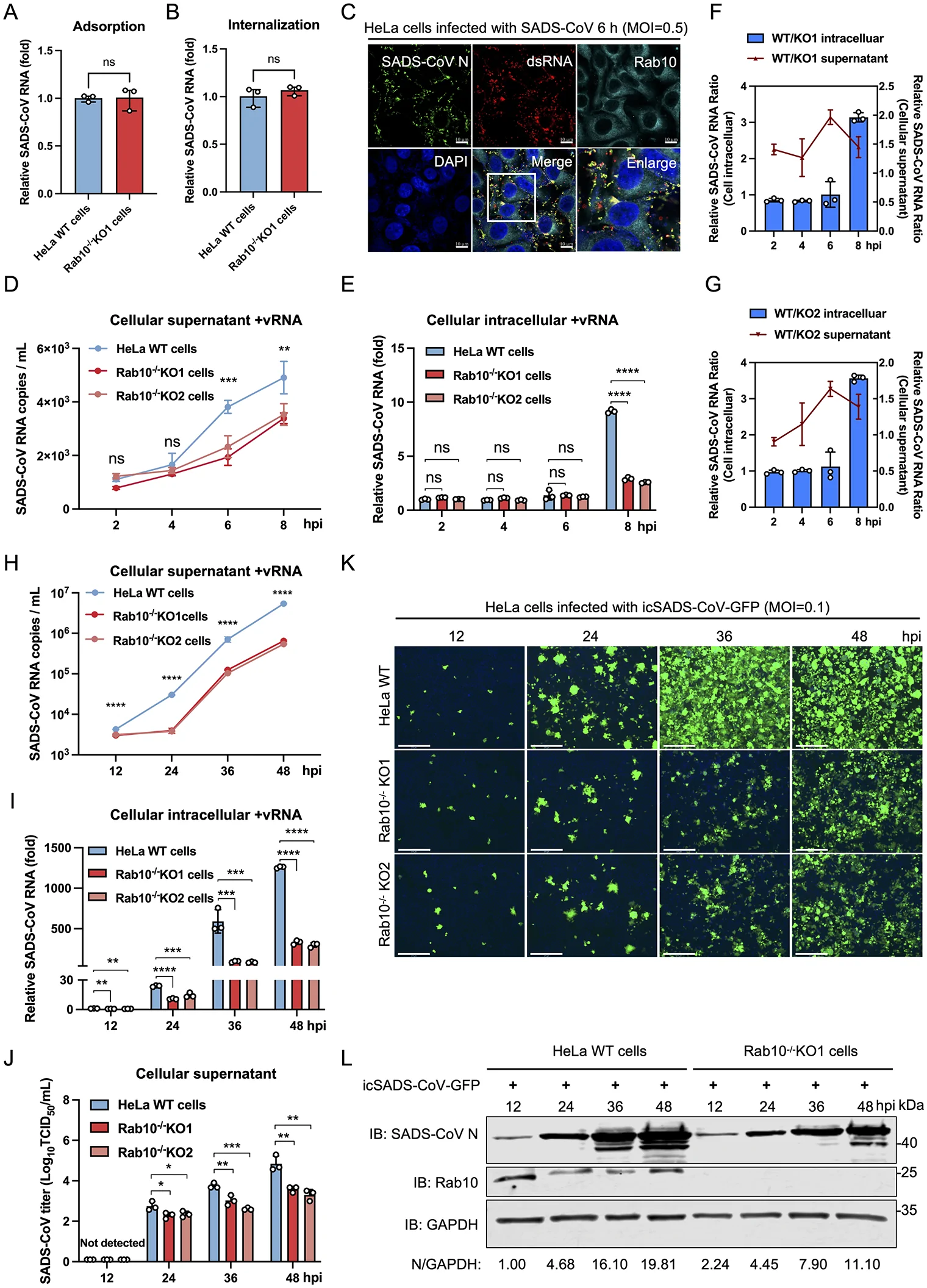
Rab10 is essential for the late-stage assembly or egress of SADS-CoV in a single-cycle infection. **(A–B)** Effect of Rab10 knockout on viral attachment and internalization. HeLa WT and *Rab10*^*-/-*^ KO1 cells were inoculated with SADS-CoV. Viral adsorption was determined by RT-qPCR of cell-bound viral RNA after incubation at 4°C (A). Viral internalization was assessed by quantifying intracellular viral RNA after incubation at 37°C and removal of surface virus (B). ns, not significant. **(C)** Confocal microscopy analysis of SADS-CoV replication complexes in HeLa WT cells. HeLa cells infected with SADS-CoV (MOI = 0.5) for 6 h were immunostained for N protein (green), dsRNA (red), and Rab10 (cyan). Nuclei were stained with DAPI (blue). Scale bar, 10 μm. **(D–E)** Single-cycle viral replication kinetics. SADS-CoV RNA copies in the cellular supernatant (D) and intracellular fold change (E) were measured from 2 to 8 hpi in HeLa WT, *Rab10*^*-/-*^ KO1, and *Rab10*^*-/-*^ KO2 cells. **(F–G)** Relative SADS-CoV RNA ratios comparing WT/KO2 (F) and WT/KO1 (G) in both intracellular and supernatant compartments across early time points (2–8 h). **(H–I)** Long-term viral growth kinetics. Viral RNA copies in the cellular supernatant (H) and intracellular vRNA fold change (I) were quantified via RT-qPCR from 12 to 48 hpi. **(J)** Determination of infectious progeny virus titers. Cellular supernatants from WT, KO1, and KO2 cells were collected at indicated time points, and titers were determined by TCID_50_ assay. **(K)** Representative fluorescence images showing the progression of icSADS-CoV-GFP (green) infection (MOI = 0.1) in HeLa WT, *Rab10*^*-/-*^ KO1, and *Rab10*^*-/-*^ KO2 cells from 12 to 48 hpi. Nuclei were stained with DAPI (blue). Scale bar, 275 μm. **(L)** Western blot analysis of viral protein expression. HeLa WT and *Rab10*^*-/-*^ KO1 cells were infected with icSADS-CoV-GFP, and the levels of SADS-CoV N and Rab10 were detected at indicated time points. GAPDH served as a loading control. Relative N/GAPDH ratios are provided below the blots. Data are presented as means ± SD from three independent experiments. A–B, unpaired t-test; D–E, H–J, one-way ANOVA with Dunnett's test. ^*^P < 0.05, ^**^P < 0.01, ^***^P < 0.001, ^****^P < 0.0001, ns, not significant.

To determine the role of Rab10 in the SADS-CoV infection cycle, we first assessed whether the adsorption and internalization stages differ between HeLa WT and *Rab10*^*-/-*^ KO1 cells. For the adsorption assay, cells were incubated with SADS-CoV (MOI = 0.5) at 4°C for 2 h. For the internalization assay, cells were first allowed to adsorb virus at 4°C for 2 h and then shifted to 37°C for 1 h to permit entry. Viral RNA levels were quantified by RT-qPCR, revealing no significant differences between HeLa WT and *Rab10*^-/-^ KO1 cells in either adsorption or internalization (Fig 4A–B). These results indicate that Rab10 knockout is dispensable for viral particle adsorption or internalization. Confocal microscopy was performed to examine the spatial relationship between Rab10 and dsRNA-labeled viral RNA replication sites in HeLa WT cells at 6 hpi with SADS-CoV. The results revealed that Rab10 did not strongly colocalize with the viral RNA replication sites, which were marked by both the viral N protein and dsRNA (Fig 4C). This suggests that Rab10 is likely not involved in the viral RNA replication process. To pinpoint which stage of the infection cycle is impaired upon Rab10 depletion, we monitored viral RNA levels at 2 h intervals from 0 to 8 hpi. Intracellular vRNA was measured by relative quantification, while released viral RNA in the supernatant was measured by absolute quantification. Starting at 6 hpi, supernatant vRNA levels were significantly reduced in *Rab10*^*-/-*^ KO (KO1 and KO2) cells compared to WT cells (Fig 4D). Intracellular vRNA showed no difference at 6 h but became significantly reduced in KO cells at 8 h compared with WT cells (Fig 4E). A comparative analysis of the viral RNA ratio between WT and KO cells revealed that WT cells released more viral RNA into the supernatant by 6 h, and by 8 h the newly produced virions led to higher intracellular vRNA accumulation in WT cells (Fig 4F–G). These observations suggest that Rab10 is likely involved in the virus late-stage of the infection cycle. Furthermore, we performed long-term propagation assays (12–48 h) following icSADS-CoV-GFP infection. Analysis revealed that Rab10 deficiency significantly reduced intracellular viral RNA levels, supernatant vRNA, and infectious virus titers compared to control cells (Fig 4H–J). Consistently, Rab10 knockout delayed SADS-CoV infection, as evidenced by the distinct differences in GFP fluorescence and N protein levels observed at 36 h (Fig 4K–L). Systematic dissection of the SADS-CoV life cycle reveals that Rab10 is dispensable for viral entry and primary replication, but essential for the efficient egress of progeny virions. Consequently, Rab10 acts as a critical proviral factor by facilitating late-stage viral release and subsequent spread.

### Rab10 is co-released with SADS-CoV virions as a vesicular component to facilitate non-lytic egress

**Fig 5:**
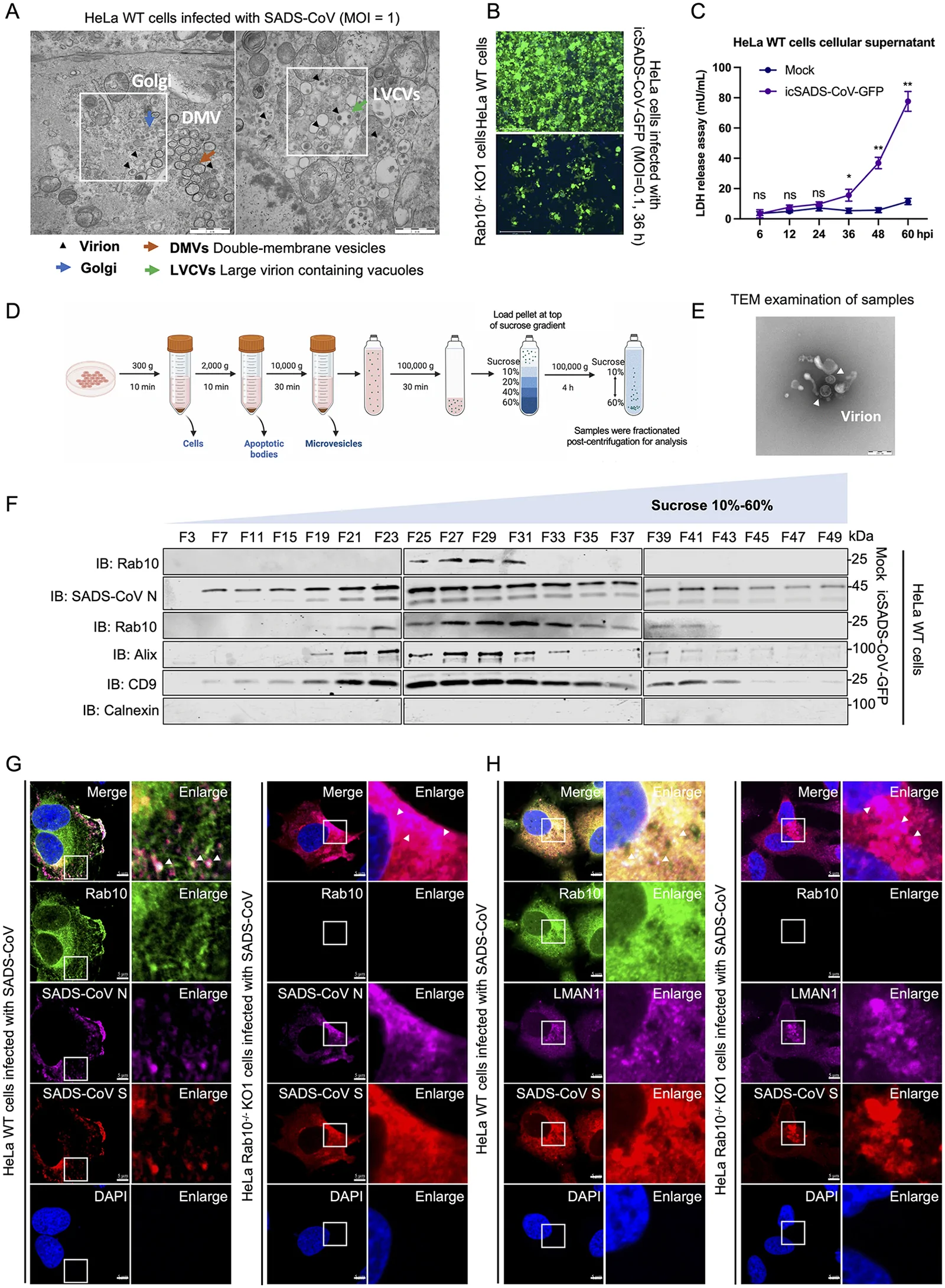
Rab10 is co-detecteded with SADS-CoV virions in extracellular vesicle enriched fractions as a vesicular component to facilitate non-lytic egress. **(A)** TEM analysis of intracellular ultrastructures in SADS-CoV-infected HeLa cells. Notable structures identified include the Golgi apparatus (blue arrow), double-membrane vesicles (DMVs, orange arrow), individual virions (black triangles), and large virion-containing vacuoles (LVCVs, green arrow). Scale bar, 1 μm. **(B)** Representative fluorescence microscopy images of icSADS-CoV-GFP infection (MOI = 0.1) in HeLa WT and *Rab10*^*-/-*^ KO1 cells at 36 hpi. Nuclei were stained with DAPI (blue). Scale bar, 275 μm. **(C)** Lactate dehydrogenase (LDH) release in the culture supernatants of HeLa cells following mock treatment or icSADS-CoV-GFP infection. Supernatants were collected at the indicated time points. Data are presented as means ± SD from three independent experiments used unpaired t-test. ^*^P < 0.05, ^**^P < 0.01, ^***^P < 0.001, ^****^P < 0.0001, ns, not significant. **(D)** Schematic workflow of the differential centrifugation and sucrose gradient ultracentrifugation protocol used to isolate and purify virions and extracellular vesicles from the cell culture supernatant. Created with BioRender.com. https://BioRender.com/su1lhy7 **(E)** Negative-stain TEM image of purified SADS-CoV virions obtained from the sucrose gradient fractions. Scale bar, 200 nm. **(F)** Sucrose gradient fractionation analysis of extracellular components. Concentrated supernatants from uninfected (top) or icSADS-CoV-GFP infected (bottom) HeLa cells were fractionated through a 10–60% sucrose gradient. Collected fractions (F3–F49) were analyzed by western blot using antibodies against Rab10, CD9, SADS-CoV N protein, Alix, and Calnexin to determine the co-fractionation profile of the virus and host factors. **(G)** Representative confocal microscopy images of SADS-CoV-infected HeLa WT and *Rab10*^*-/-*^ KO1 cells stained for Rab10 (green), the viral nucleocapsid protein (SADS-CoV N, purple), and the viral spike protein (SADS-CoV S, red). Nuclei were stained with DAPI (blue). Scale bar, 5 μm. Boxed regions are enlarged in the right-hand panels, and arrowheads indicate representative areas of signal overlap or close spatial association. **(H)** Representative Confocal microscopy images images of SADS-CoV-infected HeLa WT and *Rab10*^*-/-*^ KO1 cells stained for Rab10 (green), LMAN1 (purple), and SADS-CoV S (red). Nuclei were stained with DAPI (blue). Scale bar, 5 μm. Boxed regions are enlarged, and arrowheads indicate representative areas of signal overlap or close spatial association.

Numerous ultrastructural studies have demonstrated that coronaviruses co-opt the host endomembrane trafficking system for assembly and egress [24]. Coronavirus assembly occurs at the ERGIC, where the N, M, and E proteins coalesce and associate with the viral RNA to form virions [25]. This process leads to the formation of large virion-containing vacuoles (LVCVs), which are large, circular organelles that are thought to originate from Golgi cisternae and which are subsequently transported to the vicinity of the plasma membrane [26]. The TEM analysis of SADS-CoV-infected HeLa cells revealed intact virions sequestered within tubular membranous structures and large LVCVs, alongside characteristic double-membrane vesicles (DMVs) (Fig 5A). These ultrastructural features suggest a vesicle-mediated, non-lytic dissemination mechanism.

To investigate how Rab10 participates in SADS-CoV release, we collected culture supernatants from icSADS-CoV-GFP infected HeLa WT and *Rab10*^-/-^ KO1 cells at 36 hpi (Fig 5B). An LDH-release assay showed that plasma membrane integrity was largely maintained at 36 hpi, the time point used for supernatant collection, whereas substantial LDH release was primarily observed at later stages of infection (Fig 5C), thus supernatants were carefully harvested before significant cell detachment or lysis to minimize contamination by intracellular components. To further characterize extracellular membrane-associated components, culture supernatants were subjected to sequential centrifugation followed by sucrose-density-gradient fractionation (Fig 5D). Transmission electron microscopy of the isolated material revealed virion-like particles (Fig 5E). Western blot analysis of the gradient fractions revealed that under basal conditions, secreted Rab10 was detected in extracellular fractions containing SADS-CoV N protein and the extracellular vesicle-associated markers Alix and CD9, whereas the intracellular contamination marker Calnexin was not detected (Fig 5F). Rab10 was primarily found in fractions F25–F31. Following SADS-CoV infection, both the amount of secreted Rab10 increased and its density profile shifted downward to fractions F27–F35, which coincided with the peak fractions for SADS-CoV N protein. This co-sedimentation suggests that Rab10 functions as a component of the transport vesicles encapsulating the virions.

We next used confocal microscopy to investigate whether Rab10 participates in the intracellular trafficking of SADS-CoV. To visualize putative mature virion-associated structures, cells were co-stained with antibodies against the viral N and S proteins. In wild-type cells, Rab10-positive puncta overlapped with intracellular structures positive for both SADS-CoV N and S proteins. In contrast, *Rab10*^-/-^ KO1 cells exhibited pronounced intracellular accumulation of N- and S-positive signals, suggesting impaired transport of virus-associated structures (Fig 5G). We further examined the localization of SADS-CoV relative to the ERGIC, the primary site of coronavirus assembly. In wild-type cells, Rab10 and SADS-CoV S-positive structures showed prominent punctate association with the ERGIC marker LMAN1. In *Rab10*^-/-^ KO1 cells, the viral protein- and LMAN1-positive signals accumulated within more restricted intracellular regions, with fewer dispersed punctate structures, further indicating defective trafficking of virus-associated compartments (Fig 5H).

**Fig 6:**
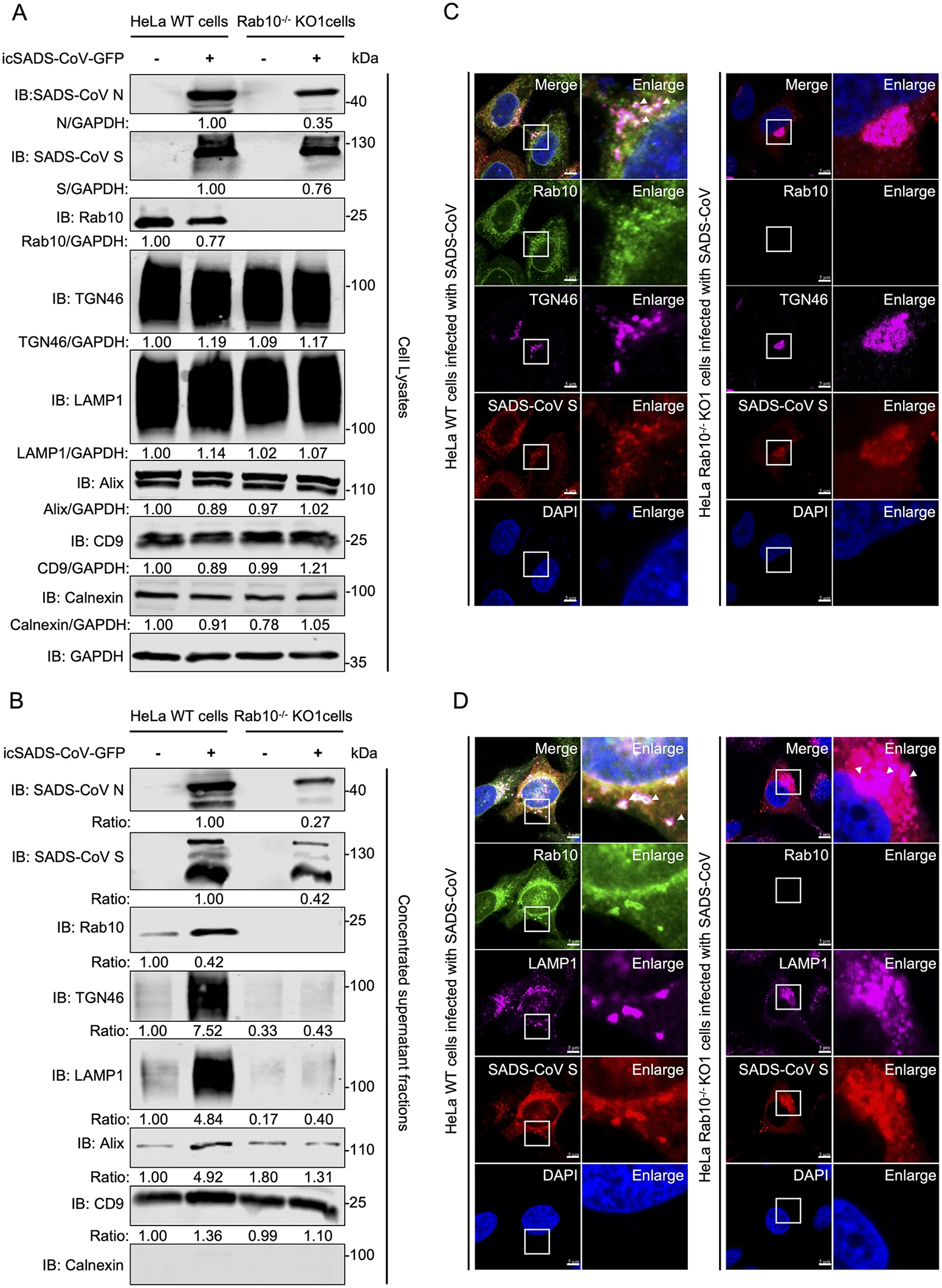
Rab10 deficiency impairs the release of SADS-CoV through TGN/lysosome-associated trafficking pathway. **(A–B)** Western blot analysis of viral and host protein levels in cell lysates (A) and concentrated culture supernatants (B). HeLa WT and *Rab10*^*-/-*^ KO1 cells were infected with SADS-CoV. Lysates and supernatants were harvested and immunoblotted for viral proteins (S, N) and host markers (Rab10, TGN46, LAMP1, Alix, CD9, Calnexin). GAPDH served as an internal loading control for cell lysates. For concentrated supernatant fractions, relative band intensities were expressed relative to the indicated control sample, which was set to 1.00. Calnexin was included as a marker of contamination by intracellular membranes or cellular contents. **(C)** Representative Confocal microscopy images images of SADS-CoV-infected HeLa WT and *Rab10*^*-/-*^ KO1 cells stained for Rab10 (green), TGN46 (purple), and SADS-CoV S (red). Nuclei were stained with DAPI (blue). Scale bar, 5 μm. Boxed regions are enlarged, and arrowheads indicate representative areas of signal overlap or close spatial association. **(D)** Representative Confocal microscopy images images of SADS-CoV-infected HeLa WT and *Rab10*^*-/-*^ KO1 cells stained for Rab10 (green), LAMP1 (purple), and SADS-CoV S (red). Nuclei were stained with DAPI (blue). Scale bar, 5 μm. Boxed regions are enlarged, and arrowheads indicate representative areas of signal overlap or close spatial association.

We next investigated whether Rab10 participates in the post-assembly release of SADS-CoV particles from the ERGIC. Total ultracentrifugation-concentrated supernatants and corresponding cell lysates were analyzed by western blot for the viral proteins N and S, Rab10, and compartment-specific markers, including TGN46 for the trans-Golgi network, LAMP1 for lysosomes, and Alix and CD9 for exosomes/extracellular vesicles. In cell lysates, Rab10 knockout reduced the abundance of SADS-CoV N and S proteins, whereas the total cellular levels of TGN46, LAMP1, Alix, CD9, and Calnexin remained largely unchanged (Fig 6A). In concentrated supernatants from infected wild-type cells, SADS-CoV N and S proteins were detected together with Rab10, TGN46, LAMP1, Alix, and CD9. Infection increased the extracellular abundance of Rab10, TGN46, and LAMP1. In contrast, Rab10 knockout reduced the extracellular abundance of the viral proteins and markedly decreased extracellular TGN46 and LAMP1 levels (Fig 6B). These findings suggest that SADS-CoV may exploit Golgi- and lysosome-associated pathways for non-lytic egress.

Confocal microscopy further revealed overlapping punctate signals among Rab10, SADS-CoV S, and TGN46 in wild-type cells (Fig 6C). Similarly, Rab10- and SADS-CoV S-positive structures overlapped with LAMP1-positive compartments (Fig 6D). In *Rab10*^-/-^ KO1 cells, SADS-CoV S-positive signals accumulated within compact TGN46- or LAMP1-positive intracellular regions, accompanied by a reduction in dispersed punctate structures. Collectively, these results suggest that SADS-CoV exploits the host endomembrane system for non-lytic egress and that Rab10 functions as a key trafficking factor during this process. Rab10 facilitates the transport of SADS-CoV associated vesicular structures through an ERGIC–TGN/lysosome associated pathway, thereby promoting the extracellular release of viral and vesicular components.

### Rab10 promotes the release of coronavirus VLPs through TGN and lysosome associated vesicular trafficking

**Fig 7:**
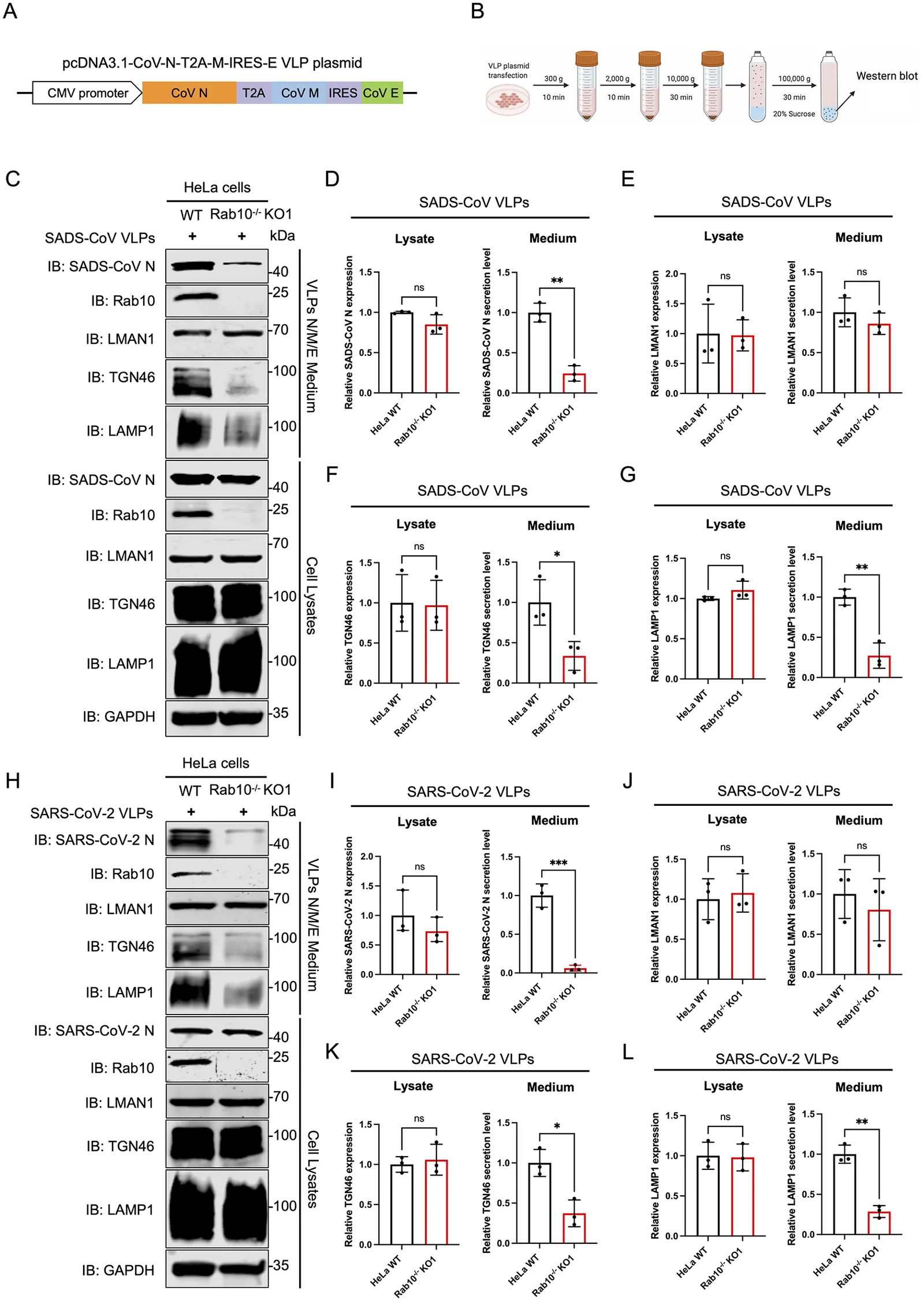
Rab10 is essential for the efficient secretion of coronavirus virus-like particles. **(A)** Schematic representation of the VLPs expression plasmid (pcDNA3.1-CoV-N-T2A-M-IRES-E) containing a CMV promoter, SADS-CoV or SARS-CoV-2 N gene, a T2A self-cleaving peptide, the M gene, an Internal Ribosome Entry Site (IRES), and the E gene. Created with BioRender.com. https://BioRender.com/qse9jf6 **(B)** Experimental workflow for VLPs production and purification. HeLa cells were transfected with the VLPs plasmid for 48 h. Culture supernatants were collected and subjected to differential centrifugation (300 × *g*, 2,000 × *g*, and 10,000 × *g*) to remove cells and debris. VLPs were then concentrated by ultracentrifugation (100,000 × *g*) through a 20% sucrose cushion for western blot analysis. Created with BioRender.com. https://BioRender.com/3pit7so **(C–L)** Impact of Rab10 depletion on SADS-CoV and SARS-CoV-2 VLPs secretion. HeLa WT and *Rab10*^*-/-*^ KO1 cells were transfected with plasmids producing SADS-CoV VLPs (C) or SARS-CoV-2 VLPs (H). Western blot was performed to analyze the expression of viral N protein, Rab10, and specific organelle markers: LMAN1 (ERGIC), TGN46 (*trans*-Golgi), and LAMP1 (lysosomes) in both cell lysates and purified VLPs. GAPDH was used as the loading control for cell lysates. Quantitative densitometric analysis of SADS-CoV N (D), LMAN1 (E), TGN46 (F), and LAMP1 (G) expression in cell lysates transfected with SADS-CoV VLPs and their respective secretion levels in the medium. Data compare HeLa WT (white bars) and *Rab10*^*-/-*^ KO1 (red bars). Quantitative densitometric analysis of SARS-CoV-2 N (I), LMAN1 (J), TGN46 (K), and LAMP1 (L) expression in cell lysates transfected with SARS-CoV-2 VLPs and their respective secretion levels in the medium. Data compare HeLa WT (white bars) and *Rab10*^*-/-*^ KO1 (red bars). Data points represent individual biological replicates (n = 3). Data are presented as means ± SD from three independent experiments. Statistical significance was analyzed by unpaired t-test. ^*^P < 0.05, ^**^P < 0.01, ^***^P < 0.001, ^****^P < 0.0001, ns, not significant.

Given that coronavirus release involves complex endomembrane trafficking, we utilized a replication-incompetent virus-like particles (VLPs) system to further delineate the specific role of Rab10 during the assembly and release stages. In studies of coronavirus egress, VLPs serve as vital tools as they are genome-less, enveloped particles that mimic wild-type virus behavior without being replication-competent [27]. Although the relative importance of each structural protein for VLPs formation varies across SARS-CoV-2 studies, E and M proteins are generally considered the primary drivers of SARS-CoV-2 VLPs assembly and release [28]. We employed an improved VLPs assembly plasmid to simulate the assembly and release of coronavirus particles [29]. This plasmid encodes the N, M, and E protein sequences linked by T2A self-cleaving peptides and an IRES bicistronic element downstream of a CMV promoter, allowing a single plasmid to produce VLPs containing all three structural proteins (Fig 7A).

After transfecting HeLa WT and *Rab10*^-/-^ KO1 cells with the VLPs plasmids for 48 h, we purified the SADS-CoV or SARS-CoV-2 VLPs via ultracentrifugation through a 20% sucrose cushion for western blot analysis (Fig 7B). Western blot analysis revealed that SADS-CoV N protein was clearly detectable in the supernatant of HeLa WT cells (Fig 7C). However, in *Rab10*^*-/-*^ KO1 cells, despite intracellular N protein expression levels showing no significant difference compared to WT cells, the level of N protein released into the supernatant was significantly reduced. Specifically, Rab10 deficiency led to an average reduction of 75% in SADS-CoV N protein secretion (Fig 7D). This phenomenon was highly consistent in the SARS-CoV-2 VLPs model, where N protein secretion decreased by an average of 90% (Fig 7H–I). These results strongly demonstrate that Rab10 exerts a critical proviral function during the particle egress of multiple coronaviruses, acting specifically at the assembly or release stages following viral protein synthesis.

Furthermore, we observed that the Rab10 protein itself is co-secreted into the extracellular space alongside the VLPs (Fig 7C, 7H). To further clarify the trafficking pathways involved, we monitored the release of major organelle markers during the coronavirus assembly and release process. The results showed that Rab10 depletion significantly inhibited the secretion levels of the *trans*-Golgi marker TGN46 and the lysosomal marker LAMP1 into the supernatant (Fig 7F–G, 7K–L), while the secretion of the ERGIC marker LMAN1 was not significantly affected (Fig 7E, 7J). This suggests that Rab10 likely serves as a component of the vesicular, mediating coronavirus release through either the classical post-Golgi secretory pathway or the lysosomal exocytosis pathway. In summary, using the VLPs model, we confirmed that Rab10 is a host factor essential for the efficient assembly or egress of SADS-CoV and SARS-CoV-2 virions. Additionally, Rab10 is a key regulator of the transport toward the TGN and lysosomal associated vesicular pathways after the virus leaves the ERGIC.

### Rab10 interacts specifically with the SADS-CoV E protein but is dispensable for M-E mediated viral assembly

**Fig 8:**
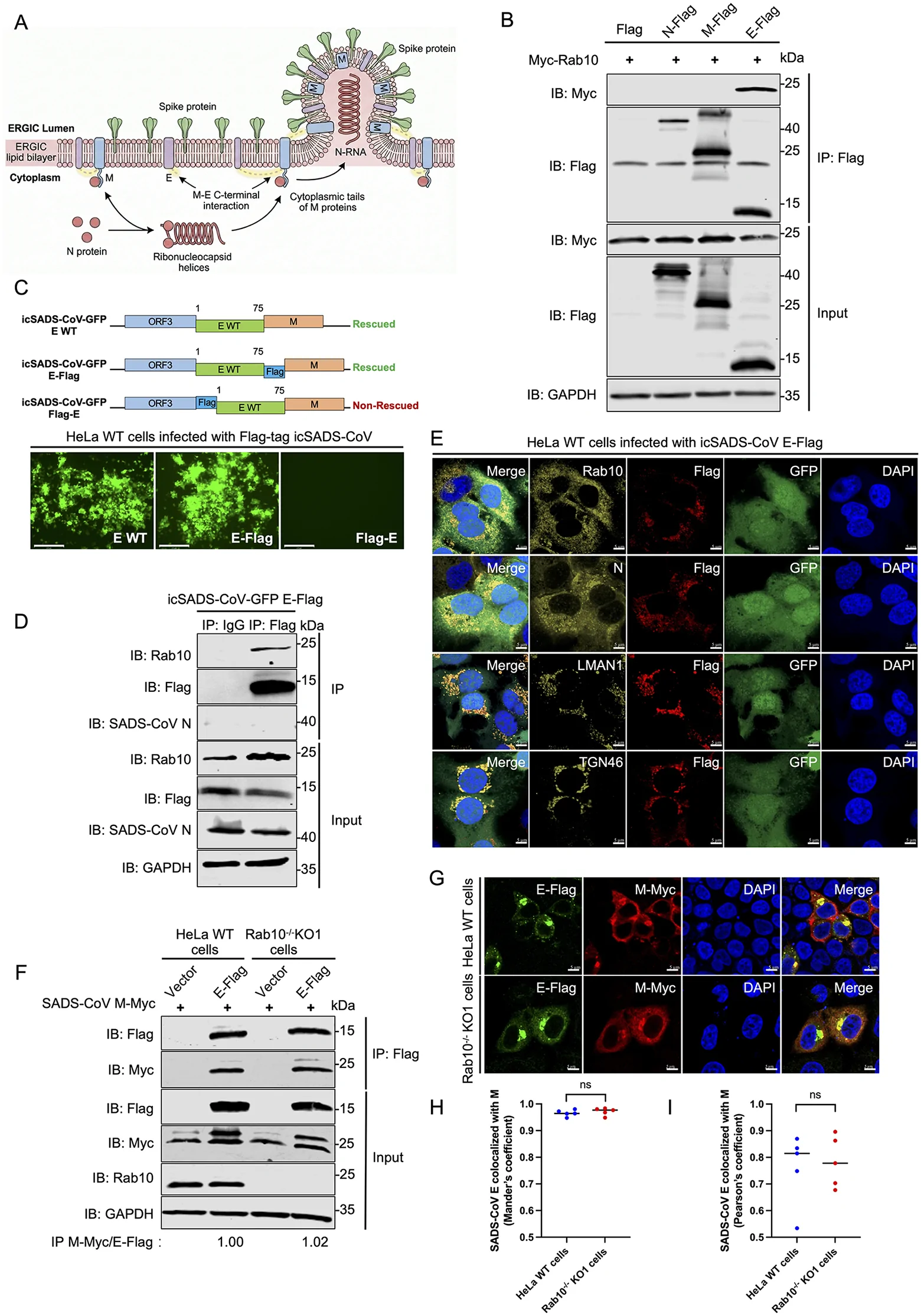
Rab10 interacts with SADS-CoV E protein but is dispensable for M-E interaction. **(A)** Schematic representation of SADS-CoV virion structure and its assembly process at the ERGIC. Viral structural proteins, including Spike (S), Envelope (E), Membrane (M), and Nucleocapsid (N), are shown alongside the viral RNA and nucleocapsid helices. The C-terminal interaction between M and E proteins is highlighted. Created with BioRender.com. https://BioRender.com/wl71gh9 **(B)** Co-IP analysis of the interaction between Rab10 and viral proteins. HeLa cells were co-transfected with Myc-Rab10 and Flag-tagged N, M, or E proteins. Cell lysates were immunoprecipitated with anti-Flag beads and analyzed by western blot with indicated antibodies. **(C)** Construction and rescue of recombinant SADS-CoV expressing tagged E proteins. The schematic shows the organization of icSADS-CoV-GFP containing E-WT, E-Flag, or Flag-E. Fluorescence images depict the successful rescue of E-WT and E-Flag viruses at Passage 2. Scale bar, 275 μm. **(D)** Co-IP analysis of endogenous Rab10 and viral E protein interaction in infected cells. HeLa WT cells were infected with icSADS-CoV-GFP E-Flag and subjected to IP using anti-Flag or control IgG beads. **(E)** Confocal microscopy analysis of icSADS-CoV-GFP E-Flag infected HeLa cells. Cells were stained for Rab10, SADS-CoV N, LMAN1 or TGN46 (yellow), Flag (red), and DAPI (blue). Infected cells are identified by GFP expression (green). Scale bar, 5 μm. **(F)** Co-IP analysis of interactions between SADS-CoV structural proteins in the presence or absence of Rab10. HeLa WT and *Rab10*^*-/-*^ KO1 cells were co-transfected with M-Myc and E-Flag. Cell lysates were immunoprecipitated with anti-Myc antibodies as indicated, and immunoblotted to detect interacting partners. The numbers below the blots indicate the relative intensity of the co-immunoprecipitated protein bands compared to the WT control (set as 1.00). **(G)** Representative confocal microscopy images showing the co-localization of E-Flag (green) and M-Myc (red) in HeLa WT and *Rab10*^*-/-*^ KO1 cells. Nuclei were stained with DAPI (blue). Scale bars represent 5 μm. **(H–I)** Statistical quantification of the co-localization between SADS-CoV E and SADS-CoV M. Mander's (H) and Pearson's (I) correlation coefficients were calculated to compare HeLa WT and *Rab10*^*-/-*^ KO1 cells. Data are presented as means ± SD from three independent experiments. Statistical significance was analyzed by unpaired t-test. ^*^P < 0.05, ^**^P < 0.01, ^***^P < 0.001, ^****^P < 0.0001, ns, not significant.

Coronavirus assembly is an orchestrated process requiring precise coordination between structural proteins, primarily driven by specific protein-protein interactions [30]. The M protein initiates virion assembly by recruiting the viral genome to budding sites through interactions with the C-terminus of the N protein. Simultaneously, M protein homodimerization and its interaction with the E protein drive membrane curvature and envelope formation, processes essential for viral budding and egress (Fig 8A).

To identify the specific viral component interacting with Rab10, we co-transfected HeLa cells with Myc-tagged Rab10 and Flag-tagged SADS-CoV N, M, or E proteins. Co-immunoprecipitation (Co-IP) analysis revealed a specific interaction between Rab10 and the E protein, whereas no significant binding was detected with the N or M proteins (Fig 8B). To validate these interactions in a physiologically relevant context, we employed a reverse genetics system to construct recombinant viruses expressing Flag-tagged E protein. We designed infectious clones of icSADS-CoV-GFP with the Flag sequence inserted at either the N- or C-terminus of the E gene to track its expression and localization. We found that only the C-terminally tagged clone (icSADS-CoV-GFP E-Flag) was successfully rescued and capable of stable passage, while the N-terminally tagged clone (icSADS-CoV-GFP Flag-E) was unable to rescued (Fig 8C). This suggests that the N-terminus of the E protein is critical for membrane topology, ion channel activity, or early recognition of other structural proteins, and is thus intolerant to tagging. In icSADS-CoV-GFP E-Flag infected cells, endogenous Co-IP confirmed the physical association between Rab10 and E during active infection (Fig 8D). Confocal microscopy further showed that E-Flag-positive intracellular structures were spatially associated with Rab10 and the viral N protein. E-Flag positive signals were also detected in association with the LMAN1 and TGN46, consistent with the localization of the E protein within virus assembly and trafficking related membrane compartments (Fig 8E). These observations suggest that the E protein may contribute to the recruitment of Rab10 to SADS-CoV associated vesicular structures.

Since the M-E interaction is the core driver of virion morphogenesis, we next investigated whether Rab10 regulates the interaction between the E and M proteins. We co-expressed E-Flag and M-Myc in both HeLa WT and *Rab10*^-/-^ KO1 cells. Co-IP assays demonstrated that the binding efficiency between M and E remained unchanged in the absence of Rab10, with normalized interaction ratios of 1.00 and 1.02, respectively (Fig 8F). Confocal microscopy further showed that E and M proteins co-aggregated in dense perinuclear regions (putative ERGIC sites) regardless of Rab10 status. The colocalization pattern of M and E at these assembly sites remained unaffected in *Rab10*^-/-^ KO1 cells (Fig 8G). Quantitative analysis using Mander's overlap coefficient and Pearson's correlation coefficient revealed no significant difference between the two cell types (Fig 8H–I). Taken together, these results demonstrate that while the SADS-CoV E protein specifically recruits Rab10 during infection, Rab10 does not directly participate in the M-E interaction that drives spatial viral assembly. This implies that Rab10 recruitment likely facilitates the subsequent viral release phase rather than the initial structural assembly of the virion.

### Rab10 mediated localization of the SADS-CoV E protein to the ERGIC and vesicular compartments

The ERGIC is a distinct membrane compartment that mediates cargo transport between the ER and the Golgi apparatus [31,32]. Coronaviruses assemble and bud at the ERGIC before exploiting host secretory pathways for subsequent transport and release, potentially through TGN or lysosome associated route [25].

**Fig 9:**
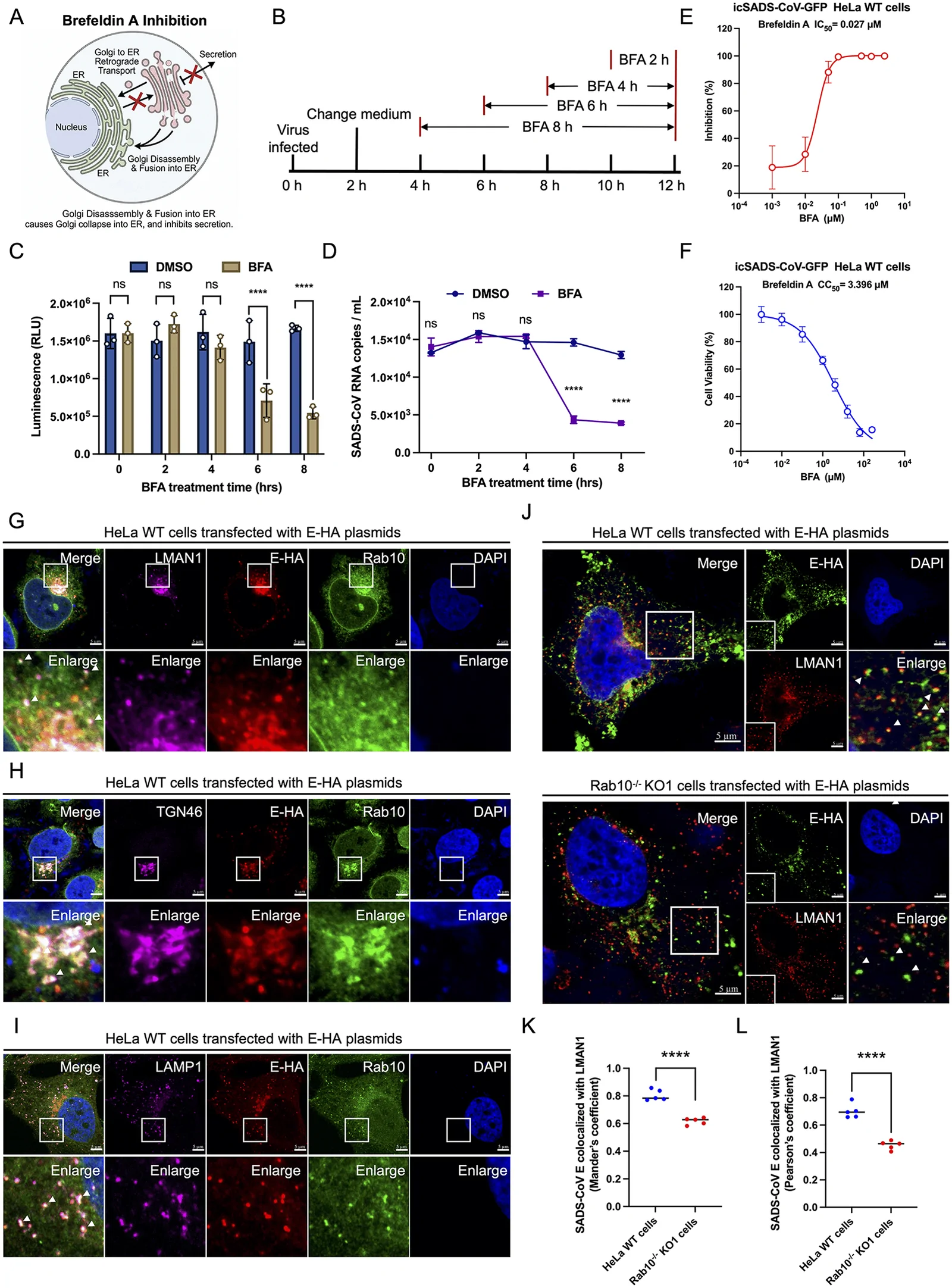
SADS-CoV utilizes the conventional secretory pathway, and Rab10 is required for the proper localization of SADS-CoV E protein to the ERGIC transport vesicles. **(A)** Schematic representation of BFA mechanism of action. BFA induces Golgi disassembly and fusion into the ER, thereby blocking the conventional protein secretory pathway. Created with BioRender.com. https://BioRender.com/xbn3zy9 **(B)** Experimental timeline for BFA treatment. HeLa cells were infected with SADS-CoV, and BFA was added at the indicated time points (2, 4, 6, 8 h) before samples were harvested at 12 h. **(C–D)** Effect of BFA treatment on viral secretion and replication. Gluc secretion (C) and extracellular viral RNA copies (D) were measured at the indicated time points. **(E–F)** Dose-response curve showing the inhibition of icSADS-CoV-GFP replication and cytotoxicity in HeLa cells treated with increasing concentrations of BFA. The IC_50_ (E) and CC_50_ (F) were determined in HeLa cells using inhibition assays and CCK-8 tests, respectively. **(G–I)** Representative confocal images showing the co-localization of transiently expressed E-HA (red) with endogenous Rab10 (green) and organelle markers LMAN1 (G), TGN46 (H) or LAMP1 (I) (purple) in HeLa WT cells. Nuclei were stained with DAPI (blue). White arrowheads indicate the punctate distribution of E protein at the ERGIC. Scale bar, 5 μm. **(J)** Localization of SADS-CoV E protein in the absence of Rab10. Representative confocal images showing the distribution of E-HA (green) relative to LMAN1 (red) in HeLa WT and *Rab10*^*-/-*^ KO1 cells. White arrowheads indicate the punctate distribution of E protein at the ERGIC. Scale bar, 5 μm. **(K–L)** Statistical quantification of the co-localization between SADS-CoV E and LMAN1. Mander's (K) and Pearson's (L) correlation coefficients were calculated to compare HeLa WT and *Rab10*^*-/-*^ KO1 cells. Data are presented as means ± SD from three independent experiments. Statistical significance was analyzed by unpaired t-test. ^*^P < 0.05, ^**^P < 0.01, ^***^P < 0.001, ^****^P < 0.0001, ns, not significant.

We first investigated whether the ER-to-Golgi trafficking pathway is essential for SADS-CoV infection using Brefeldin A (BFA), which disrupts Golgi and block ER-to-Golgi transport (Fig 9A). Following icSADS-CoV-GFP infection, BFA was added at different time points, and all samples were collected at a common endpoint (Fig 9B). Secreted Gaussia luciferase (Gluc) was used to verify inhibition of the biosynthetic secretory pathway. BFA significantly reduced both Gaussia luciferase secretion and extracellular viral RNA after 6–8 h of treatment (Fig 9C–D). Furthermore, BFA displayed potent antiviral activity with an IC_50_ of 0.027 μM and a CC_50_ of 3.396 μM, corresponding to a selectivity index of 126 (Fig 9E–F). These results indicates that the ER-to-Golgi trafficking is required for efficient SADS-CoV propagation. However, these findings do not specifically establish the involvement of downstream lysosomal transport.

We therefore evaluated the contribution of late endosomal/lysosomal trafficking. siRNA-mediated depletion of Arl8b, Rab7, or VPS39, confirmed by RT-qPCR, markedly reduced the GFP-positive infection signal and intracellular SADS-CoV RNA levels in wild-type and *Rab10*^-/-^ KO1 cells (S3A–G Fig.), indicating that these lysosomal trafficking regulators contribute to efficient SADS-CoV infection and dissemination. We further used CID-1067700 to interfere with lysosomal trafficking. Treatment with CID-1067700 markedly reduced the icSADS-CoV-GFP GFP-positive infection signal, similar to BFA treatment (S3H Fig.). To distinguish effects on viral release from those on viral entry or genome replication, we analyzed the release of SADS-CoV VLPs. CID-1067700 substantially reduced the amount of N protein detected in the culture medium, whereas intracellular N-protein abundance remained largely unchanged. The medium-to-cell lysate N-protein ratio decreased from 1.00 in DMSO-treated cells to 0.41 following CID-1067700 treatment, compared with 0.12 after BFA treatment (S3I Fig.). Consistently, time-of-addition experiments showed that prolonged treatment with either BFA or CID-1067700 reduced extracellular SADS-CoV RNA, with significant inhibition after 6–8 h of treatment (S3J–K Fig.). Together, these results support the involvement of late endosomal/lysosomal trafficking in SADS-CoV dissemination and VLP release.

To further determine whether Rab10 participates in the intracellular trafficking of the SADS-CoV E protein, HeLa cells were transfected with an E-HA expression plasmid and examined by confocal microscopy. Concurrently, E-HA expression induced the fragmentation of the compact ERGIC structure into numerous vesicular signals, within which Rab10 colocalized with the E-HA (Fig 9G), consistent with the localization observed in cells ectopically expressing HA-tagged E protein, where E-HA was also associated with Rab10-positive compartments containing the TGN marker TGN46 or the lysosomal marker LAMP1 (Fig 9H–I). Enlarged images revealed multiple overlapping punctate signals among E-HA, Rab10, and the respective organelle markers. These observations suggest that the SADS-CoV E protein is associated with Rab10-positive membrane compartments related to the ERGIC, TGN, and lysosomes.

Previous studies have suggested that the coronavirus E protein can be released in membrane associated vesicular structures, potentially through their membrane remodeling or ion channel [33]. We therefore compare the association of E-HA with LMAN1 in wild-type and *Rab10*^-/-^ KO1 cells. Interestingly, in Rab10-knockout cells, while the E protein still induced the formation of dispersed vesicles, it significantly failed to load onto LMAN1-positive ERGIC vesicles (Fig 9J). Quantitative analysis confirmed that both Mander's and Pearson's correlation coefficients for E protein colocalization with LMAN1 were significantly reduced in Rab10-deficient cells (Fig 9K–L). These findings suggest that Rab10 facilitates the proper association and trafficking of E protein within ERGIC vesicles for transport.

In summary, our results demonstrate that SADS-CoV infection depends on proper trafficking through the ER-to-Golgi/lysosome secretory pathway, with the E protein playing a central role in hijacking this host pathway. Furthermore, Rab10 acts as a critical guide that facilitates the trafficking of SADS-CoV associated vesicular structures through ERGIC-TGN/lysosomal related compartments, thereby promoting efficient viral dissemination and particle release.

### The C-terminal 64–75 aa region of SADS-CoV E protein is essential for Rab10 recruitment and ERGIC-mediated trafficking

**Fig 10:**
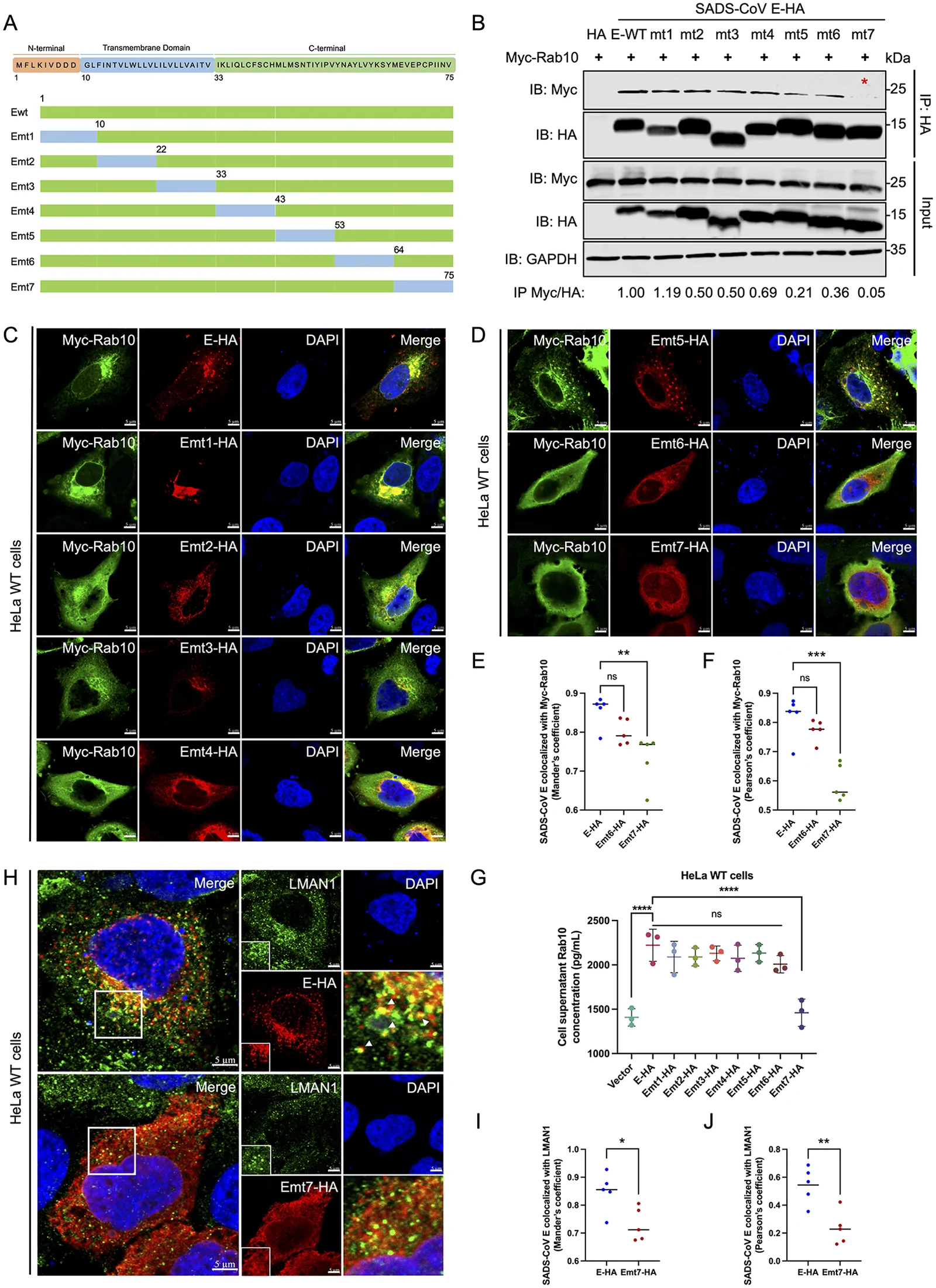
The C-terminal region of SADS-CoV E protein is essential for its interaction with Rab10 and its localization to the ERGIC. **(A)** Schematic diagram of SADS-CoV E protein truncation mutants. The E protein consists of an N-terminal domain (residues 1–10), a transmembrane domain (residues 10–33), and a C-terminal domain (residues 33–75). Truncation mutants (mt1–mt7) were constructed by sequentially deleting residues from the C-terminus. **(B)** Co-IP analysis of the interaction between Rab10 and E protein mutants. HeLa cells were co-transfected with Myc-Rab10 and HA-tagged E-WT or mutants (mt1–mt7). Cell lysates were immunoprecipitated with anti-HA beads and analyzed by western blot with anti-Myc and anti-HA antibodies. GAPDH served as the loading control for input samples. The IP Myc/HA ratios, normalized to E-WT, are indicated below the blots. **(C–D)** Confocal microscopy analysis of the co-localization between Myc-Rab10 (green) and HA-tagged E protein mutants (red) in HeLa WT cells. Nuclei were stained with DAPI (blue). Scale bar, 5 μm. **(E–F)** Statistical quantification of co-localization using Mander's and Pearson's correlation coefficients. Panels (E) and (F) show the co-localization analysis between SADS-CoV E (WT, mt6, or mt7). **(G)** Measurement of Rab10 concentration in the cell culture supernatant. HeLa cells were transfected with HA-tagged E protein mutants, and the concentration of secreted Rab10 was quantified by ELISA. **(H)** Representative confocal images showing the co-localization of E-WT-HA or Emt7-HA (red) with the ERGIC marker LMAN1 (green) in HeLa WT cells. Nuclei were stained with DAPI (blue). Scale bar, 5 μm. **(I–J)** Statistical quantification of co-localization using Mander's and Pearson's correlation coefficients. Panels (I) and (J) show the co-localization analysis between E-HA (WT or mt7) and LMAN1. Data are presented as means ± SD from three independent experiments. E–F, G, one-way ANOVA with Dunnett's test. I–J, unpaired t-test. ^*^P < 0.05, ^**^P < 0.01, ^***^P < 0.001, ^****^P < 0.0001, ns, not significant.

To further delineate the specific molecular motifs within the SADS-CoV E protein responsible for Rab10 recruitment, we designed and constructed seven alanine-substitution mutants (Emt1–Emt7) covering the full-length protein based on its structural domains (N-terminus, transmembrane domain, and C-terminus) (Fig 10A). Co-IP assays revealed a robust interaction between the wild-type E protein (E-WT) and Myc-tagged Rab10. Conversely, the Emt7 mutant, featuring mutations in residues 64–75 of the C-terminus, almost entirely abolished Rab10 binding (relative ratio = 0.05). Additionally, Emt6 (residues 53–64) exhibited substantially weakened binding affinity (ratio = 0.36) (Fig 10B).

We next examined the intracellular spatial distribution of these mutants and Rab10 using confocal microscopy. While E-WT and the majority of mutants exhibited significant colocalization with Rab10, the spatial overlap between the Emt7 mutant and Rab10 was markedly reduced (Fig 10C–D). Quantitative analysis further confirmed that both Mander's and Pearson's correlation coefficients for Emt7 were significantly lower than those for E-WT and Emt6 (Fig 10E–F). In addition, Emt7 induced substantially less extracellular Rab10 detection than E-WT, suggesting that the extracellular association of Rab10 observed following E expression depends, at least in part, on the E–Rab10 interaction (Fig 10G).

We next examined whether the loss of Rab10 binding affected the subcellular position of the E protein relative to LMAN1-labeled vesicles. In HeLa WT cells, E-WT displayed a characteristic punctate distribution and localized to LMAN1 positive ERGIC-derived vesicles. In contrast, the Emt7 mutant exhibited a distinct diffuse cytoplasmic distribution and a significantly reduced overlap with LMAN1 labeled compartments (Fig 10H). Quantitative analysis showed a significant decrease in both Pearson's and Mander's coefficients for Emt7 compared to E-WT (Fig 10I–J). These findings suggest that the C-terminal Rab10-binding region contributes to the proper localization and trafficking of E within ERGIC-associated membrane compartments.

Finally, we utilized a reverse genetics system to evaluate the importance of this C-terminal region for viral fitness. Premature stop codons were introduced into the icSADS-CoV-GFP backbone to generate various C-terminal truncations (S4A Fig). Our results showed that the icSADS-CoV-GFP E Δ73–75aa, Δ69–75aa, and Δ65–75aa mutants could not be rescued at passage 1. In contrast, the wild-type virus was successfully recovered and exhibited robust fluorescence signaling (S4B Fig). This indicates that subcellular mislocalization resulting from C-terminal deletions is a key factor precluding viral rescue. Taken together, these data strongly demonstrate that the 64–75 aa region of the SADS-CoV E protein is a critical molecular determinant for recruiting Rab10, ensuring correct secretion/trafficking from the ERGIC, and maintaining the viral life cycle.

### Disruption of the Rab10 binding site in the SADS-CoV E protein compromises the release process of progeny virions

**Fig 11:**
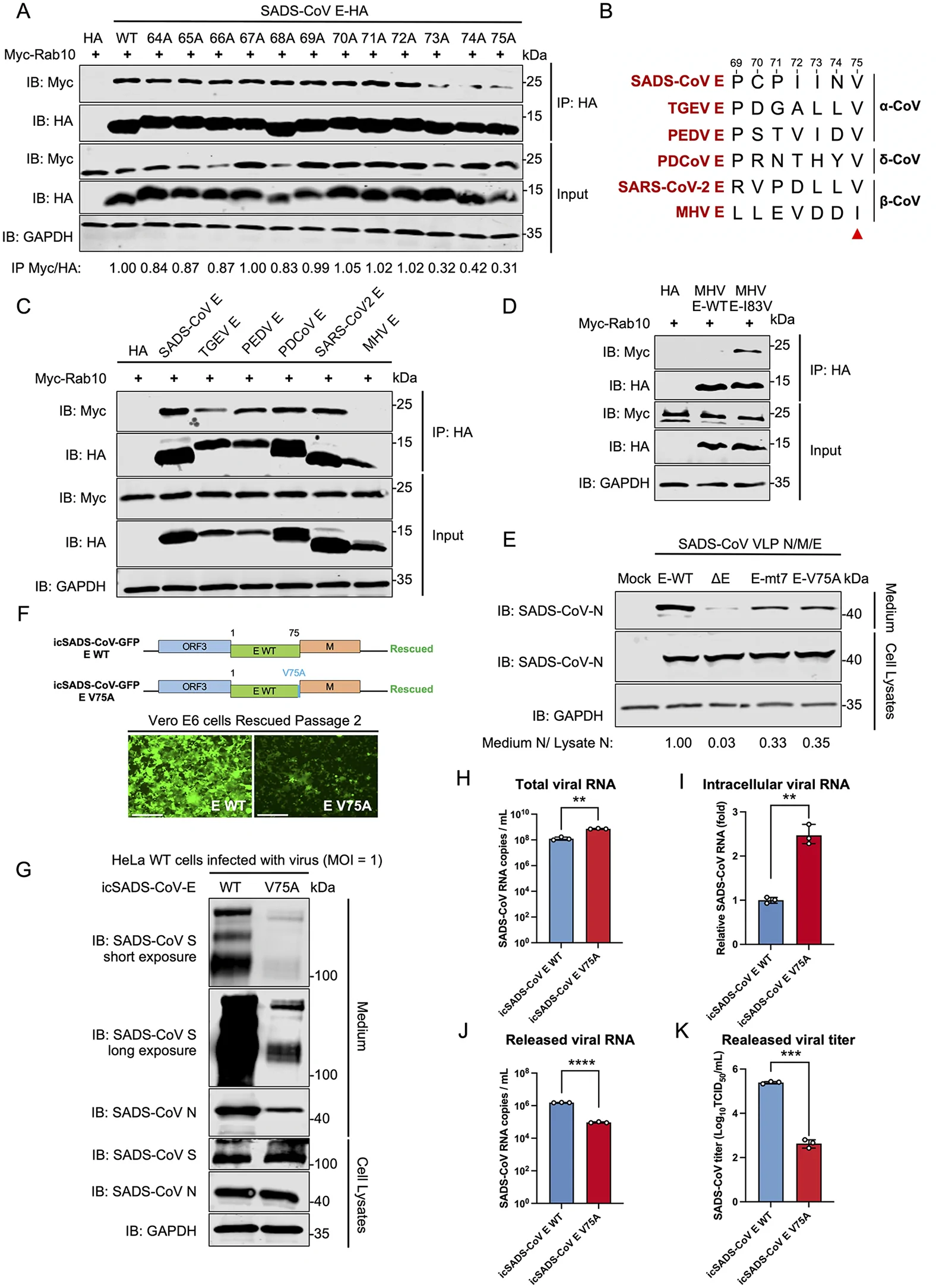
The C-terminal V75 residue of the SADS-CoV E protein is critical for Rab10 interaction and viral egress. **(A)** Alanine scanning mutagenesis of the C-terminal residues (64–75) of SADS-CoV E protein. HeLa cells were co-transfected with Myc-Rab10 and HA-tagged E-WT or alanine mutants. Cell lysates were immunoprecipitated with anti-HA beads and analyzed by western blot. **(B)** Sequence alignment of the C-terminal tails of E proteins from diverse coronaviruses, including alphacoronaviruses (SADS-CoV, TGEV, PEDV), deltacoronaviruses (PDCoV), and betacoronaviruses (SARS-CoV-2, MHV). **(C)** Co-IP analysis of Rab10 with E proteins from the indicated coronaviruses. HeLa cells were co-transfected with Myc-Rab10 and HA-tagged E from the indicated coronaviruses. Cell lysates were immunoprecipitated with anti-HA beads and analyzed by western blot. **(D)** Rescue of Rab10 interaction with MHV E protein. HeLa cells were co-transfected with Myc-Rab10 and HA-tagged MHV E-WT or the I83V mutant. **(E)** SADS-CoV VLPs secretion assay. HeLa cells were transfected with VLPs plasmids (N/M/E) containing E-WT, ΔE, E-mt7, or E-V75A. Cell lysates and concentrated medium samples were analyzed by western blot for SADS-CoV N. The ratio of N in medium versus lysate is shown below. **(F)** Schematic representation and rescue of recombinant icSADS-CoV-GFP E-WT and E-V75A. Fluorescence images show the successful rescue and propagation of viruses at Passage 2 in Vero E6 cells. Scale bar, 275 μm. **(G)** Comparison of viral protein expression and release. HeLa cells were infected with icSADS-CoV-GFP E-WT or E-V75A (MOI = 1) for 16 h. Cell lysates and concentrated culture supernatants (Medium) were analyzed by western blot for SADS-CoV S and N proteins. **(H–K)** Quantitative analysis of SADS-CoV egress for E-WT and E-V75A viruses. Metrics include total viral RNA (H), relative intracellular mRNA (I), released viral RNA in the supernatant (J), and released infectious viral titers measured by TCID_50_ (K). Data points represent individual biological replicates (n = 3). Data are presented as means ± SD from three independent experiments. Statistical significance was analyzed by unpaired t-test. ^*^P < 0.05, ^**^P < 0.01, ^***^P < 0.001, ^****^P < 0.0001, ns, not significant.

To precisely map the functional motif within the SADS-CoV E protein responsible for Rab10 recruitment, we performed alanine-scanning mutagenesis across residues 64–75. Co-IP assays revealed that the C-terminal residues I73, N74, and V75 are critical determinants, as their substitution with alanine significantly abrogated the interaction with Rab10 (Fig 11A). This region corresponds to a putative PDZ-binding motif, which typically mediates specific interactions with host PDZ-domain proteins to target viral proteins to the ERGIC assembly sites. Sequence alignment across diverse coronaviruses showed that while residues I73 and N74 are poorly conserved, V75 is nearly universal among alpha- and delta-coronaviruses, with the exception of MHV, which possesses an isoleucine at the corresponding position (Fig 11B).

We further examined the interaction between Rab10 and E proteins from TGEV, PEDV, PDCoV, SARS-CoV-2, and MHV. Co-IP results demonstrated that the E proteins of TGEV, PEDV, PDCoV, and SARS-CoV-2 all interact with Rab10, whereas MHV E fails to do so (Fig 11C). To confirm the role of the terminal valine in this interaction, we introduced an I83V mutation into the MHV E C-terminus. This single substitution was sufficient to enhance its binding affinity for Rab10 (Fig 11D), identifying the C-terminal valine as a key molecular determinant for Rab10 engagement across multiple coronavirus genera.

The functional consequences of these mutations were first evaluated using the VLPs secretion system. Compared to the wild-type (E-WT), both the Emt7 (residues 64–75) and E-75A mutants markedly reduced the amount of the secretion of N protein into the culture supernatant, indicating impaired VLPs egress (Fig 11E). To validate these phenotypes during authentic infection, we generated a recombinant SADS-CoV mutant carrying the V75A substitution, designated icSADS-CoV-GFP E V75A using reverse genetics (Fig 11F). Although the V75A mutant remained viable and could be rescued, it exhibited a pronounced release defect in HeLa cells. Western blot analysis of infected HeLa WT cells showed that while intracellular levels of N and S proteins were comparable to or even higher than those of WT, their accumulation in the culture supernatant was markedly reduced (Fig 11G). Consistently, the V75A mutant showed slightly increased intracellular viral mRNA and approximately 2.3-fold higher intracellular viral RNA levels, but extracellular viral RNA and progeny infectious titers were reduced by approximately 10-fold and 100-fold, respectively (Fig. 11H–K).

**Fig 12:**
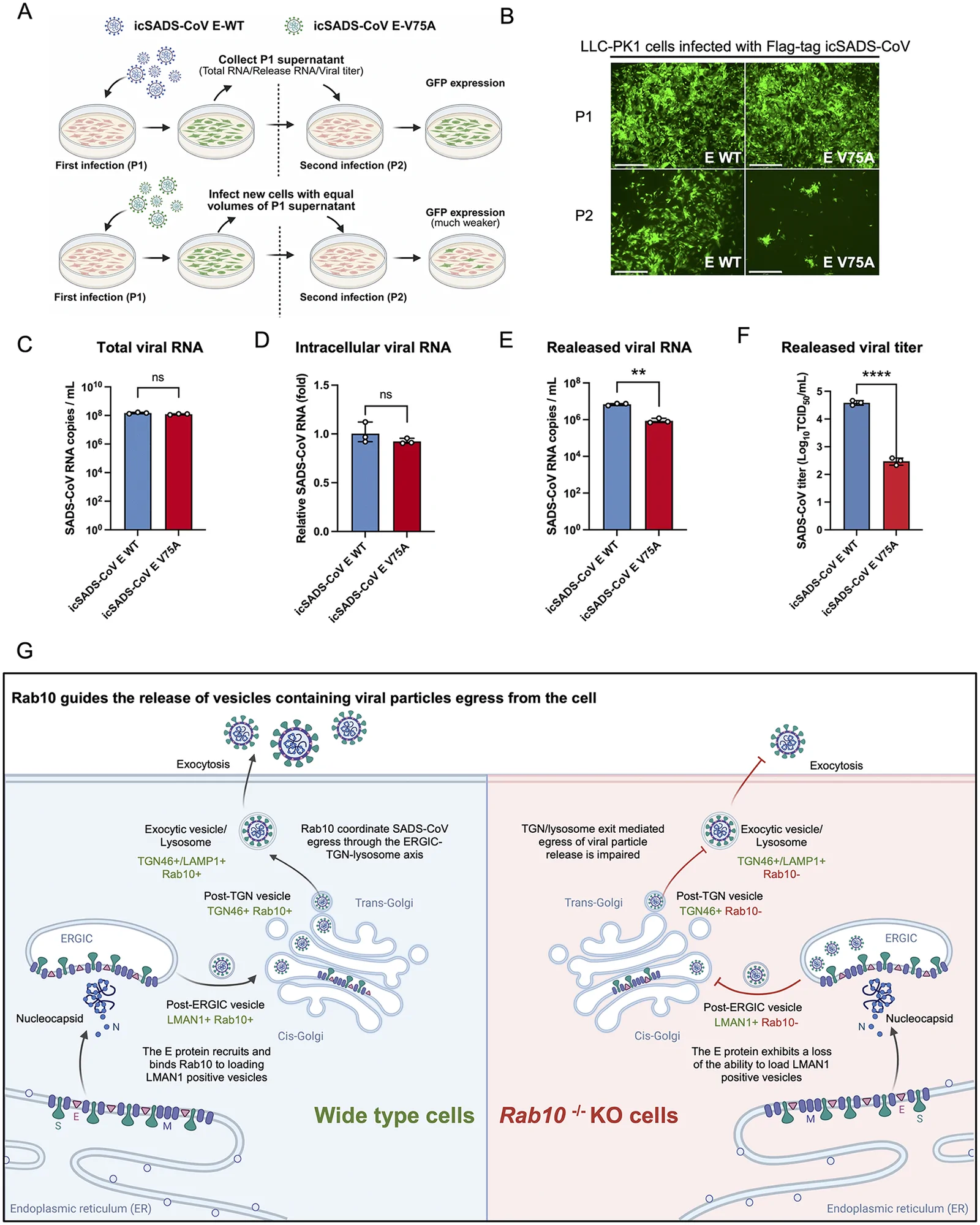
The V75 residue of the SADS-CoV E protein is also required for viral release in LLC-PK1 cells and proposed model for Rab10-mediated SADS-CoV egress via the ERGIC-TGN-lysosome axis. **(A)** Schematic representation of the two-round infection assay used to compare recombinant SADS-CoV carrying the wild-type E protein (icSADS-CoV-GFP E-WT) with the E-V75A mutant (icSADS-CoV-GFP E-V75A). Cells were initially infected with the indicated viruses during the first round of infection (P1). P1 supernatants were collected for the measurement of viral RNA and infectious virus titers when GFP fluorescence becomes uniformly bright and comparable between the two groups. Equal volumes of P1 supernatants were subsequently used to infect cells during the second round of infection (P2), and infection was evaluated by GFP expression. Created with BioRender.com. https://BioRender.com/voc0v0c **(B)** Representative fluorescence images showing GFP expression in cells infected with icSADS-CoV-GFP E-WT or icSADS-CoV-GFP E-V75A during P1 and P2. Comparable GFP signals were observed for the two viruses during P1, whereas GFP expression was markedly reduced in cells infected with the E-V75A mutant during P2. Scale bar, 275 μm. **(C–F)** Quantitative analysis of SADS-CoV egress for E-WT and E-V75A viruses. Metrics include total viral RNA (C), relative intracellular mRNA (D), released viral RNA in the supernatant (E), and released infectious viral titers measured by TCID_50_ (F). Data points represent individual biological replicates (n = 3). Data are presented as means ± SD from three independent experiments. Statistical significance was analyzed by unpaired t-test. ^*^P < 0.05, ^**^P < 0.01, ^***^P < 0.001, ^****^P < 0.0001, ns, not significant. **(G)** Proposed model for Rab10-mediated SADS-CoV egress. In wild-type cells, during a productive infection, the viral E protein recruits and binds Rab10, facilitating its loading onto LMAN1-positive vesicles at the ERGIC. Rab10 subsequently coordinates the trafficking of virion-containing vesicles through TGN46-positive post-TGN compartments and TGN46/LAMP1-positive exocytotic vesicle/lysosome compartments, ultimately facilitating virion release by exocytosis. In Rab10-knockout cells, the E protein exhibits a loss of ability to recruit Rab10, the loading of viral cargo into LMAN1-positive vesicles is disrupted. Consequently, the trafficking of post-TGN vesicles is stalled, and the exit of viral particles from the TGN/lysosome compartments is significantly impaired, leading to reduced viral egress from the host cell. Created with BioRender.com. https://biorender.com/ovfgvpj.

We further examined the V75A phenotype in porcine LLC-PK1 cells to confirm its physiological relevance (Fig. 12A). Cells were initially infected with the indicated viruses during the first round of infection (P1). P1 supernatants were collected for the measurement of viral RNA and infectious virus titers when GFP fluorescence becomes uniformly bright and comparable between the two groups. However, when equal volumes of P1 culture supernatants were transferred to LLC-PK1 cells, the V75A mutant produced markedly weaker GFP signals during the second passage, indicating reduced production of extracellular infectious progeny (Fig. 12B). Total viral RNA yields and intracellular viral RNA levels were not significantly different between the wild-type and V75A viruses (Fig. 12C and D). In contrast, extracellular viral RNA was reduced by approximately one order of magnitude, and the released infectious titer decreased by approximately two orders of magnitude in the V75A mutant (Fig. 12E and F). These results confirm that the V75A substitution primarily impairs viral egress rather than affecting intracellular viral RNA accumulation, and that this phenotype is conserved in a porcine cell line.

In summary, these findings identify the C-terminal residues 73–75 (specifically V75) as a critical molecular determinant for Rab10 recruitment. Disruption of this motif leads to the intracellular sequestration of viral components and a concomitant failure in efficient virion exocytosis.

## Discussion

The precise orchestration of coronavirus assembly and egress involves a highly complex exploitation and remodeling of host membrane systems. While the mechanisms by which viruses hijack host machinery are increasingly understood, identifying the specific host factors indispensable for virion formation and export, as well as how viral structural proteins coordinate these late-stage processes, remains a critical area of investigation [34]. In this study, through a genome-wide CRISPR screen, we demonstrated for the first time that the small GTPase Rab10 as an important proviral host factor for SADS-CoV infection. Our findings reveal that Rab10 primarily facilitates a late stage of the viral life cycle by coordinating the trafficking and extracellular release of virus-containing compartments, rather than by directly regulating viral entry, genome replication, or the initial association of viral structural proteins. SADS-CoV hijacks Rab10 to drive the precise loading of the viral E protein into transport vesicles derived from the ERGIC, ensuring the subsequent trafficking of infectious progeny virions. Subsequently, SADS-CoV exploits a Rab10 dependent vesicular trafficking pathway associated with the ERGIC, TGN, and late endosomal/lysosomal compartments to facilitate non-lytic egress (Fig 12G). By mapping recruitment signals to a specific C-terminal PDZ-binding motif on the E protein, we show that the terminal residue V75 is responsible for directly recruiting Rab10. This mechanism appears conserved across several alpha- and beta-coronaviruses. These results suggest that the ER-to-Golgi pathway and the E-Rab10 interaction interface represent promising therapeutic targets for treating diseases caused by coronaviruses and other viruses utilizing similar trafficking routes.

Rab GTPases constitute the largest family of small GTPases involved in vesicle trafficking, with nearly 70 members localizing to specific membrane compartments to regulate the transport of cellular cargo between organelles [35]. Enveloped viruses exploit Rab proteins at multiple stages of infection, including endocytosis-mediated entry through Rab5 positive early endosomes or Rab7/Rab9 positive late endosomes, as reported for PHEV [36], PEDV [37], and PDCoV [38]; late-stage assembly and release involving Rab6 [39], Rab8 [40], or Rab27a [41]; and the transport of envelope glycoproteins following post-translational modification [42]. Rab10 exhibits diverse subcellular localizations and regulates trafficking from the TGN and endosomes compartments to the plasma membrane, thereby contributing to cell polarity and organelle homeostasis [21]. Functional analyses further indicated that Rab10 acts predominantly during viral trafficking and release. Rab10 knockout significantly reduced the release of infectious virions and viral proteins, a finding validated in both authentic virus infection and VLPs models. In contrast, quantitative colocalization analyses showed that Rab10 deficiency did not significantly alter the spatial association between the SADS-CoV E and M proteins, suggesting that the egress defect is unlikely to result from impaired E–M colocalization and instead occurs during a subsequent trafficking or release step. A significant finding of this research is the observation that Rab10 is co-detected with SADS-CoV particles proteins and vesicular markers in concentrated extracellular vesicle-enriched fractions. Time-course LDH-release assays and the absence of the intracellular marker Calnexin from these fractions argue against extensive cell lysis as the principal source of extracellular Rab10 at the time of sample collection. Sucrose density gradient centrifugation analysis revealed that, upon infection, the density profile of Rab10 aligns precisely with that of the SADS-CoV N protein. This evidence suggests that Rab10 may be a structural constituent of the transport vesicles that encapsulate mature virions and direct these virus-containing vesicles through the host secretory pathway toward the plasma membrane.

Coronaviruses are reported to undergo virion assembly and budding within the ERGIC. Following assembly, mature virions of multiple coronaviruses, including SARS-CoV-2, are generally thought to be transported from the ERGIC to the Golgi and released via conventional secretory pathways [10,43]. However, evidence also supports that certain beta-coronaviruses, such as SARS-CoV-2 [11] and MHV [13], exit cells through Golgi-independent lysosomal exocytosis or unconventional routes like exosomes [25]. In this study, In the present study, BFA treatment markedly reduced SADS-CoV propagation and VLP release, consistent with an important role for the ERGIC–Golgi secretory machinery. The TEM provided high-resolution insights into the morphogenesis of SADS-CoV. Assembled virions were observed within vesicular tubular clusters (VTCs), which are pre-Golgi intermediates. These particles were further organized into small vesicles containing single virions or fused into the characteristic LVCVs. Confocal microscopy showed spatial associations among SADS-CoV proteins, Rab10, and the compartment markers LMAN1, TGN46, and LAMP1. In Rab10 knockout cells, virus and LAMP1 positive structures accumulated in restricted intracellular regions rather than appearing as dispersed cytoplasmic puncta, suggesting defective trafficking or redistribution of virus-associated compartments. Furthermore, biochemical analysis of the extracellular space revealed that TGN46 and LAMP1 are secreted along with the viral particles. Moreover, inhibition lysosomal trafficking with CID-1067700 reduced extracellular viral RNA in a treatment dependent manner and selectively decreased extracellular N protein in the SADS-CoV VLPs system, with a comparatively smaller effect on intracellular N-protein abundance. Depletion of Arl8b, Rab7, or VPS39 also reduced SADS-CoV infection and viral RNA abundance. This suggests that SADS-CoV egress involves transport through the Rab10 associated ER-to-Golgi pathway, followed by accumulation at the TGN and subsequent release via the lysosomal pathway, rather than a simple classical secretory route or purely Golgi-independent exocytosis. This confirms that SADS-CoV utilizes host vesicle trafficking systems for non-lytic egress.

Recent studies have identified the interaction interface between coronavirus assembly/egress and host endomembrane systems as a novel target for antiviral strategies. For instance, the small molecule CIM-834 inhibits SARS-CoV-2 by stabilizing a specific M protein conformation to block assembly [44], while peptides disrupting the M-ARF1 interaction effectively halt viral trafficking [45]. Building on this paradigm, our study identifies the SADS-CoV E protein and its recruitment of Rab10 as a similarly vulnerable axis in the viral life cycle. We demonstrate that Rab10 is essential for loading the E protein into ERGIC-derived transport vesicles; its absence arrests the E protein-mediated transit of virions through the Golgi/lysosome pathway. Crucially, our mapping analysis pinpointed the C-terminal PDZ-binding motif of the E protein as the direct binding site for Rab10. The PDZ domains of coronavirus E proteins contribute to protein-interaction, intracellular trafficking, and viral pathogenesis [46,47]. Sequence analysis of E proteins from diverse coronaviruses revealed that the terminal valine residue in the PDZ-binding motif (V75 in SADS-CoV) is highly conserved. Interestingly, while the MHV E protein possesses an isoleucine (I83) at the corresponding position and does not naturally interact with Rab10, a single point mutation (MHV E I83V) is sufficient to enable its interaction with Rab10. These observations raise the possibility that related coronaviruses may exploit Rab10 or functionally analogous host trafficking factors through their E-protein C termini. Nevertheless, the extent to which this mechanism is conserved across coronavirus genera requires further investigation.

To confirm the functional significance of this residue, an infectious clone of SADS-CoV (icSADS-CoV-GFP) was used to generate a mutant virus with a V75A substitution in the E protein. The biological characterization of the icSADS-CoV-GFP E-V75A virus demonstrated that the V75A mutation severely impaired the release phase compared to the wild-type (E-WT) virus. This provides conclusive evidence that the terminal valine in the PDZ-binding motif is essential for the Rab10-E interaction and for efficient non-lytic viral egress. Consequently, the V75-centered PBM interface represents a high-value therapeutic pocket. Much like the strategies targeting the M protein, developing small-molecule inhibitors or peptidomimetics that mask this conserved valine residue could provide a potent, broad-spectrum approach to block the egress of SADS-CoV and related pathogenic coronaviruses.

In summary, this study identifies Rab10 as an important host regulator of SADS-CoV non-lytic egress and elucidates how the E protein engages host trafficking machinery to facilitate viral transport and release. This study has several limitations. First, the role of Rab10 during infection has not yet been validated *in vivo*, partly because systemic Rab10 knockout results in embryonic lethality and thereby complicating the development of systemic animal models. Second, the membrane topology, extracellular form, and precise membrane association of Rab10 remain unresolved. Future studies should further characterize the molecular interface between the E protein and Rab10, clarify the associated vesicular trafficking pathway, and evaluate competitive peptides or small molecules that interfere with the E–Rab10 interaction in appropriate animal models. Such studies will help determine the therapeutic potential of targeting this pathway for the control of SADS-CoV and related porcine coronaviruses.

## Materials and methods

### Generation of plasmid constructs

The following plasmids were constructed and used in this study: pCAGGS-hRab10-Myc for expressing human Rab10; a series of pCAGGS-based plasmids for expressing C-terminally HA-tagged E proteins from SADS-CoV (including mutants), PEDV, TGEV, PDCoV, SARS-CoV-2, and MHV (including I83V mutant), as well as C-terminal HA-tagged M and N proteins from SADS-CoV; pLV2-TRE3GS-Rab10-TetOne-Blast for doxycycline-inducible expression of Rab10; pcDNA3.1-based plasmids for co-expressing the N, M and E protein (separated by a T2A cleavage site) from either SARS-CoV-2 or SADS-CoV for VLPs production; and a set of pCAGGS plasmids for expressing Myc- or Flag-tagged SADS-CoV M, E, and N proteins. All constructs were verified by DNA sequencing.

### Cells and viruses

All cell lines, including HeLa (ATCC, CCL-2), Vero E6 (ATCC, CRL-1586), HEK 293T (ATCC, CRL-3216), BHK21 (ATCC, CCL-10), IPI-2I (Porcine Intestinal) and LLC-PK1 (Porcine kidney, ATCC, CL-101), were cultured in Dulbecco's Modified Eagle Medium (DMEM, Gibco, 11965092) supplemented with 10% fetal bovine serum (FBS, JinYuanKang, FBS-300) and 1% penicillin-streptomycin (Sigma, #P4333) at 37°C in a humidified incubator with 5% CO_2_. All cell lines tested negative for mycoplasma contamination. SADS-CoV (GenBank: MF094681), the icSADS-CoV-GFP (in which the GFP-T2A sequence is substituted at the 5’ end of the N gene) reporter virus, PEDV (GenBank: GU372744), TGEV (GenBank: EU074218) and PDCoV (GenBank: KU981059) were stored in our laboratory. The SADS-CoV, icSADS-CoV-GFP and PEDV viral titers were determined by measuring the TCID_50_ on Vero E6 cells, while TGEV and PDCoV were propagated and their titers were determined by TCID_50_ assays in LLC-PK1 cells. All viral culture and infection experiments in this study were conducted in Biosafety Level 2 (BSL-2) laboratories and were approved by the institutional biosafety committee in accordance with national biosafety regulations.

### Antibodies

The following primary antibodies were used in this study: rabbit anti-Rab10 (Abcam, ab237703, IFA: 1:100, WB: 1:1000); Alexa Fluor 488 Anti-Rab10 antibody MJF-R23 (Abcam, ab302654, IFA: 1:50); rabbit anti-SADS-CoV N; mouse anti-SADS-CoV N; mouse anti-SADS-CoV S were prepared and maintained in our laboratory; mouse anti-SARS-CoV-2 N (ABclonal, A20142, WB: 1:1000); rabbit anti-TGN46 (ABclonal, A19618, IFA: 1:100, WB: 1:1000); rabbit anti-LMAN1 (ABclonal, A4941, IFA: 1:100, WB: 1:1000); rabbit anti-LAMP1 (ABclonal, A22194, IFA: 1:100, WB: 1:1000); rabbit anti-Alix (ABclonal, A25326, WB: 1:1000); rabbit anti-CD9 (ABclonal, A1703, WB: 1:1000); rabbit anti-Caveolin-1 (ABclonal, A22417, WB: 1:1000); rabbit anti-Calnexin (Abcam, ab22595, WB: 1:1000); mouse anti-dsRNA (Scicons, #10010500, IFA: 1:100)rabbit anti-GAPDH (Proteintech, #81640–5-AP, WB: 1:10000); rabbit anti-Flag (Proteintech, #80801–2-RR, WB: 1:5000); mouse anti-Flag (Proteintech, #66008–4-Ig, IFA: 1:200, WB: 1:5000); rabbit anti-Myc (Proteintech, #10828–1-AP, WB: 1:4000); mouse anti-HA (ABclonal, AE008, IFA: 1:100, WB: 1:1000). The secondary antibodies used were as follows: Alexa Fluor 488 Goat Anti-Rabbit IgG H&L (Abcam, ab150077, IFA: 1:500); Alexa Fluor 488 Goat Anti-Mouse IgG H&L (Abcam, ab150113, IFA: 1:500); Alexa Fluor 594 Goat Anti-Rabbit IgG H&L (Abcam, ab150080, IFA: 1:500); Alexa Fluor 594 Goat Anti-Mouse IgG H&L (Abcam, ab150116, IFA: 1:500) and Alexa Fluor 647 Donkey Anti-Rabbit IgG H&L (Abcam, ab150075, IFA: 1:500). For immunoblotting, membranes were probed with the indicated primary antibodies, followed by incubation with the following secondary antibodies (LI-COR) diluted at 1:10,000: goat anti-rabbit IRDye 800CW (#926–32211), goat anti-rabbit IRDye 680RD (#926–68071), or goat anti-mouse IRDye 800CW (#926–32210). Blots were visualized and quantified using an Odyssey CLx Imaging System and Image Studio software (version 5.2).

### Rapid reconstruction of SADS-CoV using the yeast homologous recombination platform

The full-length infectious clones pCC1BAC-SADS-CoV, pCC1BAC-SADS-CoV-GFP, and pCC1BAC-SADS-CoV E mutant were generated using a yeast homologous recombination platform. Specifically, the yeast replication and selection elements, consisting of the 2μ ori-TRP1 promoter-TRP1 sequence (2558 bp) from the pGBKT7 vector (Clontech, 630442), were integrated into the pCC1BAC vector. This modification enabled the plasmid to replicate in yeast. Concurrently, the cytomegalovirus (CMV) early promoter, the hepatitis delta virus (HDV) ribozyme sequence, and the bovine growth hormone (BGH) termination signal were inserted into the linearized pCC1BAC vector to create the intermediate plasmid pCC1BAC-CMV-HDVRz-BGH. For the construction of pCC1BAC-SADS-CoV-GFP, eight overlapping cDNA fragments were amplified and purified from SADS-CoV cDNA. A GFP-T2A sequence was introduced at the first start codon of the N gene coding region within the eighth fragment. All fragments possessed homologous arms approximately 50–100 bp in length to facilitate recombination. Fragment amplification was performed using PrimeSTAR Max DNA polymerase (Takara, R045A) according to the manufacturer's guidelines.

The linearized vector and the eight overlapping fragments were co-transformed into MaV203 yeast competent cells (Thermo, 11445012) via a standard yeast plasmid transformation protocol. The transformation mixture was subsequently plated on SD-Trp tryptophan selection agar plates and incubated at 30°C for 48 h to select for positive yeast colonies. Following screening and identification of positive yeast clones, the recombinant plasmids were propagated in DH10B electrocompetent cells (Thermo, 18290015) via electroporation (BioRad, Gene Pulser Xcel) and cultured in LB medium supplemented with 12.5 μg/mL chloramphenicol. The integrity of the obtained infectious clones was verified through full-length sequencing. These verified clones were then transfected into BHK21 cells using Lipofectamine 3000 (Thermo, L3000075) for 48 h, and the rescued virus could subsequently be passaged in Vero E6 cells and harvested for further study.

The recombinant icSADS-CoV-GFP E-Flag genome was generated by inserting a Gly-Ser linker and a FLAG epitope immediately upstream of the native stop codon of the SADS-CoV E gene. The inserted nucleotide sequence, 5′-GGTAGTGACTACAAA GACGATGACGACAAG-3′, encodes GS-DYKDDDDK and is followed by the native TAA stop codon. This C-terminal tagging strategy preserved the E open reading frame, the downstream E–M genomic junction, and the M open reading frame. To generate recombinant viruses carrying mutations in the E gene, the codon corresponding to the indicated amino acid was replaced with an alanine codon or a stop codon, as appropriate. The modified sequences were introduced into the full-length icSADS-CoV-GFP infectious clone, and the recombinant viruses were rescued using our established reverse-genetics system. The modified regions of the rescued viruses were verified by RT-PCR followed by Sanger sequencing.

### Genome-wide CRISPR knockout screen

A human GeCKO CRISPR knockout pooled library containing 123,411 sgRNAs targeting 19,050 human genes was a gift from Feng Zhang (Addgene, #1000000048). The library was amplified in Endura competent cells (Lucigen) and purified using a Plasmid Maxi Kit (Qiagen, #12163). For lentiviral packaging, HEK 293T cells were co-transfected with the sgRNA library plasmid, psPAX2 (Addgene, #12260), and pMD2.G (Addgene, #12259) at a 4:3:2 mass ratio using PEI MAX 40K transfection reagent (Polysciences, 24765). Viral supernatants were collected 48 hpi, concentrated with Lenti-X Concentrator (Clontech, 631231), and stored at -80°C. For the CRISPR screen, HeLa cells were transduced with the packaged lentiviral sgRNA library at an MOI of 0.3. Transduced cells were selected with puromycin for 7 days to generate a stable pooled knockout library. Approximately 1 × 10^8^ library cells were seeded in T175 flasks and inoculated with icSADS-CoV-GFP at an MOI of 0.01. Infection proceeded until near complete cytopathic effect was observed. The medium was then replaced to allow the survival and outgrowth of resistant cell colonies. Surviving cells were harvested, re-plated, and subjected to two additional rounds of identical viral challenge. Following the third round of selection, the resistant cell pool was expanded. Genomic DNA was extracted from approximately 1 × 10^7^ resistant cells isolated by fluorescence-activated cell sorting (FACS). As a control, genomic DNA was also extracted from 1 × 10^7^ uninfected library cells. sgRNA-encoding regions were amplified from the genomic DNA and subjected to deep sequencing on an Illumina NovaSeq 6000 platform. Enriched sgRNA sequences and their corresponding target genes were subsequently identified through bioinformatic analysis (S1 Table).

### RNA interference

All siRNAs were synthesized by Seven Biotech. The target sequences for all siRNAs are listed in Supplementary Table (S2 Table). For transfection, HeLa cells, IPI-2I cells or LLC-PK1 cells were seeded in 12-well plates one day prior to reach 30–50% confluence. Then, 50 nM siRNA was diluted in 100 μL of Opti-MEM medium. The diluted siRNA was mixed with 4 μL Lipofectamine RNAiMAX (Thermo, 13778150) reagent and incubated for 15 min at room temperature to allow complex formation. The resulting siRNA-lipid complexes were then added to the cells. After 36 h of incubation, the cells were subjected to subsequent experimental treatments.

### Generation of Rab10 knockout HeLa cell lines

To generate Rab10-knockout HeLa cells, a human Rab10-targeting sgRNA (5′- catcatccgaaaaacgaaaa-3′) was cloned into the lentiCRISPR v2 vector and packaged into lentiviral particles. Wild-type HeLa cells were then transduced with the resulting lentivirus, followed by isolation of single-cell clones. Successful knockout of the Rab10 gene was confirmed by DNA sequencing and western blot.

### Generation of Dox-inducible cell lines expressing Rab10

Briefly, the cDNA encoding wild-type Rab10 was cloned into the pLV2-TRE3GS-MCS-TetOne-Blast vector to generate lentiviral transfer plasmids. The lentiviruses were then produced by co-transfecting HEK 293T cells with the lentiviral packaging plasmids psPAX2, pMD2.G, and the respective transfer plasmid. The viral supernatant was collected 48 h post-transfection and concentrated 50-fold using a lentiviral concentrator. Rab10-knockout HeLa cells were infected with the concentrated lentiviruses carrying either wild-type Rab10. Transduced cells were selected with blasticidin (5 μg/mL). To induce the expression of wild-type Rab10, cells were treated with 2.5 μg/mL doxycycline for 24 h.

### Cell viability assay

Cell viability was assessed using the Cell Counting Kit-8 (CCK-8, APExBiO, K1018) according to the manufacturer's instructions. Approximately 1 × 10^4^ control or Rab10-knockout HeLa cells were seeded into each well of opaque-walled 96-well plates. After 48 h, CCK-8 reagent was added to each well, followed by incubation for 2 h in a cell culture incubator. Absorbance at 450 nm was then measured using a BioTek ELx808 microplate reader.

### RNA extraction, reverse transcription, and RT-qPCR

Total RNA was extracted from tissue or cell samples using RNAiso EASY (Takara, TCH020) according to the manufacturer's instructions. The extracted RNA was reverse-transcribed into cDNA using the PrimeScript RT Reagent Kit with gDNA Eraser (Takara, RR047A). RT-qPCR was then performed with TB Green Premix Ex Taq II (Takara, RR820A) on a QuantStudio 5 system (Applied Biosystems). Relative gene expression levels were calculated using the GAPDH gene for normalization.

For viral genomic RNA detection, viral RNA was purified from cell culture supernatants using the MiniBEST Viral RNA/DNA Extraction Kit (Takara, 9766). Quantification was conducted by one-step reverse transcription qPCR using the One Step TB Green PrimeScript PLUS RT-PCR Kit (Takara, RR096A) on the QuantStudio 5 system, with absolute quantification performed based on a standard curve. All primer sequences used for RT-qPCR are listed in the Supplementary Table (S2 table).

### Transmission electron microscopy (TEM)

For ultrastructural examination, HeLa cells were either mock-infected or inoculated with SADS-CoV at an MOI of 1. At 24 hpi, cells were subjected to primary fixation with 2% glutaraldehyde for 30 min at room temperature. Following three washes in 0.1 M sodium cacodylate buffer, the samples underwent secondary fixation on ice using a solution of 1% (w/v) reduced osmium tetroxide, 0.1 M sodium cacodylate, and 1.5% (w/v) cyanoferrate. After thorough rinsing with buffer and distilled water, the specimens were dehydrated through a graded ethanol series and embedded in resin. Ultrathin sections (50–70 nm) were then prepared using a diamond knife and collected on Formvar-coated copper grids. For contrast enhancement, the grids were double-stained with 2% uranyl acetate (5 min) and lead citrate (2 min). Final imaging was performed at various magnifications (1,500 × to 12,000 ×) using a HITACHI H7650 transmission electron microscope.

### VLPs purification

HeLa WT or HeLa *Rab10*^*-/-*^ KO1 cells were co-transfected with either pcDNA3.1-SARS-CoV-2-N-T2A-M-IRES-E or pcDNA3.1-SADS-CoV-N-T2A-M-IRES-E. At 48 h post-transfection, cell culture supernatants were harvested, filtered through 0.22 μm membranes, and clarified by centrifugation (10,000 × *g*, 30 min, 4°C). For the drug inhibition assay, DMSO, BFA (MCE, HY-16592; 5 μM), or CID-1067700 (MCE, HY-13452; 40 μM) were added to the culture medium at 12 h post-transfection, and supernatants were collected at 48 h post-transfection. To concentrate VLPs, the clarified supernatants were subjected to ultracentrifugation (28,000 × *g*, 2 h, 4°C) over a 20% sucrose cushion. The resulting VLPs pellets were resuspended in phosphate-buffered saline (PBS) buffer for western blot.

### Isopycnic gradient centrifugation and rate-zonal centrifugation in sucrose gradients

For differential centrifugation, the cell culture supernatant from SADS-CoV-infected cells was first clarified by centrifugation at 1,000 × *g* for 10 min at 4°C to remove cells and debris. The supernatant was further cleared by centrifugation at 10,000 × *g* for 30 min at 4°C. The clarified supernatant was then layered onto a 20% (w/v) sucrose cushion and pelleted by ultracentrifugation at 100,000 × *g* for 2 h. The resulting pellet was gently resuspended in PBS and incubated overnight at 4°C with mild agitation. The resuspended material was analyzed by western blot.

For rate-zonal centrifugation, the pellet obtained from differential centrifugation was resuspended and layered onto a continuous 10–60% (w/v) sucrose gradient. The gradient was centrifuged at 167,000 × *g* for 2 h at 4°C using a 55Ti rotor in an Optima XPN-100 ultracentrifuge (Beckman Coulter). Following centrifugation, approximately 50 fractions were collected sequentially from the top of the gradient. The distribution of viral and cellular marker proteins across the gradient was assessed by western blot analysis of individual fractions.

### ELISA for detection of Rab10 in cell culture supernatant

The concentration of Rab10 protein in cell culture supernatant was quantified using a sandwich enzyme-linked immunosorbent assay (JL20386, Jonlnbio, China). Supernatants were collected 24 h post-transfection and corresponding treatments. The samples were centrifuged at 1,000 × *g* for 20 min, and the resulting supernatant was diluted 50-fold. Subsequently, 100 μL of the diluted sample was added to the ELISA microplate wells pre-coated with a Rab10 capture antibody and incubated at 37°C for 1 h. Following incubation and washing, 100 μL of biotin-labeled detection antibody was added to each well and incubated at 37°C for 1 h. After another wash step, 100 μL of horseradish peroxidase (HRP)-conjugated streptavidin was added and incubated at 37°C for 30 min. The plate was then thoroughly washed to remove unbound conjugates. For color development, 90 μL of tetramethylbenzidine (TMB) substrate was added to each well at 37°C for 15 min. The enzymatic reaction was stopped by adding 50 μL of stop solution. The absorbance was immediately measured at a wavelength of 450 nm using a microplate reader (BioTek ELx808, USA), and the sample concentration was calculated based on the standard curve.

### Western blot and Co-IP assay

Cells were lysed in RIPA buffer (Beyotime, P0013B) containing PMSF. The lysates were mixed with SDS-PAGE sample loading buffer (Beyotime, P0015), boiled at 100°C for 10 min, separated by SDS-PAGE, and transferred onto nitrocellulose membranes. For the Co-IP assay, cell lysates were incubated with tag-specific antibodies at 4°C for 4 h, followed by incubation with Protein A/G beads (Thermo, 88802) for 6 h. After washing with IP washing buffer, the beads were boiled in loading buffer and the proteins were resolved by SDS-PAGE. Membranes were blocked with 5% non-fat milk in TBST for 1 h at room temperature and then incubated with primary antibodies overnight at 4°C. After washing, the membranes were probed with appropriate IRDye-conjugated secondary antibodies for 1 h at room temperature. Signals were detected using an Odyssey CLx imaging system (LI-COR) at 680 and 800 nm, and band intensities were quantified with Image J. Information on specific antibodies can be found in the antibody section of Materials and methods.

### IFA and confocal microscopy

HeLa WT or *Rab10*^-/-^ KO cells grown on 35-mm glass-bottom dishes (Cellvis, D35-10–1-N) were infected with SADS-CoV. For immunofluorescence, cells were fixed with 4% paraformaldehyde for 30 min (ensuring complete virus inactivation), permeabilized with 0.3% Triton X-100 for 10 min at 4°C, and blocked with 5% BSA for 1 h at room temperature. Subsequently, cells were incubated overnight with primary antibodies, followed by appropriate secondary antibodies for 1 h. Information on specific antibodies can be found in the antibody section of Materials and methods. Nuclei were stained with DAPI (Beyotime, C1002). Images were acquired using a Zeiss LSM880 confocal microscope equipped with a 60 × oil-immersion objective. For each experiment, 15–20 random fields were recorded. Maximum intensity projections were generated to analyze protein subcellular localization. Colocalization was quantified using Mander's correlation coefficient or Pearson's correlation coefficient with Zeiss analysis software.

### Gaussia luciferase assay

Gaussia luciferase activity was measured using the Gaussia-Lumi Gaussia Luciferase Assay Kit (Beyotime, RS072S). HeLa cells were transfected with the pGluc Gaussia luciferase reporter plasmid (Beyotime, D2098). After treatment with DMSO or BFA, cell culture supernatants were collected, and Gaussia luciferase activity was analyzed according to the manufacturer's instructions. Briefly, 100 μL of supernatant was collected from treated wells into a 96-well plate, followed by the addition of 100 μL of Gaussia-Lumi detection working solution per well and thorough mixing. After incubation at room temperature for 10 min, chemiluminescence was measured using an Enspire multimode plate reader (PerkinElmer, USA).

### Statistical analyses

Analysis of experimental data, standard deviation (SD) calculations, and graph plotting from the results of three independent experiments were performed using GraphPad Prism software (version 9.0). All statistical analyses were performed using either one-way ANOVA with Dunnett's test or an unpaired t-test. Significance levels are as follows: ^*^P < 0.05, ^**^P < 0.01, ^***^P < 0.001, ^****^P < 0.0001, ns, not significant.

## Supporting information

S1 Fig Characterization and validation of the recombinant icSADS-CoV-GFP reporter virus. (A) Replication kinetics of WT and icSADS-CoV-GFP in Vero E6 cells. Vero E6 cells were inoculated with WT or icSADS-CoV-GFP at an MOI of 0.01. After 2 h of adsorption, the inoculum was removed, cells were washed three times with DMEM, and further cultured in DMEM containing 5 μg/mL trypsin. Supernatants were collected at the indicated time points (0–60 hpi), and viral titers were determined by TCID_50_ assay on Vero E6 cells. **(B)** Immunofluorescence analysis of icSADS-CoV-GFP infection. Vero E6 cells were infected with the reporter virus, and expression of GFP (green) and SADS-CoV N protein (red) was visualized at 36 hpi. The lower panel shows representative images of positive cells from three independent experiments. Scale bar, 275 μm. **(C)** Plaque morphology of the recombinant icSADS-CoV-GFP virus. Plaque assay was performed on Vero E6 cells and stained at 3 days post-infection. **(D)** Direct fluorescence observation of HeLa cells infected with icSADS-CoV-GFP (MOI = 0.05), at 24 hpi. Green signal indicates GFP fluorescence. Representative images from three independent experiments are shown. **(E)** Western blot analysis of viral protein expression in HeLa cells infected with icSADS-CoV-GFP. Cell lysates were harvested at 8, 16, and 24 hpi in the presence or absence of trypsin. Membranes were probed with anti-GFP and anti-SADS-CoV N antibodies. Red arrows indicate the uncleaved GFP-2A-N polyprotein, cleaved GFP, and cleaved SADS-CoV N protein. GAPDH served as a loading control. **(F)** Transmission electron micrographs of viral particles purified from Vero E6 cells infected with either WT SADS-CoV or icSADS-CoV-GFP. Black arrows indicate representative, intact SADS-CoV virions. Scale bar, 200 nm. Data are presented as means ± SD from three independent experiments. Statistical significance was analyzed by unpaired t-test. ^*^P < 0.05, ^**^P < 0.01, ^***^P < 0.001, ^****^P < 0.0001, ns, not significant.
(TIF)

S2 Fig SADS-CoV infection reduces the endogenous Rab10 protein abundance without affecting Rab10 mRNA expression, and Rab10 colocalizes with the viral protein in HeLa cells. (A) HeLa cells were mock-infected or infected with icSADS-CoV-GFP and harvested at 12, 24, 36, and 48 hpi. The protein levels of SADS-CoV nucleocapsid protein (N), Rab10, and GAPDH were analyzed by western blot. GAPDH served as the loading control. The relative Rab10 protein levels were determined by densitometric analysis, normalized to GAPDH, and expressed relative to the corresponding mock-infected group. **(B)** Relative Rab10 mRNA levels in mock- or SADS-CoV-infected HeLa cells at the indicated time points were measured by RT-qPCR. Data are presented as means ± SD from three independent experiments. Statistical significance was analyzed by unpaired t-test. ^*^P < 0.05, ^**^P < 0.01, ^***^P < 0.001, ^****^P < 0.0001, ns, not significant. (C) Representative confocal microscopy images of mock- or SADS-CoV-infected HeLa cells. Rab10 was detected in green, the SADS-CoV spike protein (S) in red, and nuclei were counterstained with DAPI in blue. The enlarged image on the right show's areas of Rab10 and S-protein colocalization, indicated by white arrowheads. Scale bars, 5 μm.
(TIF)

S3 Fig Depletion of Arl8b, Rab7, and VPS39 suppresses SADS-CoV replication, while inhibition of ERGIC–Golgi and lysosomal trafficking impairs viral particle release. (**A–B)** Representative immunofluorescence images of HeLa WT cells (A)and HeLa *Rab10*^*-/-*^ KO1 cells (B) transfected with siNC or siRNAs targeting Arl8b, Rab7, or VPS39, followed by infection with icSADS-CoV-GFP. Scale bars, 275 μm. **(C–D)** RT-qPCR analysis of relative SADS-CoV RNA levels in HeLa WT cells (C) and *Rab10*^*-/-*^ KO1 cells (D) following knockdown of Arl8b, Rab7, or VPS39. Viral RNA levels were normalized to those in siNC-transfected cells. **(E–G)** Validation of siRNA-mediated knockdown efficiency. The relative mRNA levels of Arl8b (E), Rab7 (F), and VPS39 (G) were measured by RT-qPCR and normalized to those in siNC-transfected cells. **(H)** Representative immunofluorescence images of icSADS-CoV-GFP infected cells treated with vehicle control (DMSO), Brefeldin A (BFA; 5 μM), or the CID-1067700 (40 μM). Scale bars, 275 μm. **(I)** SADS-CoV VLPs secretion assay. HeLa cells were transfected with VLPs plasmids (N/M/E) following treatment with DMSO, BFA, or CID-1067700. Cell lysates and concentrated medium samples were analyzed by western blot for SADS-CoV N. The ratio of N in medium versus lysate is shown below. **(J)** Experimental timeline for CID-1067700 treatment. HeLa cells were infected with SADS-CoV, and CID-1067700 was added at the indicated time points (2, 4, 6, 8 h) before samples were harvested at 12 h. **(K)** Extracellular SADS-CoV RNA copy of infected cells treated with DMSO, BFA, or CID-1067700 for the indicated durations. Data points represent individual biological replicates (n = 3). Data are presented as means ± SD from three independent experiments. C–D, one-way ANOVA with Dunnett's test; E**–**G, K, unpaired t-test. ^*^P < 0.05, ^**^P < 0.01, ^***^P < 0.001, ^****^P < 0.0001, ns, not significant.
(TIF)

S4 Fig The C-terminal region of SADS-CoV E protein is essential for viral rescue and propagation. (A) Schematic representation of the SADS-CoV genome for the E-WT and truncation mutants. Mutations introducing premature stop codons (TAA) at amino acid positions 72, 68, or 64 are indicated. E-WT is labeled as “Rescued,” while E Δ73–75aa, E Δ69–75aa, and E Δ65–75aa are labeled as “non-rescued.” **(B)** Representative fluorescence images of the rescue and propagation of recombinant icSADS-CoV-GFP E-WT and truncation mutants (E Δ73–75aa, E Δ69–75aa, and E Δ65–75aa). Images show Passage 0 and Passage 1 of the viruses following transfection of infectious clone plasmids. Green signal represents GFP expression. Only the E-WT virus was successfully rescued and propagated to Passage 1. Scale bar, 275 μm. **(C)** Sequence identification of the icSADS-CoV-GFP E truncation mutant clones. The nucleotide sequence alignments confirm the presence of the TAA stop codon at the designated locations for E Δ73–75aa, E Δ69–75aa, and Δ65–75aa.
(TIF)

S1 Table List of genes and sgRNA read counts from the SADS-CoV knockout screen analysis. (XLSX)

S2 Table Sequences of siRNAs for RNA interference, and primers for sgRNA knockout plasmid construction and RT-qPCR experiments. (XLSX)

S3 Table Source data for the experiments. (XLSX)

S1 Raw gel Raw gel from all the western blot images in the study. The raw gel follow the same order as presented in figures and supporting information.
(PDF)
