# Supplementary material for: Rab10 coordinates SADS-CoV non-lytic egress through the ERGIC-TGN-lysosome trafficking pathway
Source: PLoS Pathog. 2026 Sep 1;22(9):e1014569. doi: 10.1371/journal.ppat.1014569 (PMC13561426; doi:10.1371/journal.ppat.1014569)

Uncropped gels for Western Blots in Fig 2.

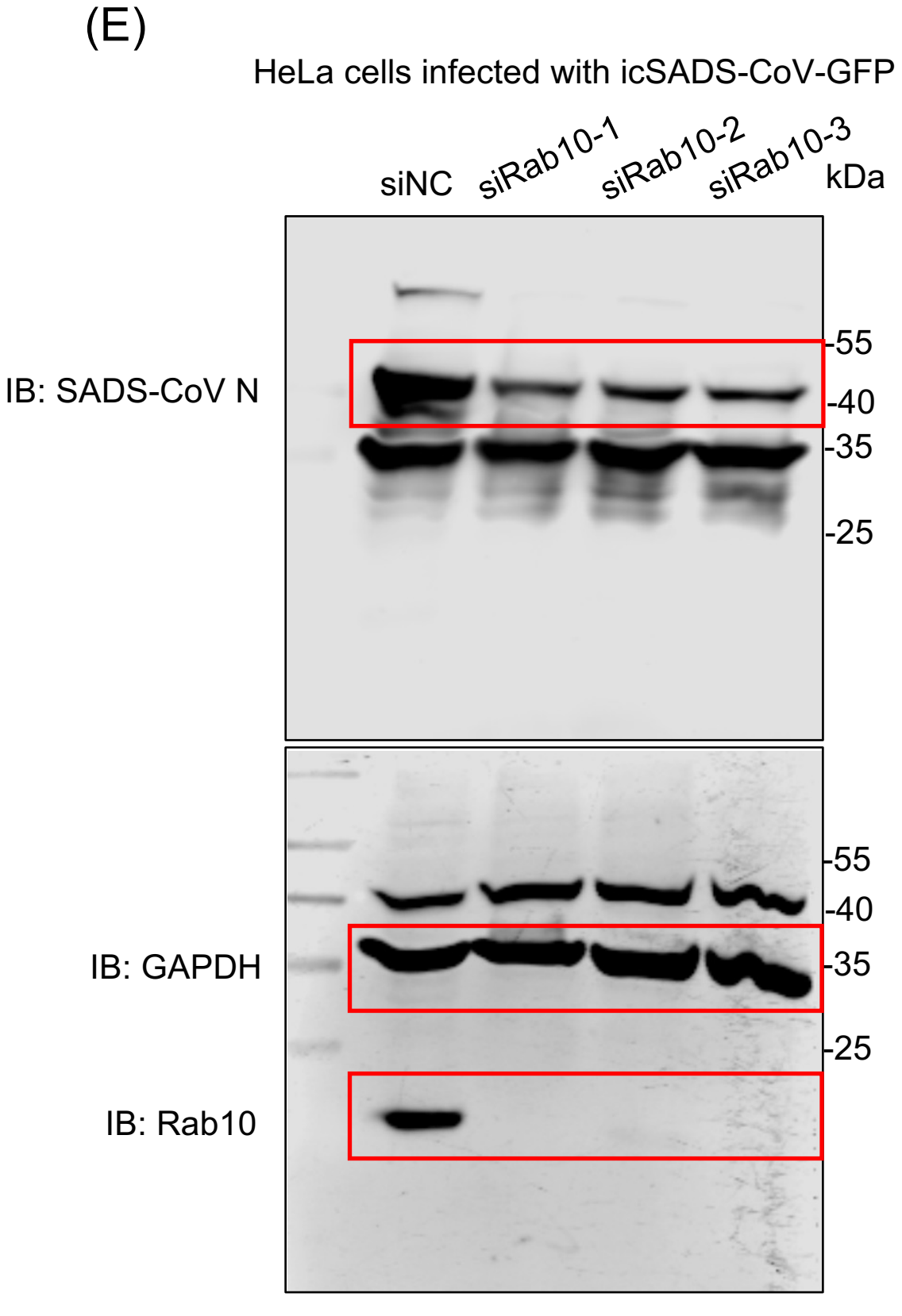

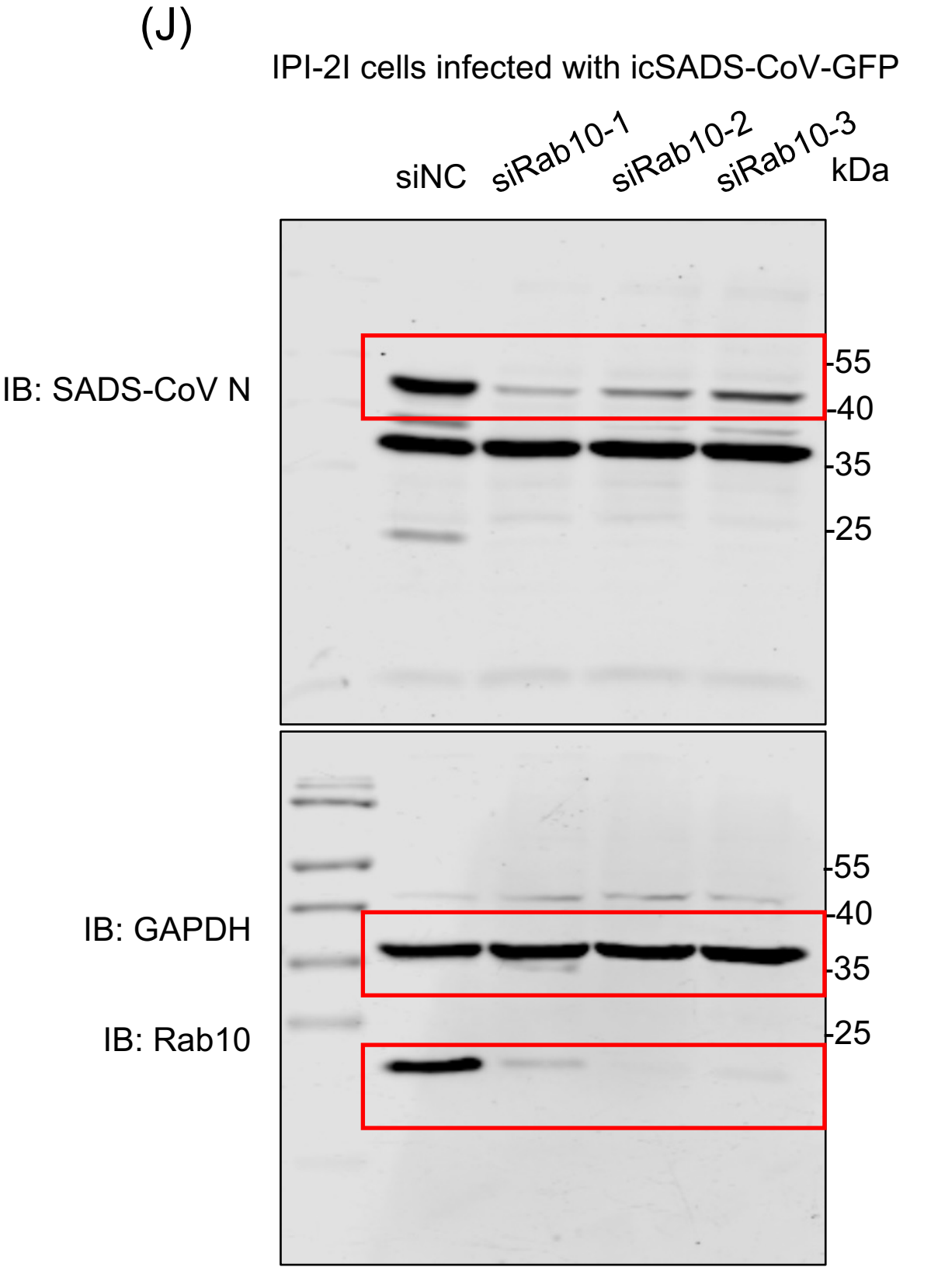

Uncropped gels for Western Blots in Fig 2.

(P)

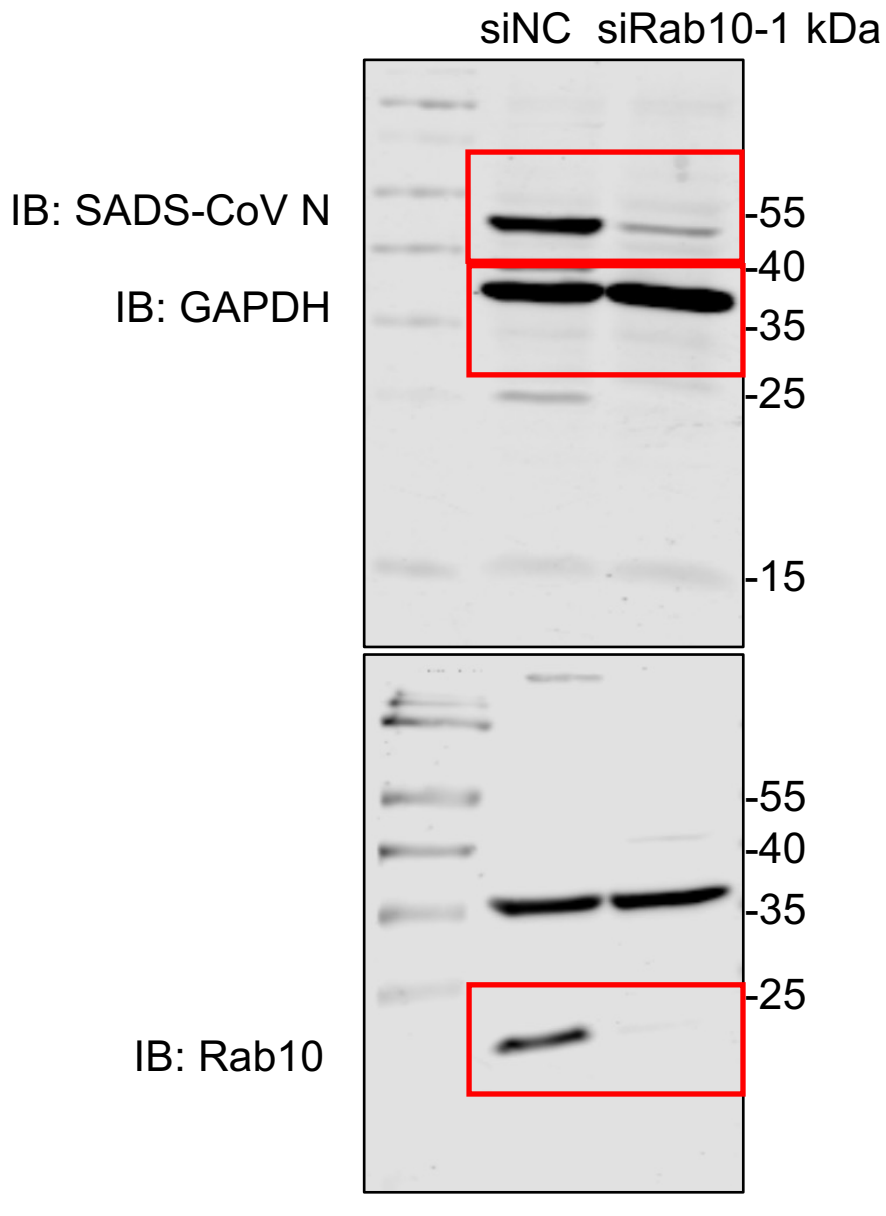

(Q)

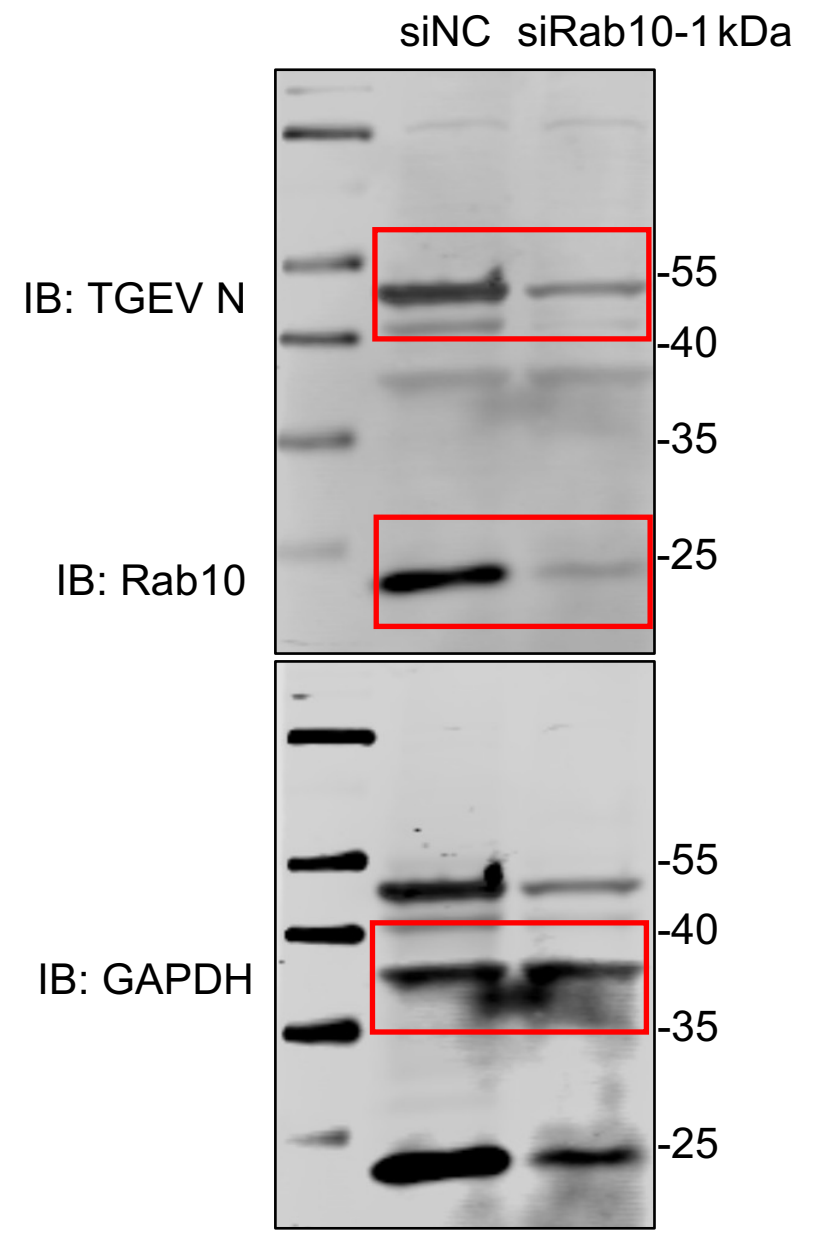

(R)

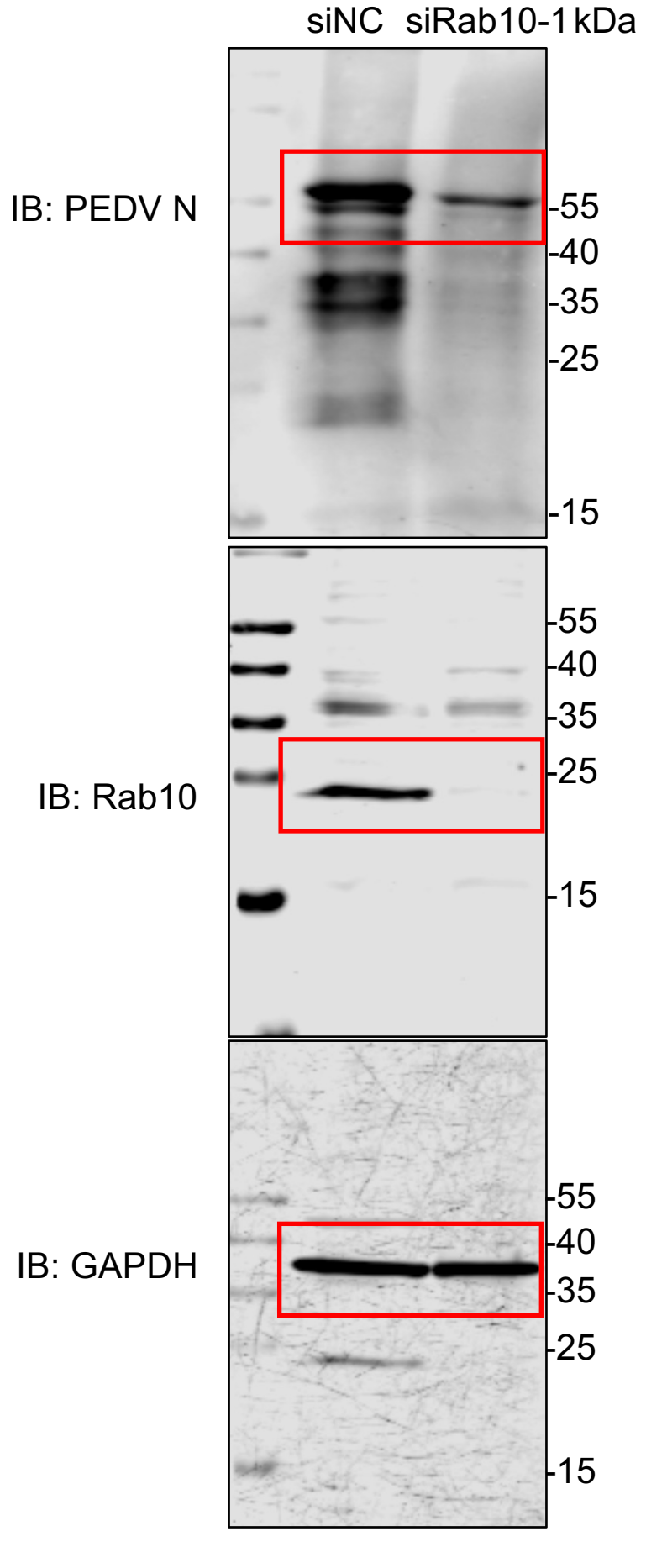

(S)

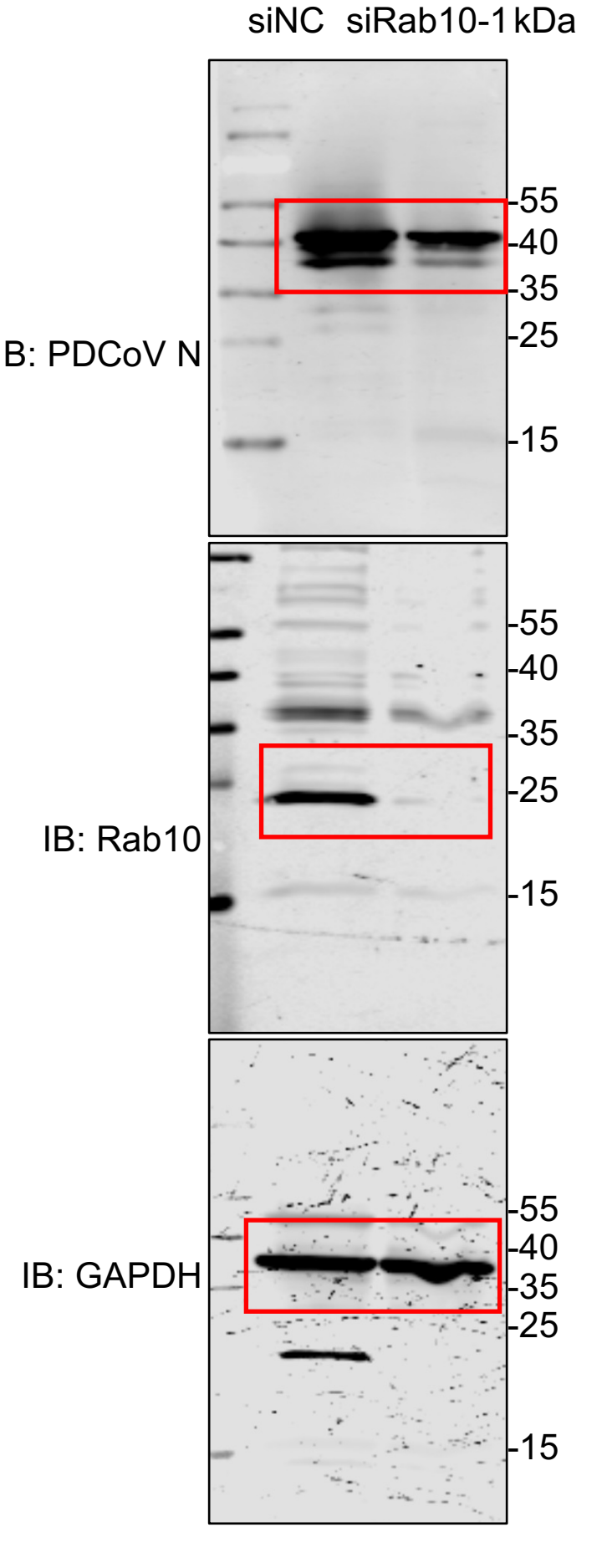

Uncropped gels for Western Blots in Fig 3.

(E)

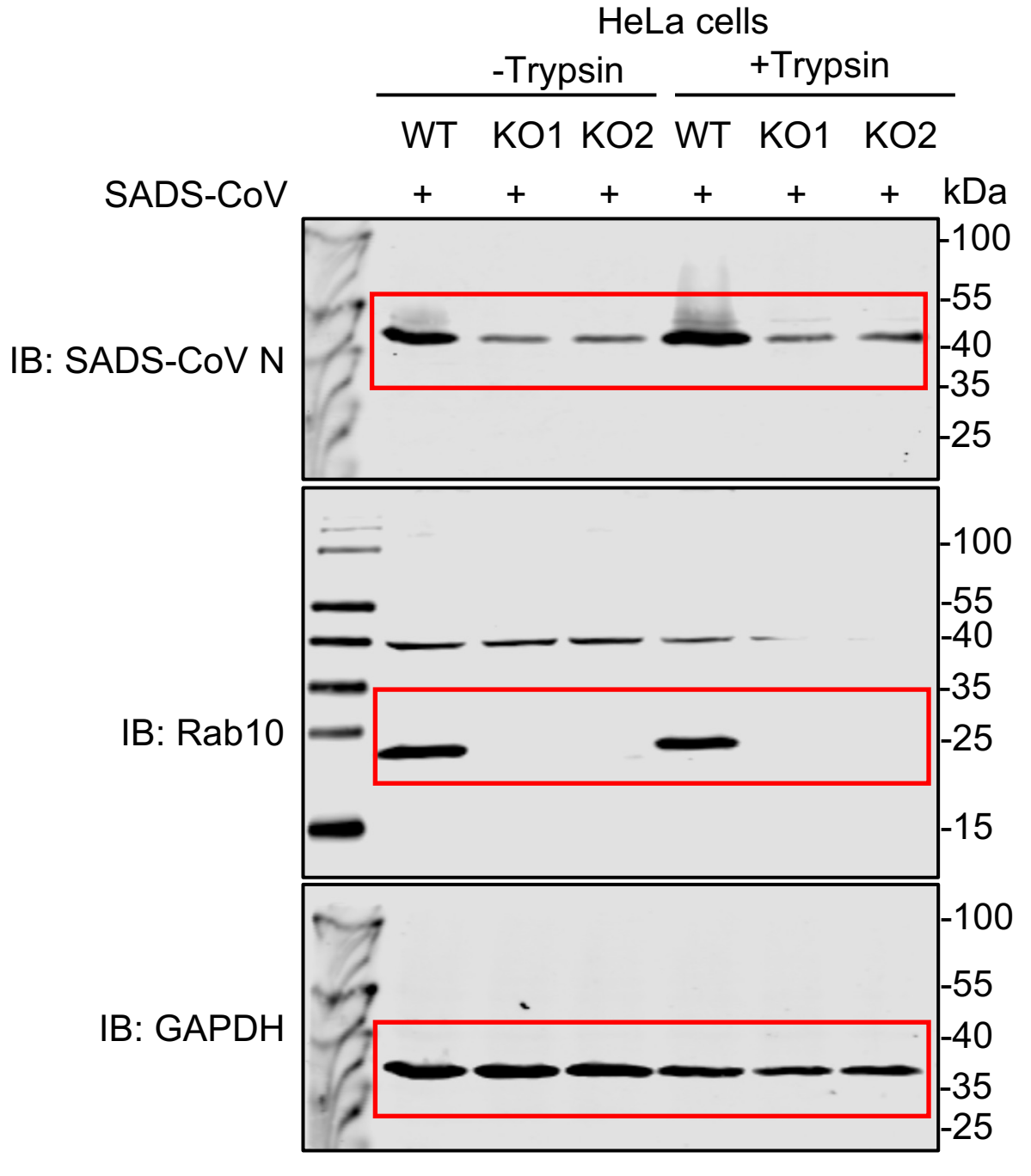

(I)

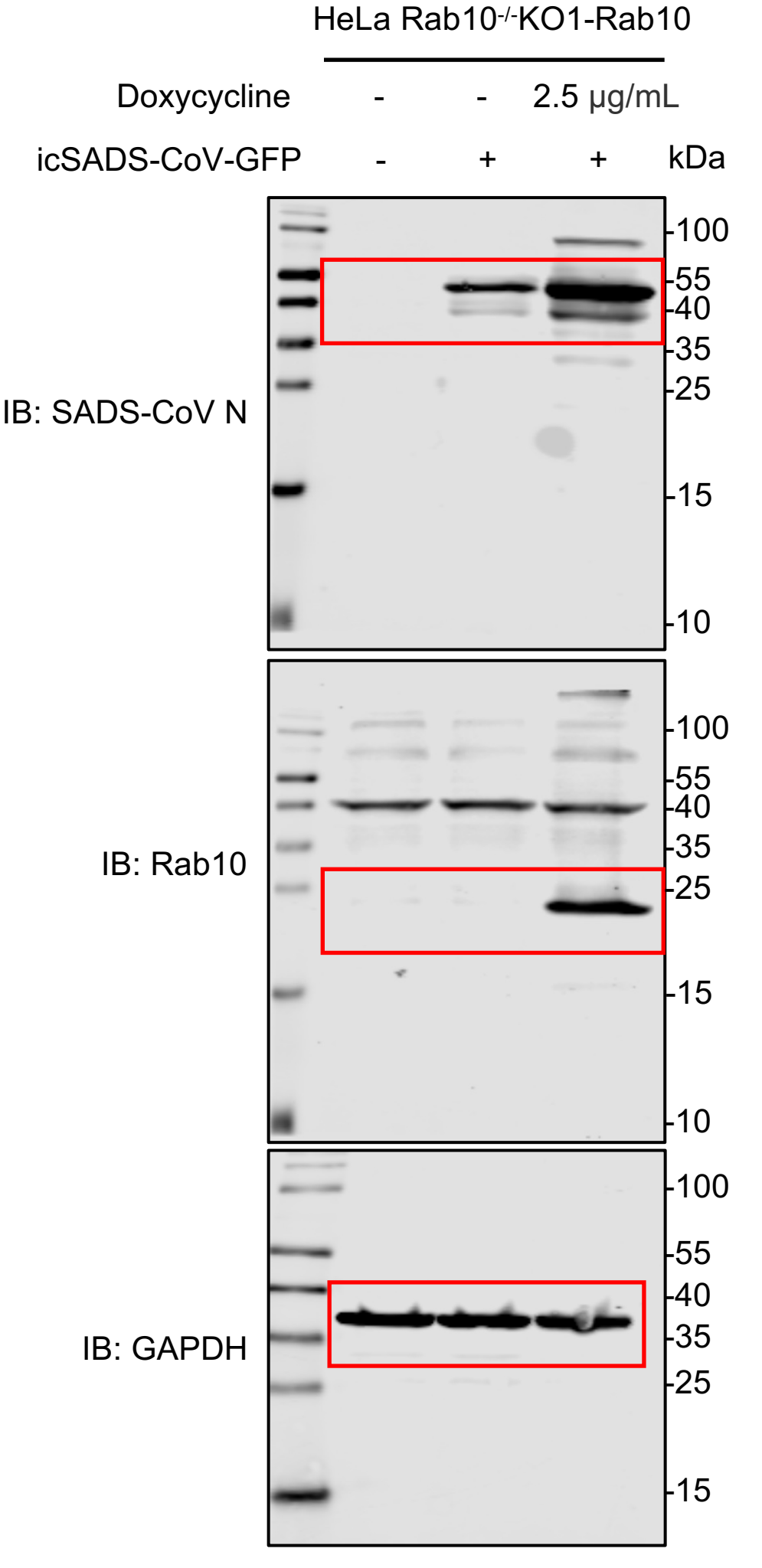

Uncropped gels for Western Blots in Fig 4.

(L)

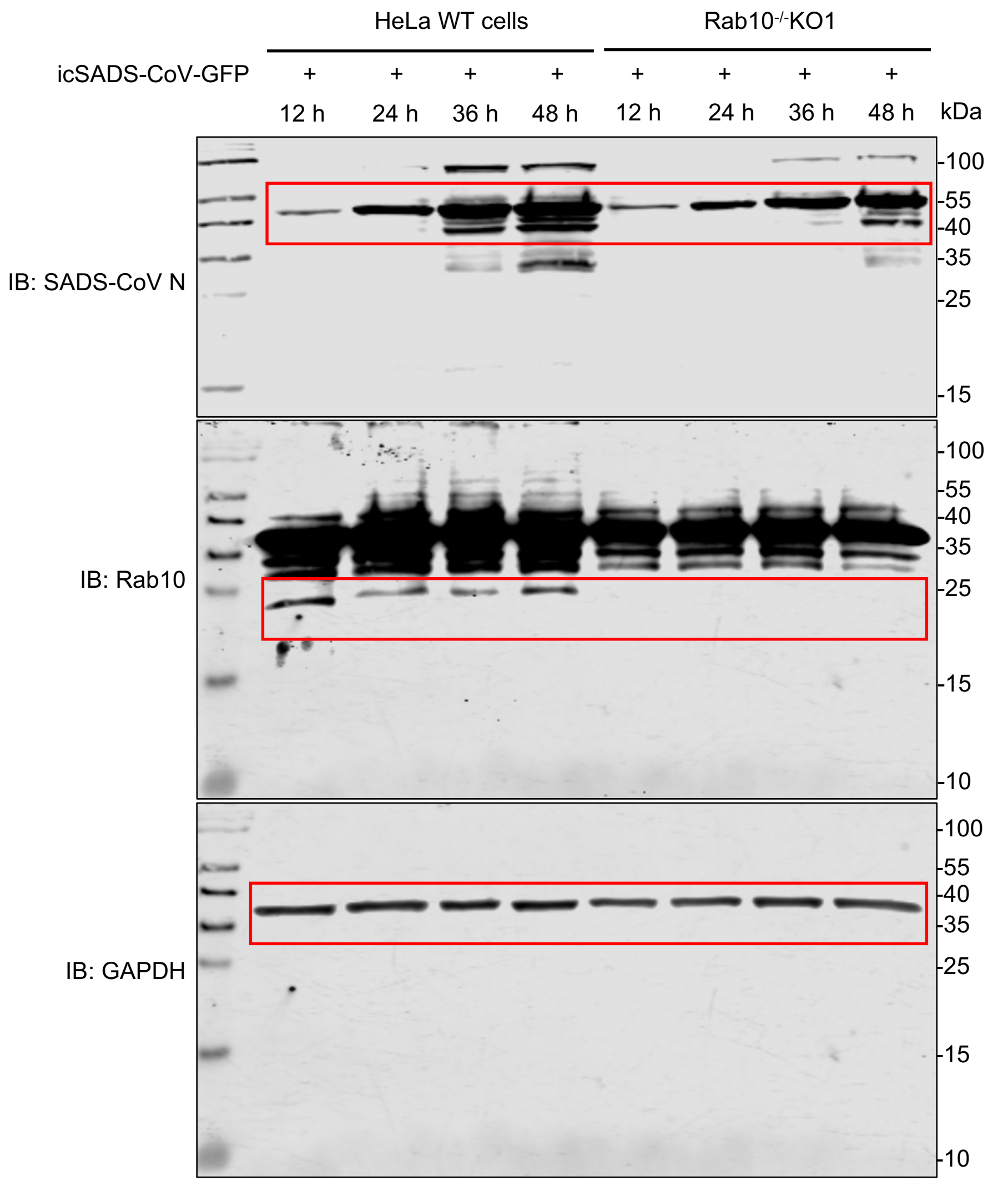

Uncropped gels for Western Blots in Fig 5.

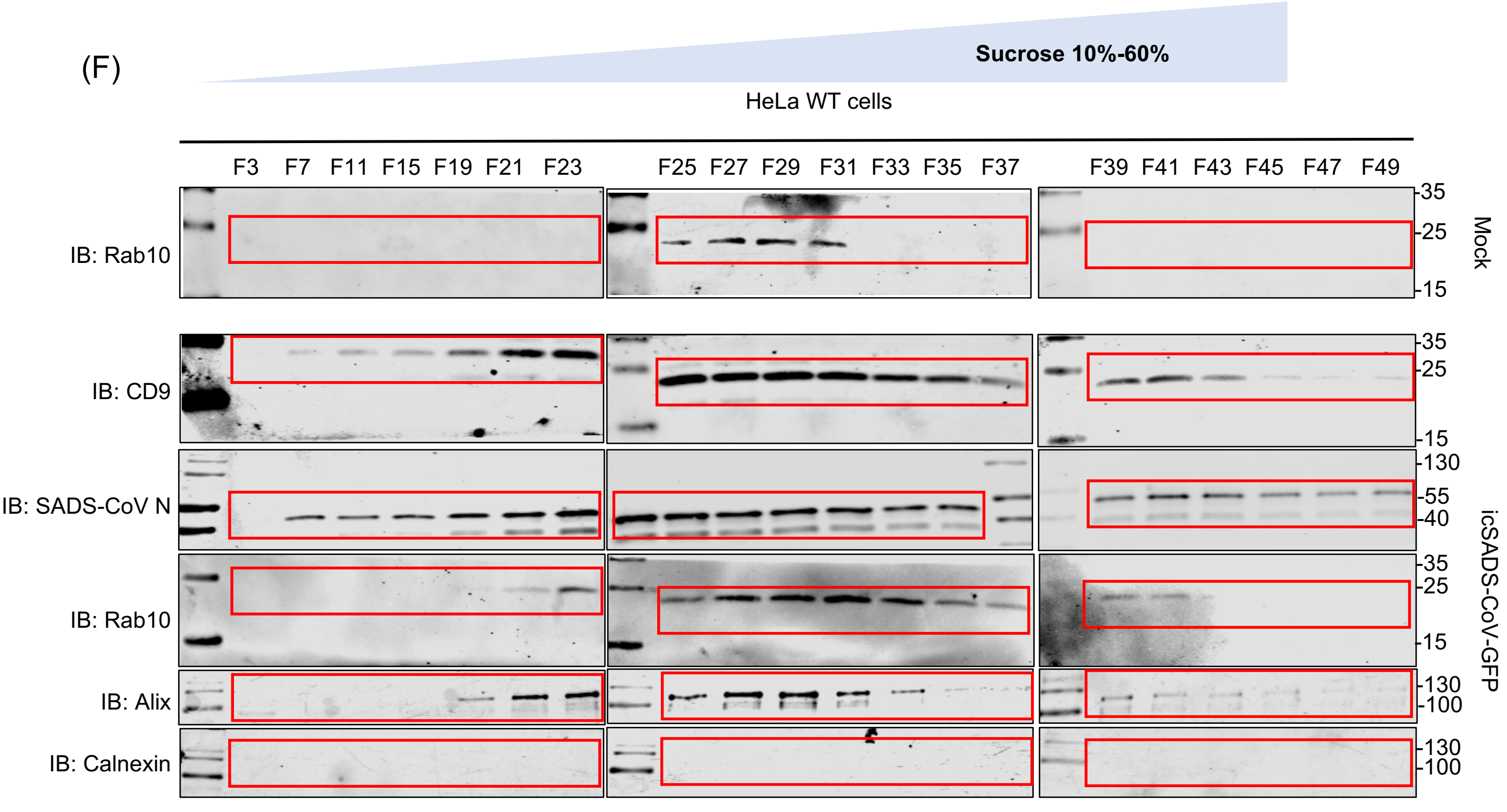

Uncropped gels for Western Blots in Fig 6.

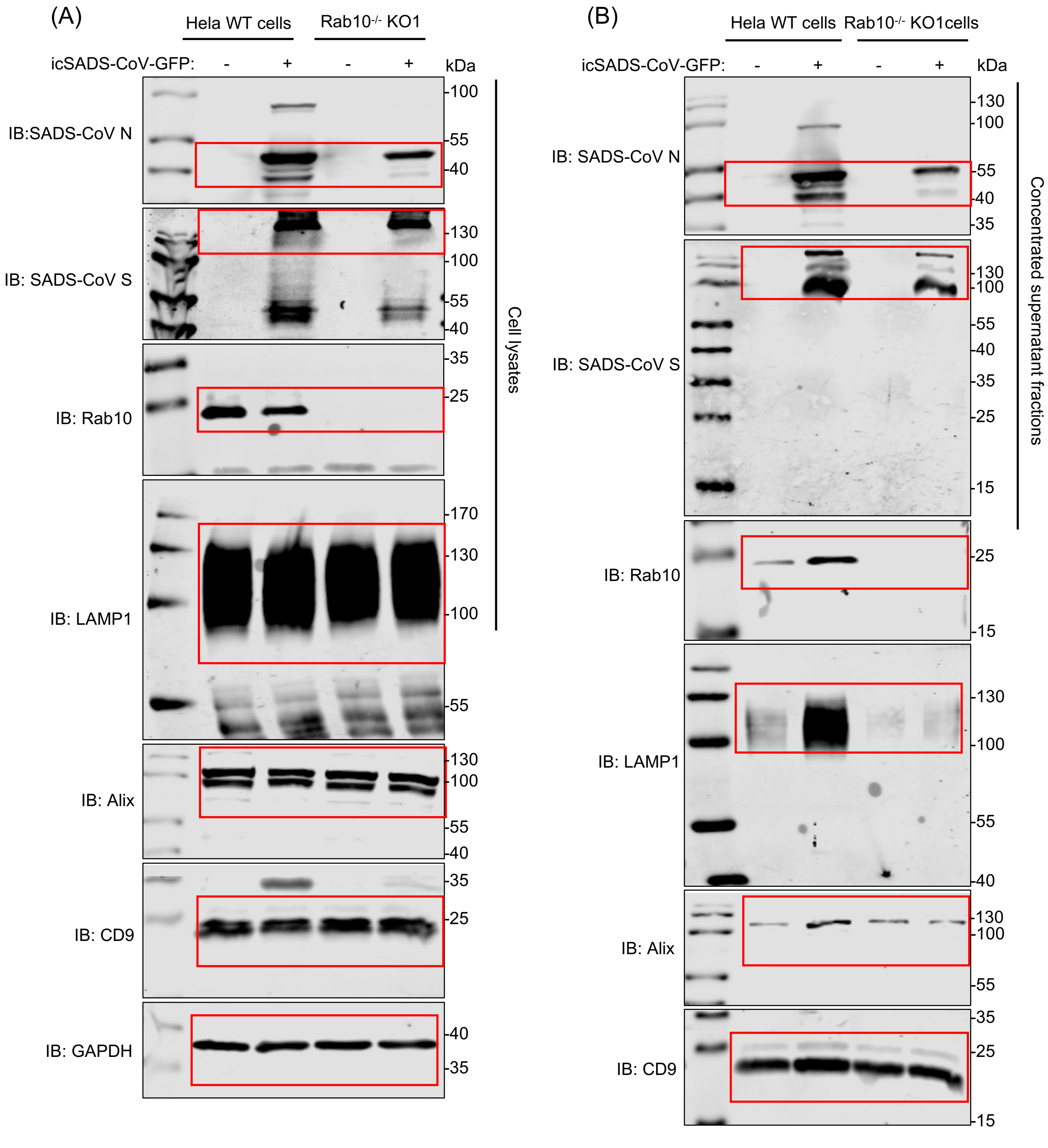

Uncropped gels for Western Blots in Fig 6.

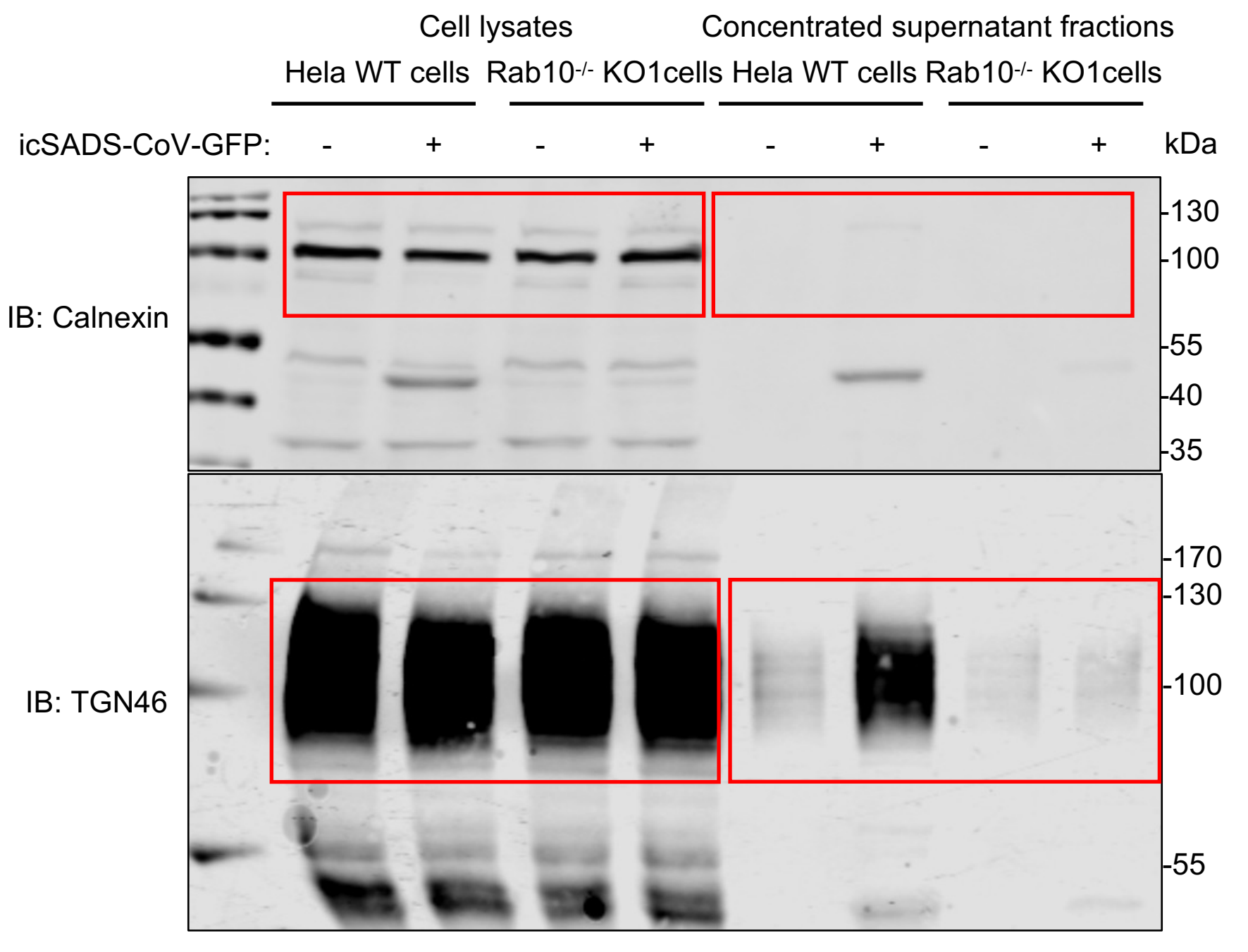

Uncropped gels for Western Blots in Fig 7.

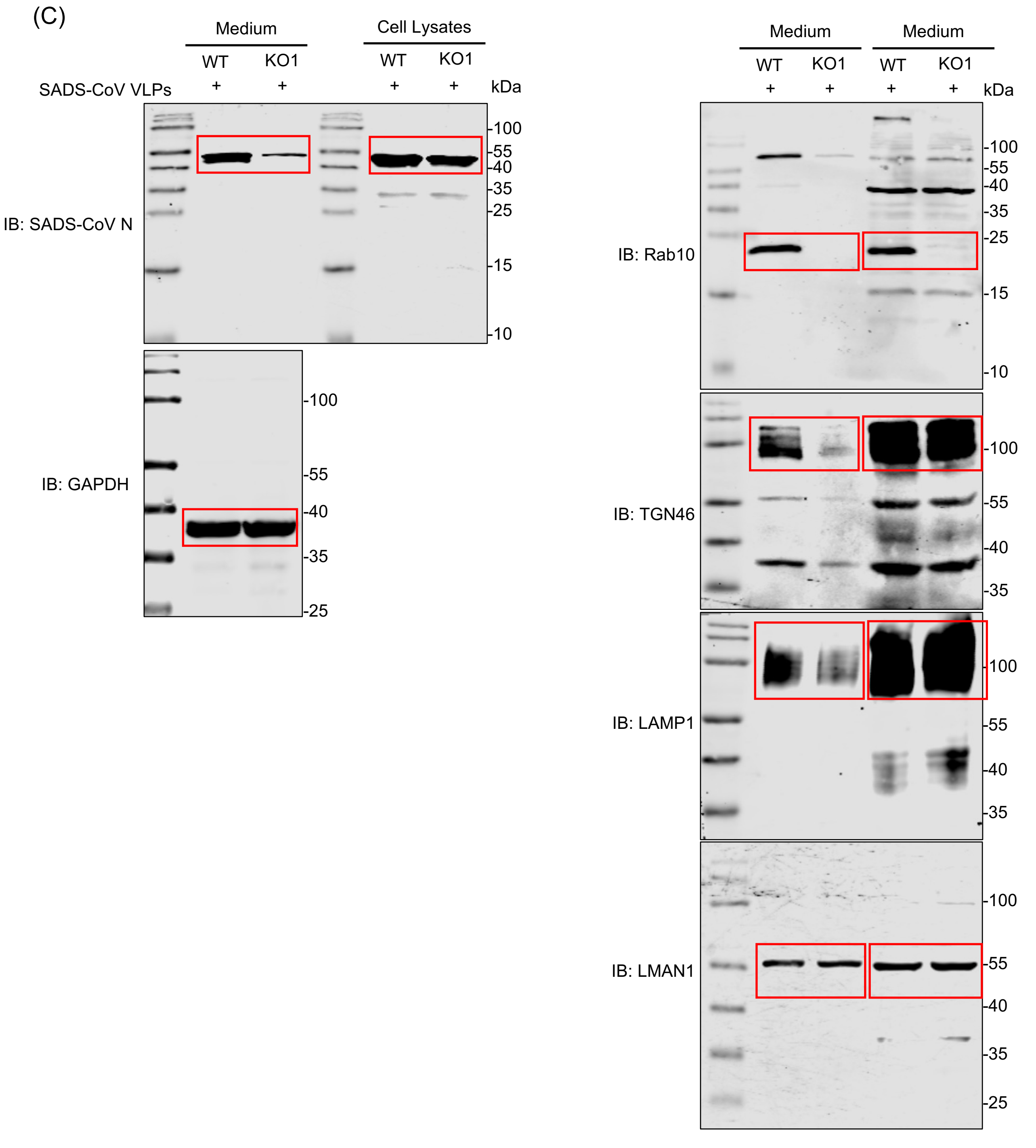

Uncropped gels for Western Blots in Fig 7.

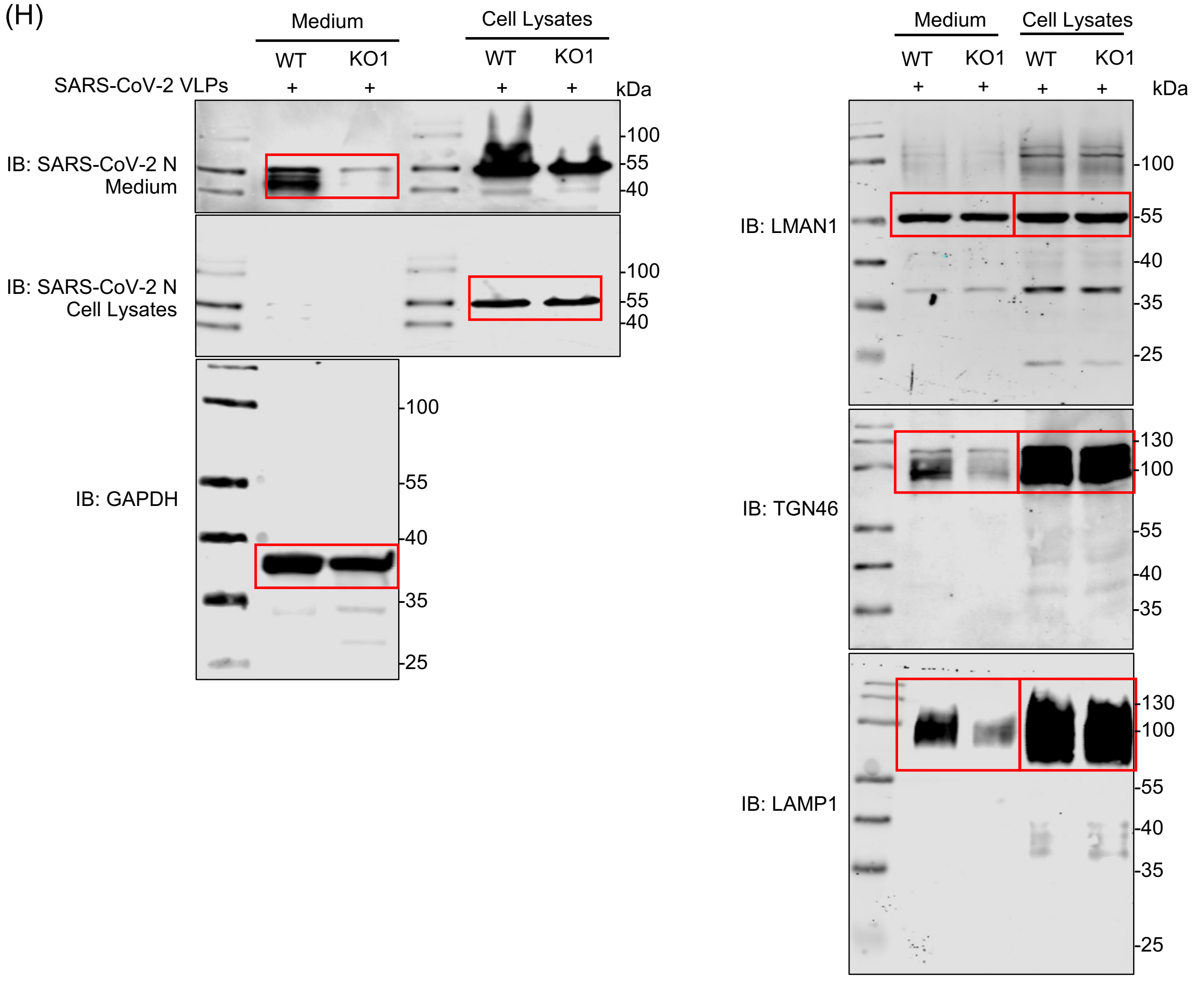

Uncropped gels for Western Blots in Fig 8.

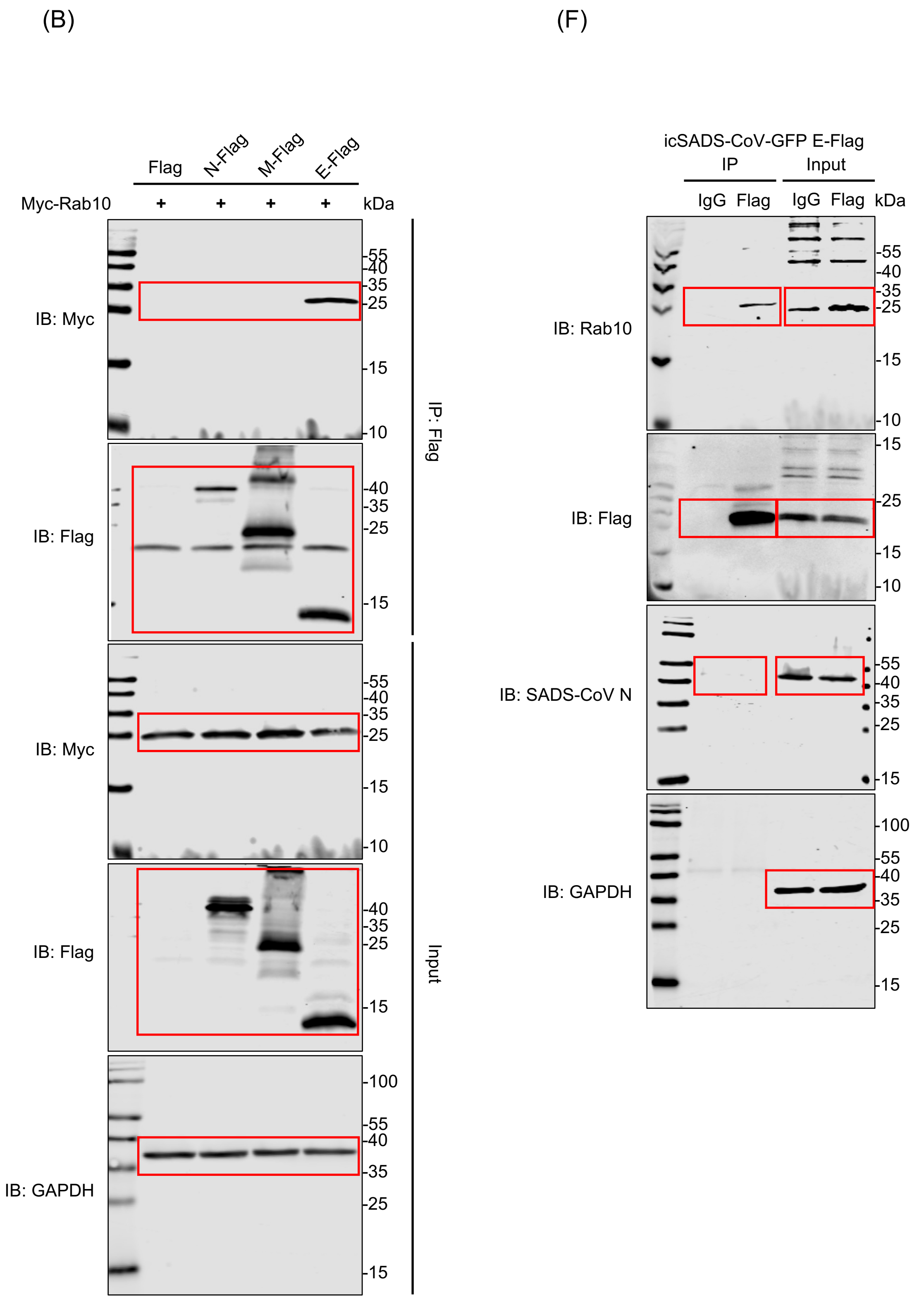

Uncropped gels for Western Blots in Fig 8.

(G)

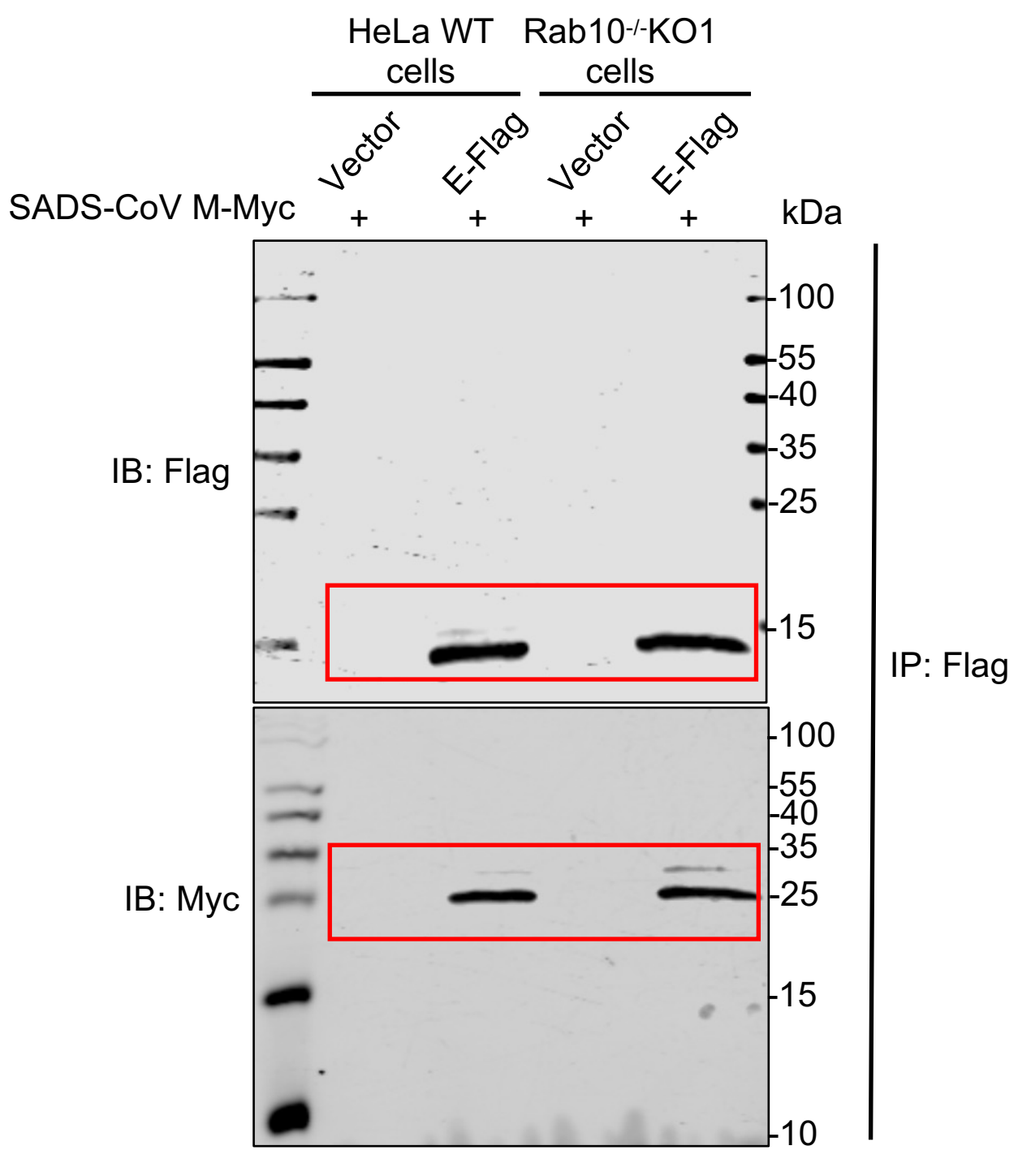

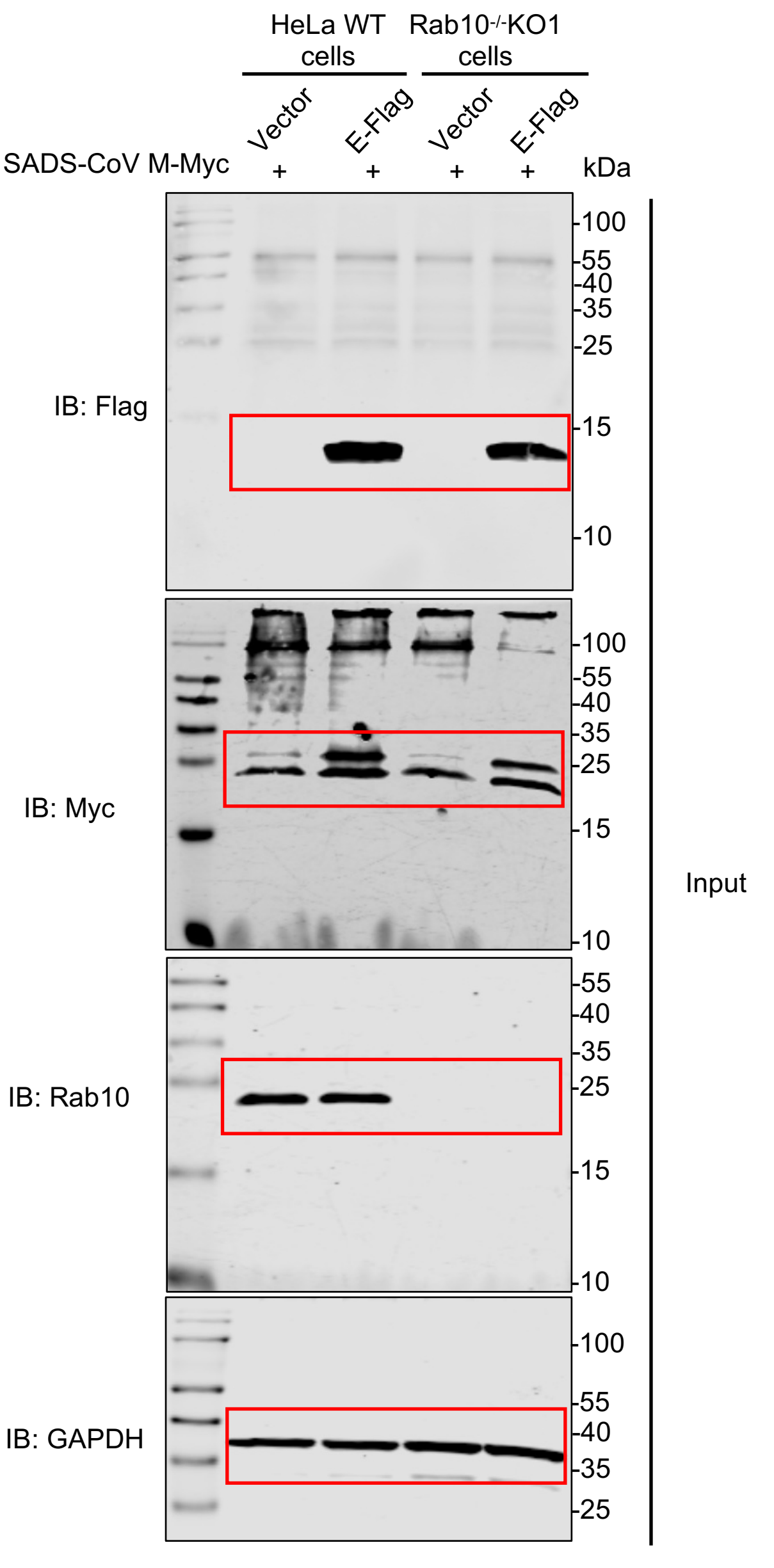

Uncropped gels for Western Blots in Fig 10.

(B)

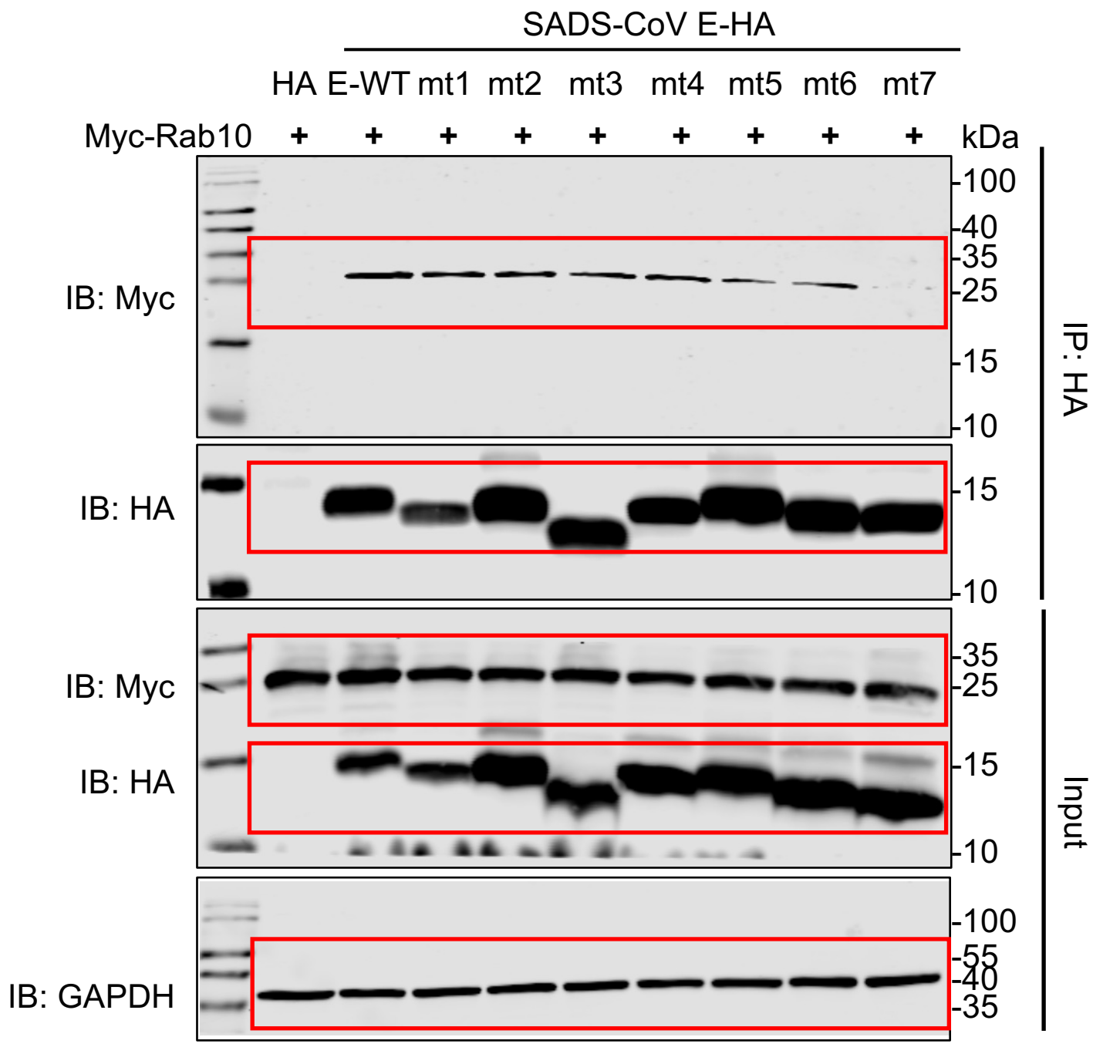

Uncropped gels for Western Blots in Fig 11.

(A)

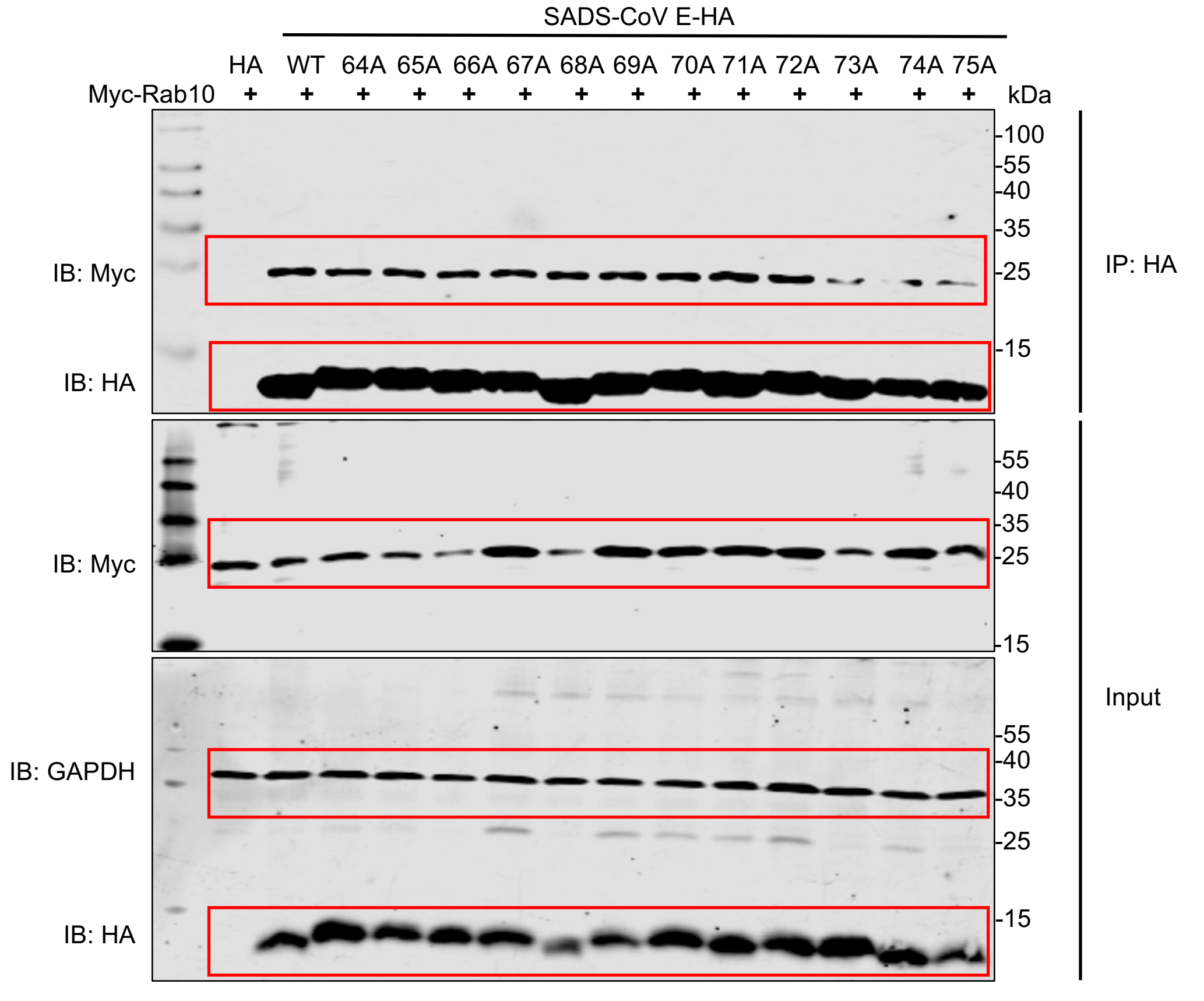

Uncropped gels for Western Blots in Fig 11.

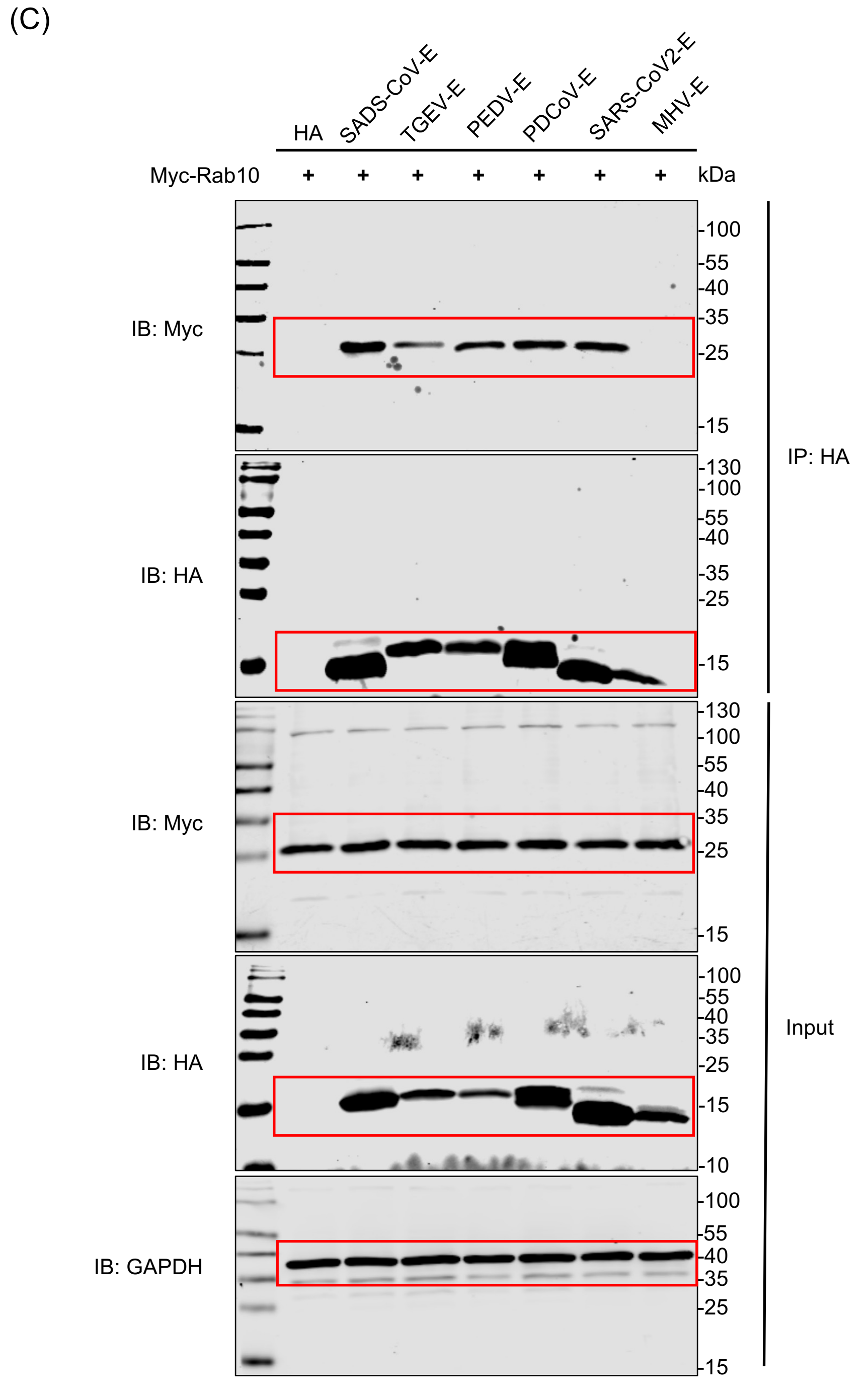

Uncropped gels for Western Blots in Fig 11.

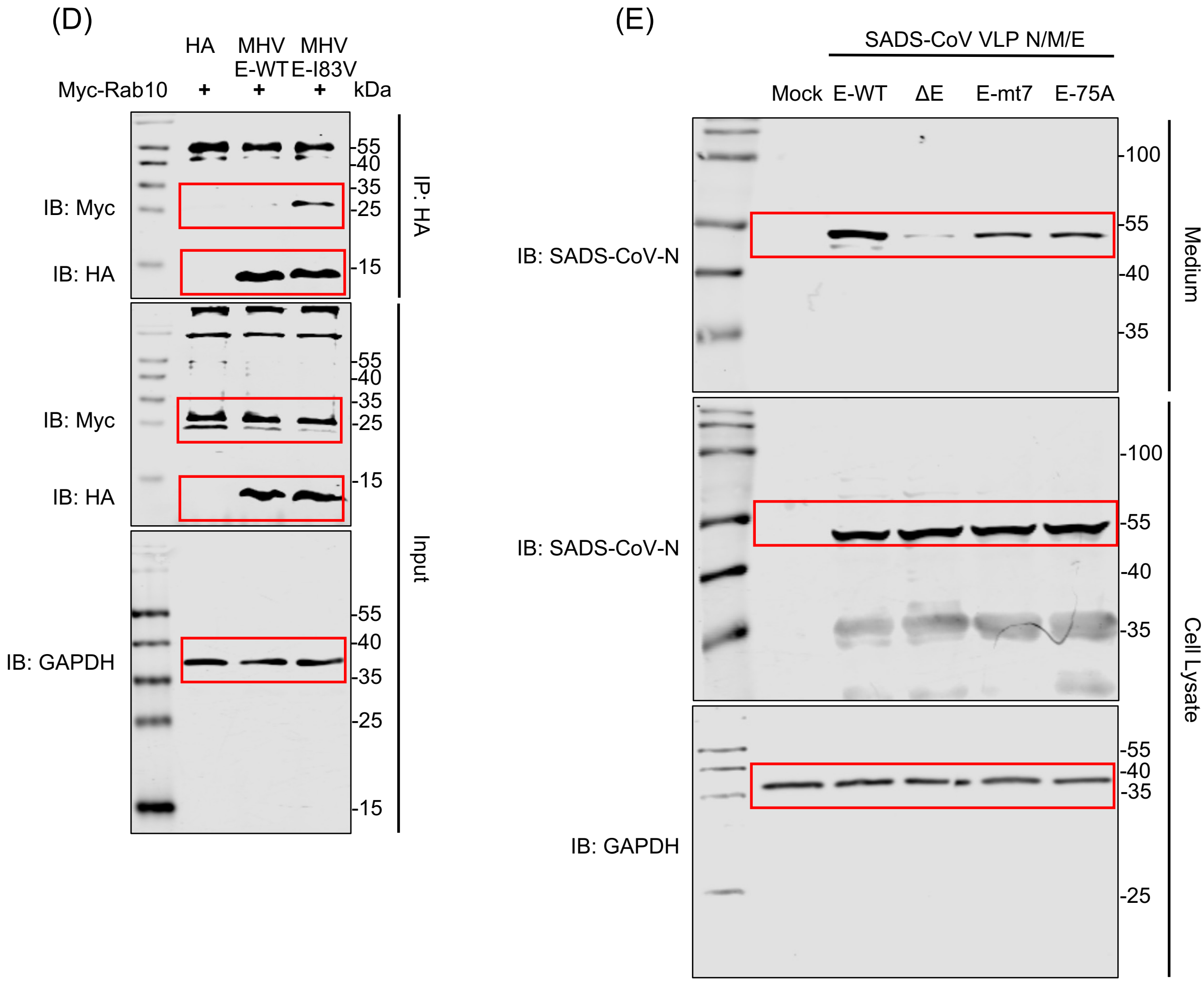

Uncropped gels for Western Blots in Fig 11.

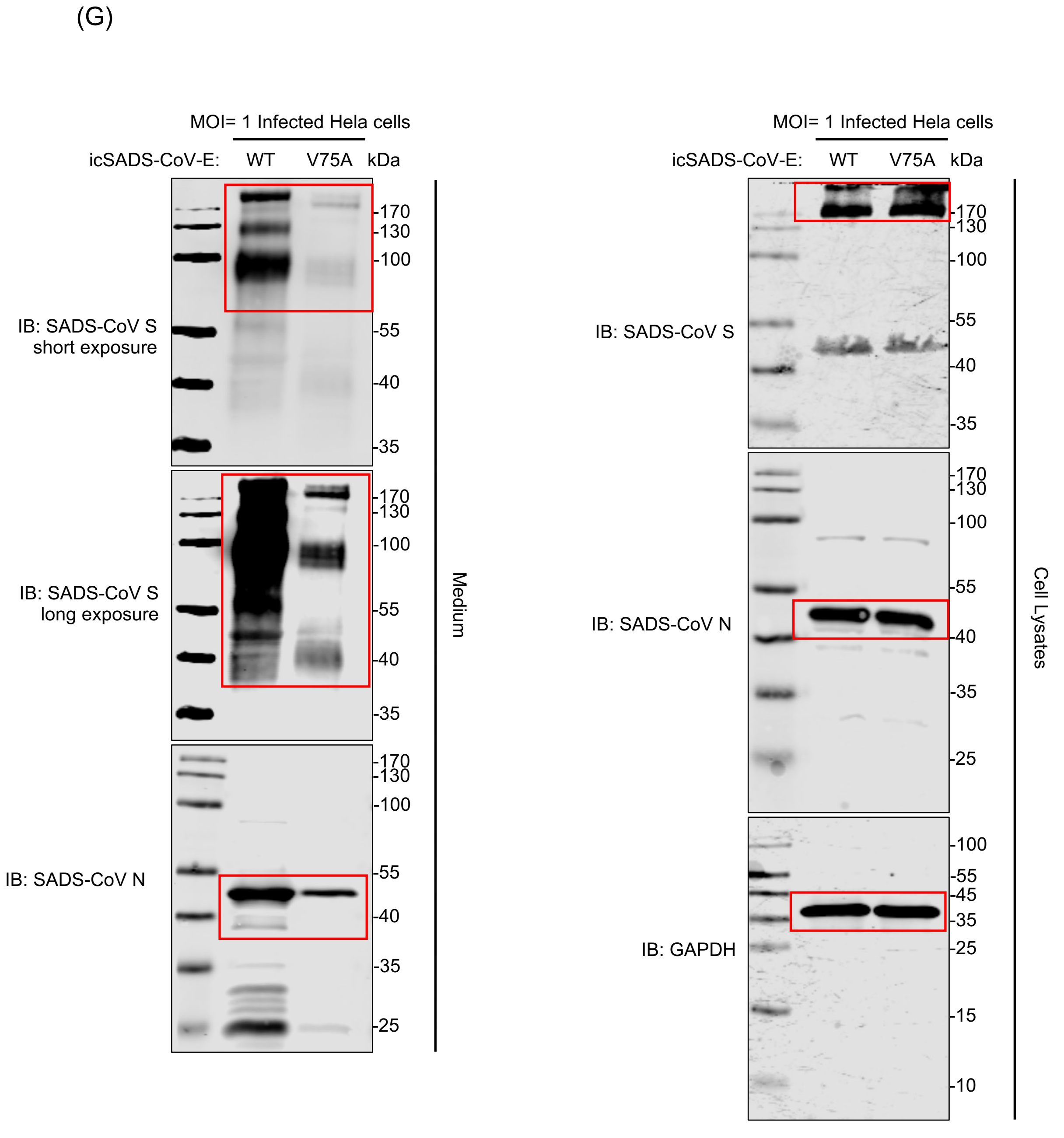

Uncropped gels for Western Blots in S1 Fig.

(E)

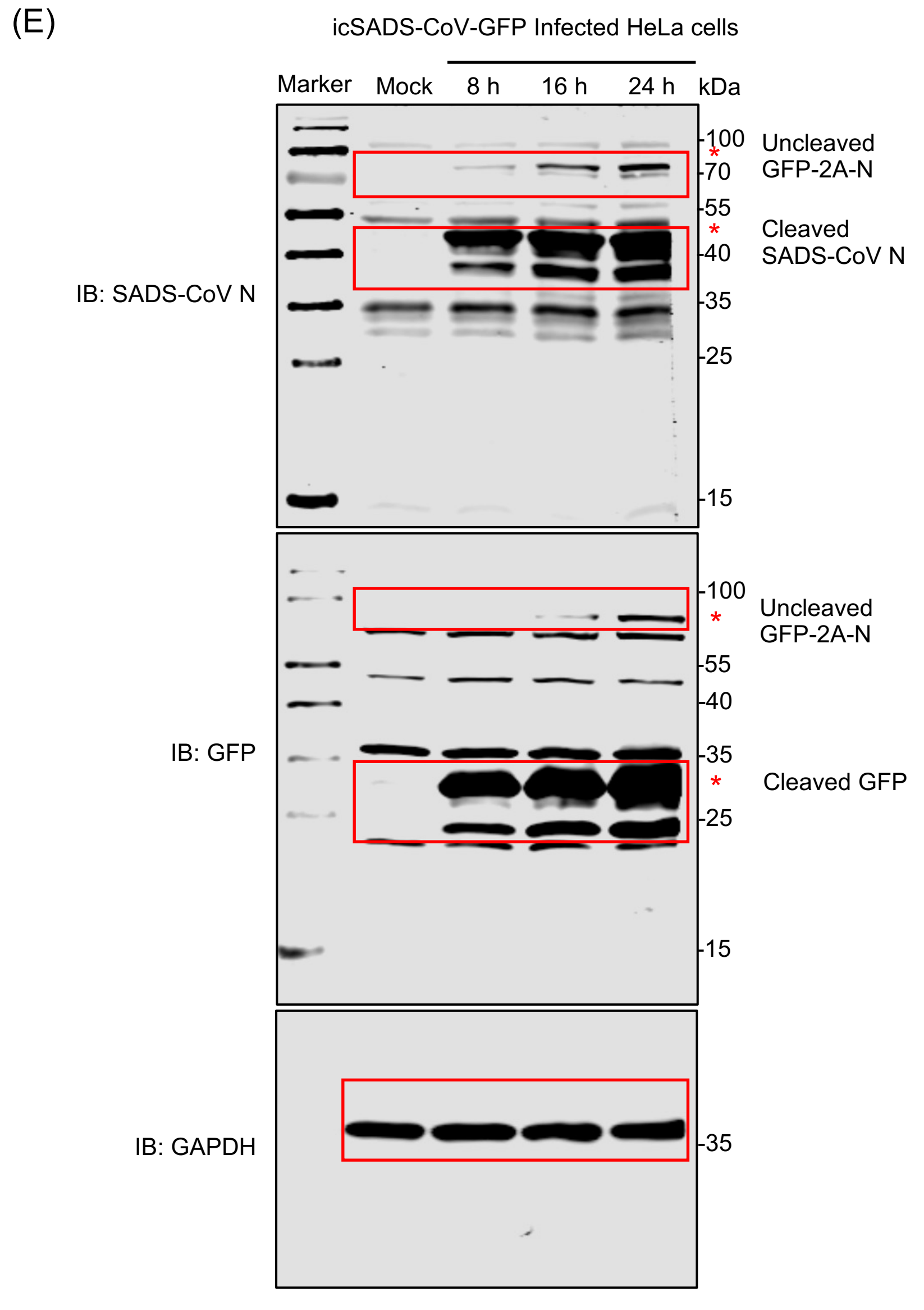

Uncropped gels for Western Blots in S2 Fig.

(A)

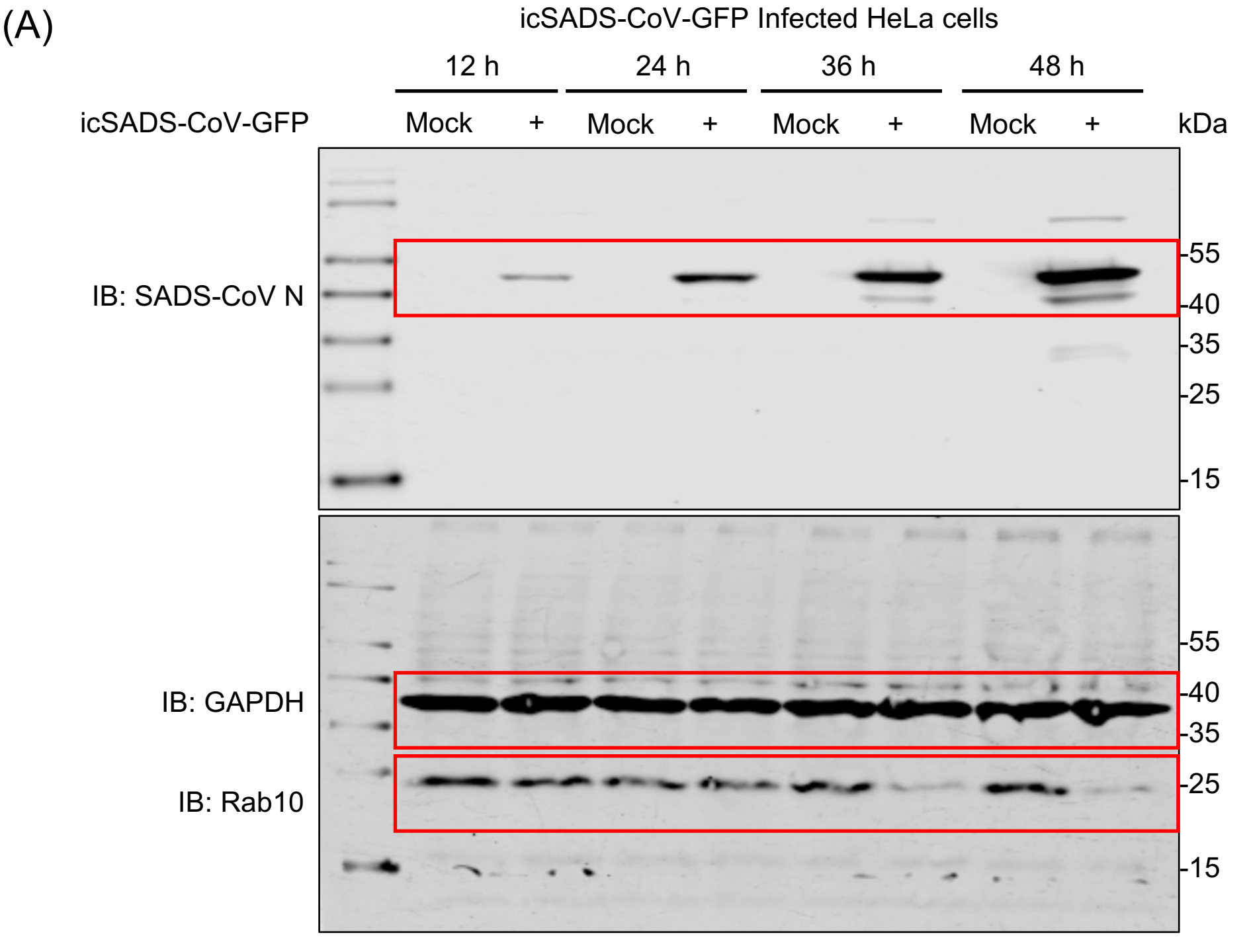

Uncropped gels for Western Blots in S3 Fig.

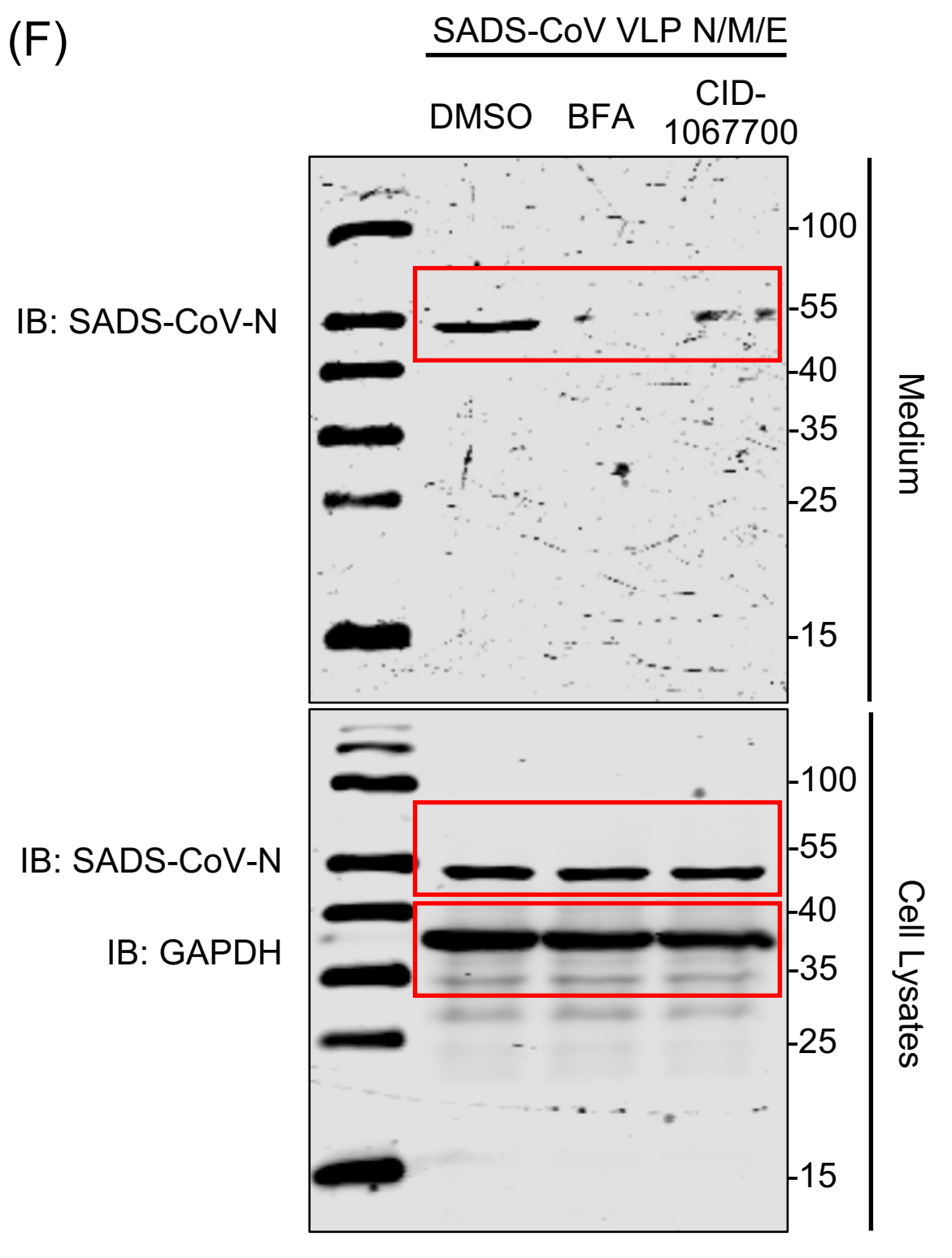

Supplement: Supplementary file 1 [file ppat.1014569.s008.pdf]
